# Icotinib derivatives as tyrosine kinase inhibitors with anti-esophageal squamous carcinoma activity

**DOI:** 10.3389/fphar.2022.1028692

**Published:** 2022-11-16

**Authors:** Xiaojie Chen, Long-Fei Mao, Siqi Tian, Xueli Tian, Xueqiong Meng, Mu-Kuo Wang, Weifeng Xu, Yue-Ming Li, Kangdong Liu, Zigang Dong

**Affiliations:** ^1^ School of Basic Medical Sciences, Henan University of Science and Technology, Luoyang, China; ^2^ The First Affiliated Hospital of Henan University of Science and Technology, Luoyang, China; ^3^ China-US (Henan) Hormel Cancer Institute, Zhengzhou, China; ^4^ State Key Laboratory of Medicinal Chemical Biology, College of Pharmacy and Tianjin Key Laboratory of Molecular Drug Research, Nankai University, Haihe Education Park, Tianjin, China; ^5^ School of Chemistry and Chemical Engineering, Henan Normal University, Henan Engineering Research Center of Chiral Hydroxyl Pharmaceutical, Xinxiang, China; ^6^ Department of Medical Oncology, Affiliated Cancer Hospital of Zhengzhou University, Henan Cancer Hospital, Zhengzhou, China; ^7^ Department of Pathophysiology, School of Basic Medical Sciences, Zhengzhou University, Zhengzhou, China

**Keywords:** icotinib, 1,2,3-triazole, NSCLC, ESCC, EGFR-TK pathway

## Abstract

Previous report showed that a variety of icotinib derivatives bearing different 1,2,3-triazole moieties, which could be readily prepared *via* copper (I)-catalyzed cycloaddition (CuAAC) reaction between icotinib and different azides, exhibited interesting activity against different lung cancer cell lines such as H460, H1975, H1299, A549 or PC-9. To further expand the application scope of the compounds and to validate the function of triazole groups in drug design, the anti-cancer activity of these compounds against esophageal squamous carcinoma (ESCC) cells was tested herein. Preliminary MTT experiments suggested that these compounds were active against different ESCC cell lines such as KYSE70, KYSE410, or KYSE450 as well as their drug-resistant ones. Especially, compound 3l showed interesting anticancer activity against these cell lines. The mode of action was studied *via* molecular docking, SPR experiments and other biochemical studies, and 3l exhibited higher binding potential to wild-type EGFR than icotinib did. *In vivo* anticancer study showed that 3l could inhibit tumor growth of cell-line-derived xenografts in ESCC. Study also suggested that 3l was a potent inhibitor for EGFR-TK pathway. Combining these results, 3l represents a promising lead compound for the design of anti-cancer drugs against ESCC.

## Introduction

EGFR is a member of human epidermal growth factor receptor (HER) family locating on the short arm of human chromosome 7. It regulates the response of cells to external stimuli through a series of reactions and participates in the growth and development of various tissues and organs (Lee, 2017; Al-Quteimat and Amer, 2020).The overexpression of EGFR was related to the occurrence and development of lung cancer (Russo et al., 2020), breast cancer (Loibl and Gianni, 2017), glioma (Kros et al., 2015) or esophageal cancer (Huang et al., 2016), and EGFR-tyrosine kinase inhibitors are currently the first-line therapeutics for the treatment of advanced non-squamous cell carcinoma with EGFR mutation. Especially, EGFR-TKIs can improve the local control rate, prolong the tumor progression-free stage and the overall survival period, and improve the life quality of these patients (Jia et al., 2016).

Esophageal cancer has been identified as a tumor with generally high expression of epidermal growth factor receptor (EGFR) (Lyu et al., 2014; Liu et al., 2018). Moreover, EGFR overexpression is closely related to tumor invasion, metastasis and chemoradiotherapy tolerance of esophageal cancer (Zhang et al., 2016; Xu et al., 2018). Numerous EGFR monoclonal antibodies or EGFR tyrosine kinase inhibitors (Meluch et al., 2010; Sutter et al., 2010) have been used alone or in combination with chemotherapy, radiotherapy or chemoradiotherapy to treat esophageal cancer, but the results have been disappointing (Ren et al., 2018). Due to the insidious symptoms of early esophageal cancer and low prevalence of endoscopic screening, most of the patients were in the advanced stage at the initial diagnosis (Mao et al., 2020a). The efficacy of chemoradiotherapy in patients with non-operative esophageal cancer has reached the bottleneck, and the overall 5-year survival rate is about 30% (Asokan et al., 2020; Then et al., 2020). EGFR was not or weakly expressed in chronic esophageal mucosal inflammation tissues with a positive expression rate of 23.33%, but 67.50% in esophageal squamous cell carcinoma tissues (Eskilsson et al., 2018). The expression of EGFR was related to TNM staging of ESCC (Eskilsson et al., 2018). These findings suggest that EGFR-TKIs may be suitable as a starting point for the design of new chemical entities against esophageal cancers.

We have shown that new compounds with promising anti-cancer activity could be obtained after introducing 1,2,3-triazole moieties to icotinib. Such compounds showed interesting activity against different lung cancer cell lines such as H460, H1975, H1299, A549 or PC-9 (Mao et al., 2020b). In this paper, we wish to further expand the application scope of the compounds against ESCC cells.

## Materials and methods

### Chemistry

The synthetic route to target compounds 3 is outlined in Scheme 1 using compound 1 as starting material. Icotinib with different azides provided the desired compounds 3a-3v in good yields *via* click reaction. The structures of the key intermediates and all the products were confirmed by nuclear magnetic resonance (Supplementary Figures S1–S22, Mao et al., 2020b).

**SCHEME 1:**
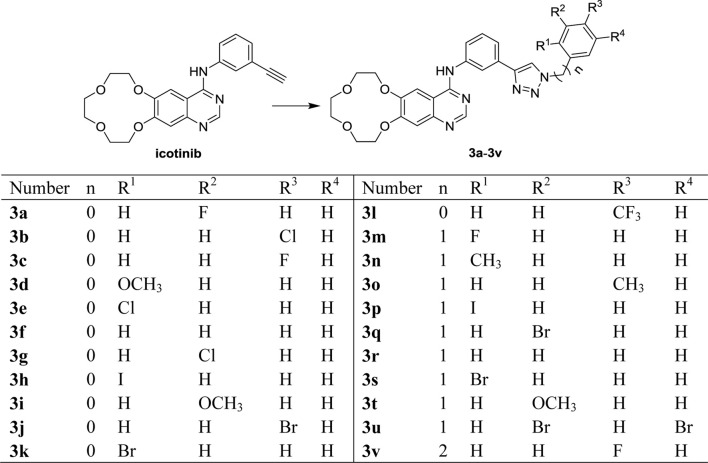
Synthetic route to icotinib-1,2,3-triazole derivatives **3**.

### Docking simulation

*In silico* docking was carried out using AutoDock 4.2 (Rizvi et al., 2013). The crystal structure of human epidermal growth factor receptor (1M17) was downloaded from the protein data bank (https://www.rcsb.org/). Pymol was used to remove all water, ligands and co-factors. AutoDockTools (Morris et al., 2009) was used to add hydrogen, calculate Gasteiger charges, and generate PDBQT files of compounds and receptors. A grid of 54, 54, and 54 points in x, y, and z directions was built with a grid spacing of 0.375 Å and a distance-dependent function of the dielectric constant was used for the calculation of the energetic map. The default settings were used for all other parameters. The Lamarckian genetic algorithm method (Morris et al., 1998)was employed for docking simulations. The standard docking procedure was used for a rigid protein and a flexible ligand whose torsion angles were identified (for 200 independent runs per ligand).

### Cell culture and treatment

The human cancer cells (H1650, H1975, KYSE70, KYSE410 and KYSE450) were purchased from the Type Culture Collection of the Chinese Academy of Sciences (Shanghai, China). The human immortalized normal esophageal epithelial cell line SHEE was donated by Dr. Enmin Li from the Laboratory of Tumor Pathology (Shantou University Medical College, Shantou, Guangdong, China). Drug-resistant cells (H1650TR, H1975OR, KYSE70TR, KYSE410TR and KYSE450TR) were induced by China-US (Henan) Hormel Cancer Institute. The cellswere cultured in RPMI-1640 complete growth medium containing 100 U/mL penicillin-streptomycin and 10% FBS. Based on the above medium formula, 100 nm/L paclitaxel was added in taxol-resistant cells, 100 nm/L gefitinib was added in gefitinib-resistant cells. The cells were incubated at 37 °C in an atmosphere containing 5% of CO_2_. The compounds were dissolved in DMSO to make a 50 mM stock solution and were diluted to the concentration of working solutions with complete growth medium before administration.

### MTT assay for cell proliferation and cytotoxicity

Cells were seeded in 96-well plates with 2,200–2,500 cells/well in100 μL. One day after seeding, test compounds with the concentration between 0 and 50 μM were added with 0.1% DMSO as control. Five multiple wells for each concentration. After 48 h, MTT was added and incubated in the plate for 1–4 h in the incubator. The absorbance at 490 nm was measured using a microplate reader (Thermo).

### Culture assay for tumor colony-forming cells

Compounds to be tested were suspended in 0.5% agar in RPMI-1640 medium, supplemented with 10% FBS, 0.1% gentamicin, 9% PBS, 1% l-glutamine. Concentrations of compounds ranged from 2 μM to 16 μM. Just prior to plating, 3 ml mixture was pipetted into one well of six-well plates. After the coagulation of the lower agar, cells to be tested were suspended in the mixture, the final concentration of cells was 8000 cells in 1 ml of agar medium. One mL mixture was pipetted into one well with three multiple holes in each group. After preparation of both bottom and top layers, the plates were examined under the inverted microscope to assure the presence of a good single-cell suspension. The plates were then incubated at 37°C in a 5% CO_2_ humidified atmosphere.

### Scoring and identification of colonies in cultures

Cultures were examined with a Nikon inverted-phase microscope at × 40. Colony counts were made 15 days after plating. Aggregates of 50 or more cells were considered colonies. Aggregates of less than 50 cells were considered clusters. The plates were placed under an inverted microscope, and 10 visual fields were chosen at random, and the number of colonies greater than 50 cell or 0.05 mm in field of vision were counted.

### CFSE assay for cell proliferation and cytotoxicity

Mother liquor (1 ×)was prepared in advance from CFSE (CytoTell™ Red 650, cat. No. 22255) in DMSO according to the instructions, and stored separately in −80°C refrigerator. Cell was planked with 12-well plates, 2×10^4^ cells for each well. After 24 h, cells were added into CFSE solution at 1 : 500 ratio and incubated in darkness for 15 min at 37°C. The cells was washed three times with cold culture medium containing 10% FBS. Cells were treated with **3l** or icotinib for 48 h at 3 μM concentrations using 0.1% of DMSO as the control. Finally, the cells were detected or observed using flow cytometry or confocal microscopy.

### EGFR kinase assay

Kinase inhibitory activities of the compounds were evaluated using the Protein Tyrosine Kinase Assay Kit (SignalChem (British Columbia, Canada)). The kinase enzyme of EGFR was purchased from Active MOTIF. The ELISA experiments were carried out according the instructions of the kit. Active kinases were incubated with indicated compounds in 1 × reaction buffer containing 20 μM substrate at 25°C for 1 h and 2 h, respectively. The absorbance was read with a multimode plate reader (PerkinElmer, United States) at 450 nm.

### Flow cytometry detection for cell apoptosis

Cell-apoptosis analysis was carried out by flow cytometry using the Annexin V/PI apoptosis methods. Briefly, KYSE450/KYSE450TR (2 × 10^4^ to 3× 10^4^/well) cells were incubated in 6-well plates for 12 h, and then treated with 0.1% DMSO (as control), compound **3l** or icotinib at various concentrations for 48 h, respectively. Cells were harvested and incubated with 250 μL of 1 × Annexin V binding buffer containing 5 μL PI and 5 μL FITC Annexin V (final concentration 1.8 μg/ml, Biolegend (cat: 640945) for 15 min at room temperature in dark. 200 µL of 1 × binding buffer was added for flow cytometry analysis (BD FACSCalibur™ Flow Cytometer).

### Cell cycle analysis

KYSE450/KYSE450TR cells were plated in 6-well plates with a density of 1×10^5^ cells/well and cultured overnight. Cells were treated with **3l** or icotinib of different concentrations (0, 2, 4, 8, 16, 32 μM or 0, 0.5, 1, 2, 4, 8 μM) for 24 h. Trypsinization treatment and the cells were collected by centrifugation. The cell pellet was re-suspended in 70% ethanol at −20°C for at least 3 h. The cells were washed with PBS and were re-suspended in 250 μL of 0.6% tricine with Renease A for 1 h, then stained in PI (final concentration 1.8 μg/ml, Biolegend cat: 640945) for 15 min in dark. Cells were retransferred to the BD FACSCalibur™ Flow Cytometer. All analyses were performed with FlowJo software v105.3.6.

### EGFR protein affinity determined with SPR

Firstly, EGFR protein (protein concentration : 0.468 μg/μL, Active MOTIF, cat: 31165, lot: 28215004) was covalently immobilized onto a CM5 sensor chip at densities of 2000 response units. Then, **3l** and icotinib dissolving in DMSO was injected at concentrations between 0.064 nM and 125000 nM at 25°C. The final doses of DMSO did not exceed 1% (v/v). During the drug-protein interaction, the change of the refractive index was measured in real-time which would allow the plotting of the results of interaction as response units *versus* time. The interaction results were analyzed under a BIA evaluation 3.0 software.

### Western blot analysis

KYSE450/KYSE450TR cells (3 × 10^5^/well) were incubated overnight in six-well plates, and then treated with either compound **3l** or icotinib at 3 μM for 0, 0.5, 1, 2, 3 and 6 h. Cells treated with 1% DMSO were used as control. Then the cells were harvested and total proteins were extracted. Total proteins were separated by 12% SDS polyacrylamide gel electrophoresis and transferred onto PVDF membranes. The membrane was blocked for 1 h, then incubated overnight with a 1 : 1000 dilution of anti-EGFR, anti-p-EGFR (Tyr1068/Tyr1086), anti-Erk1/2, and anti-p-Erk1/2 (Thr202/Tyr204), or 1 : 3000 dilution of anti-GAPDH primary antibody at 4°C. 1 : 3000 anti-rabbit secondary antibodies incubated for 2 h at room temperature. Protein bands were developed by chemiluminescence.

### *In vivo* cell-derived xenograft mouse model

Mice were housed under specific pathogen-free conditions. All animal experiments were approved by the Bioethics Committee of Zhengzhou University and followed the guidelines set by the Institutional Animal Care and Use Committee (CUHCI2019002, CUHCI2021001, and CUHCI2021005). To generate the cell-derived xenograft (CDX) mouse model, nu/nu nude mice (Vital River Labs., Beijing, China) were subcutaneously injected with 5 × 10^6^ cells (KYSE450). Mice were randomly divided into four groups: 1) control, 2) erlotinib, 3) icotinib and 4) **3l**.Once tumor volumes reached approximately 200 mm^3^, compounds (50 mg/kg)or vehicle (5% DMSO in PBS) was given by intraperitoneal injection every day. Tumor volume was calculated as follows: length × width × height × 0.5. Tumor size and mice weight were measured every 2–3 days. Compounds were administered for 15 days. The tumors were extracted for weighting, immunohistochemistry and immunofluorescence studies.

### Immunofluorescence

Frozen sections were washed three times with PBS and incubated overnight at 4°C with Ki-67 antibody. The next day, sections were washed three times and incubated with secondary antibody in PBS. After several washes, slides were counterstained with DAPI (Solarbio). The sections were mounted on microscope and imaged using fluorescent microscopy (Nikon, Tokyo, Japan). Image-Pro Plus 6.0 was used to analyze the fluorescence intensity.

### Immunohistochemical analysis

Histological sections were dewaxed, antigens were repaired and sealed with serum in sequence. Next, histological sections were incubated overnight with diluted primary antibodies (1 : 500 dilution), and the control specimens were treated with PBS. The sections were washed 3 times with PBS. Secondary antibody was added and the system was then placed at 37 °C for half an hour. The systems were washed 3 times with PBS. Color developing agent was added. After washing with water, the system was soaked in hematoxylin for 0.5 min for dyeing. The re-dyed slides were washed with water, and then dehydrated. Neutral gums were dropped on the side of the tissue. The slices were then covered with cover glass, sealed and placed in a ventilator for drying. Immunohistochemical staining analysis was then carried out with Image-Pro Plus 6.0.

### Statistical analyses

Statistical analysis was conducted using Graph Prim 5.0. Statistical probability (P) for significance was expressed as *p < 0.05, **p < 0.01 and ***p < 0.001. One- or two-way analysis of variance (ANOVA) or Unpaired Student’s t-test was used to assess statistical significance between datasets. Error bars represent the mean ± SD. The details of the statistical tests carried out are indicated in the respective figures.

## Results

### Docking simulation

**FIGURE 1:**
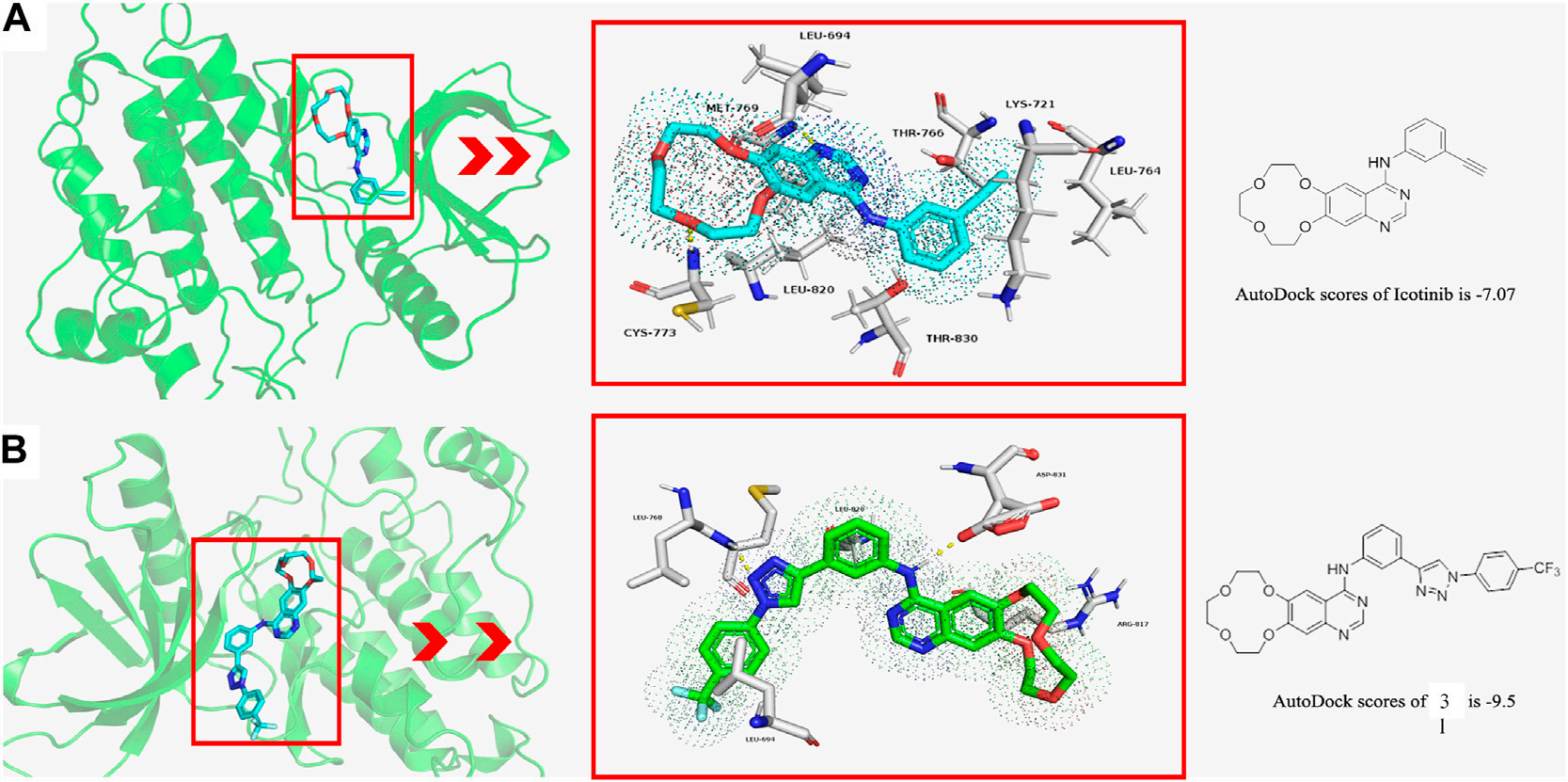
The binding modes of icotinib and compound 3l with EGFR (PDB: 1M17) **(A)** The binding mode of icotinib in the ATP binding site of EGFR. The AutoDock score for icotinib is −7.07 **(B)** The binding mode of 3l in the ATP binding site of EGFR. The AutoDock score for 3l is −9.5.

Molecular docking studies are conducted to explore the binding modes of icotinib and compound **3l** in the active site of EGFR, and the results are presented in Figure 1. Figure 1A shows the interaction of icotinib with EGFR and Figure 1B shows the interaction of **3l** with EGFR. Preliminary results suggested icotinib or **3l** interacted with the ATP binding site of EGFR with docking score of −7.07 kcal/mol and −9.5 kcal/mol, respectively.

Icotinib could occupy the pocket containing LEU 694, LYS 721, LEU 764, THR 766, LEU 820 and THR 830. The backbone amino group of CYS 773 formed hydrogen bond with one oxygen atom of the crown ether group, and amino group of MET 769 formed hydrogen bond with quinazoline moiety of icotinib (Figure 1A). Compound **3l** could occupy the pocket containing LEU 694, ARG 817 and LEU 820. The backbone amino group of LEU 768 formed hydrogen bond with one nitrogen atom of the 1,2,3-triazole group. The oxygen atom of ASP 831 formed hydrogen bond with the quinazoline moiety of **3l** (Figure 1B).

### Inhibition of NSCLC and ESCC cells proliferation by icotinib-1,2,3-triazole derivatives

In our previous report, we have shown that compounds 3a-3v were effective against several lung cancer cell lines such as H460, H1975, H1299, A549 or PC-9 (Mao et al., 2020b).To verify the effectiveness of these compounds against lung cancer cells, human NSCLC cell lines H1650, and H1975 were chosen for further study. While H1650 expressed wild type EGFR, H1975 possessed the L858R and T790M mutation associating with first-generation EGFR-TKIs resistance (Naruse et al., 2002). Drug resistance cells H1650TR and H1975OR were also tested. H1650TR cells are resistant to taxol, and H1975OR cells are resistant to the recent EGFR-TKI osimertinib. The resistant indices are shown in Table 1. Cytotoxicity of compounds 3a-3v against different NSCLC cell lines are shown in Table 2. Preliminary results suggested that EGFR wild-type lung cancer cell H1650 and EGFR mutant lung cancer H1975 cells showed poor sensitivity to icotinib with IC50 values of >50 μM, and icotinib-triazole derivatives such as 3b, 3d, 3e, 3g, 3i, 3k, 3l, 3n, 3o and 3v all exhibited stronger killing effects on the above lung cancer cell lines than icotinib did.

**TABLE 1:** The resistance indices of the drug-resistant cell lines (n = 5).

Cell	24 hIC_50_ (nM)	Resistance index	48 hIC_50_ (nM)	Resistance index
H1650	870.07 ± 75.70	4.74	83.33 ± 3.33	7.74
H1650TR	4126.12 ± 264.00		645.03 ± 45.03	
H1975	584.77 ± 26.13	23.57	102.11 ± 12.12	58.48
H1975OR	13782.14 ± 2976.32		5972.30 ± 289.18	

Conditions: The absorbance at 490 nm of MTT, were measured using a microplate reader (Thermo). Data are shown as mean ± SD.

MTT, method was used for preliminary assessments. The cytotoxicity of compounds 3a-3v against these NSCLC, cell lines are shown in Table 2.

**TABLE 2:** Cytotoxicity of compounds 3a-3v against different NSCLC cell lines (n = 5).

Nr	IC_50_ (μM) 48 h		Nr	IC_50_ (μM) 48 h	
	H1650	H1975		H1650	H1975
3a	15.60 ± 1.02	9.87 ± 0.88	3l	4.40 ± 0.32 (8.43 ± 0.68)	3.26 ± 0.26 (9.73 ± 0.83)
3b	7.81 ± 0.46	>50	3m	>50	47.33 ± 2.01
3c	9.40 ± 0.73	>50	3n	17.00 ± 1.27	18.52 ± 1.43
3d	10.8 ± 0.89	19.41 ± 1.21	3o	5.60 ± 0.29	8.70 ± 0.66
3e	5.61 ± 0.30	13.11 ± 1.02	3p	4.41 ± 0.31	39.52 ± 1.91
3f	12.30 ± 0.91	>50	3q	21.50 ± 1.87	47.27 ± 2.13
3g	8.72 ± 0.73	4.75 ± 0.33	3r	19.62 ± 1.72	19.15 ± 1.59
3h	30.62 ± 1.99	12.13 ± 0.92	3s	17.10 ± 1.64	>50
3i	6.25 ± 0.54	19.70 ± 1.07	3t	21.01 ± 1.91	20.02 ± 1.52
3j	27.63 ± 1.85	5.02 ± 0.37	3u	>50	>50
3k	17.71 ± 1.21	27.18 ± 1.67	3v	11.60 ± 0.96	11.12 ± 0.95
icotinib	>50	48.27 ± 2.13	—	—	—
—	(>50)	(>50)	—	—	—

Conditions: The absorbance at 490 nm of MTT, were measured using a microplate reader (Thermo). Data are shown as mean ± SD.

As a consequence, the single use or combined use of EGFR signaling pathway blockers with chemoradiotherapy has recently become one of the hotspots in EC research. Thus, esophageal cancer cell lines KYSE 70, KYSE410, and KYSE450 were chosen to test the bioactivity of the compounds in an attempt to expanding the application scope of these compounds. The taxol-resistant cell lines such as KYSE70TR, KYSE410TR and KYSE450TR were also subjected to the same study. The resistance indices of these cell lines were listed in Table 3. The human immortalized esophageal epithelial cell line (SHEE) was used as a control to get preliminary information about cytotoxicity of the compounds against normal cells. The results are shown in Table 4. Icotinib-triazole derivatives such as 3b, 3d, 3g, 3k, 3l, 3n, 3r and 3v all exhibited stronger inhibition effects on the above three ESCC cell lines than icotinib did.

**TABLE 3:** The resistance indices of KYSE70TR, KYSE410TR and KYSE450TR cell lines (n = 5).

Cell	24 hIC_50_ (nM)	Resistance index	48 hIC_50_ (nM)	Resistance index
KYSE 70	640.07 ± 43.00	7.69	20.21 ± 2.19	56.00
KYSE 70 TR	4924.27 ± 274.00		1120.33 ± 35.16	
KYSE 410	1280.09 ± 69.00	5.00	52.41 ± 3.45	32.88
KYSE 410 TR	6400.43 ± 430		1710.05 ± 50.04	
KYSE 450	55.03 ± 3.82	4.84	7.82 ± 0.84	15.38
KYSE 450 TR	266.13 ± 66.14		120.32 ± 30.14	

Conditions: The absorbance at 490 nm of MTT, were measured using a microplate reader (Thermo). Data are shown as mean ± SD.

**TABLE 4:** Antiproliferative activities of compounds 3a-3v against ESCC cell lines (n = 5).

Nr	IC_50_ (μM) 48 h				Nr	IC_50_ (μM) 48 h			
	SHEE	KYSE70	KYSE410	KYSE450		SHEE	KYSE70	KYSE410	KYSE450
3a	12.14 ± 0.97	10.22 ± 0.88	48.57 ± 2.16	7.24 ± 0.65	3l	4.82 ± 0.23	4.25 ± 0.21 (5.13 ± 0.23)	5.94 ± 0.41 (5.30 ± 0.28)	3.40 ± 0.16 (8.59 ± 0.78)
3b	6.03 ± 0.48	8.85 ± 0.67	10.67 ± 0.86	11.60 ± 1.02	3m	>50	>50	>50	>50
3c	6.23 ± 0.37	5.84 ± 0.36	>50	13.81 ± 1.13	3n	8.05 ± 0.71	6.87 ± 0.51	8.23 ± 0.92	13.37 ± 0.99
3d	7.20 ± 0.71	7.35 ± 0.70	8.67 ± 0.71	13.47 ± 1.21	3o	22.35 ± 1.26	14.60 ± 0.96	>50	4.81 ± 0.36
3e	10.33 ± 0.84	7.75 ± 0.68	11.68 ± 1.01	21.84 ± 1.47	3p	>50	19.92 ± 1.31	>50	>50
3f	>50	25.04 ± 1.53	>50	45.03 ± 2.53	3q	>50	20.71 ± 1.73	>50	21.80 ± 1.12
3g	8.01 ± 0.77	6.13 ± 0.31	10.88 ± 0.99	4.51 ± 0.29	3r	20.99 ± 1.11	7.32 ± 0.62	9.23 ± 0.97	28.11 ± 1.18
3h	22.59 ± 1.19	18.43 ± 1.05	>50	16.70 ± 1.31	3s	14.17 ± 0.94	9.35 ± 0.89	19.22 ± 1.22	23.35 ± 1.13
3i	28.08 ± 1.38	7.02 ± 0.64	>50	10.02 ± 0.93	3t	33.26 ± 1.99	>50	>50	45.62 ± 2.01
3j	>50	50	>50	12.52 ± 1.22	3u	>50	>50	>50	>50
3k	12.52 ± 1.03	5.05 ± 0.34	21.87 ± 1.39	6.25 ± 0.57	3v	8.26 ± 0.71	6.93 ± 0.37	21.55 ± 1.35	11.04 ± 0.97
icotinib	19.16 ± 1.29	>50	>50	30.4 ± 1.96	—	—	—	—	—
—	—	(>50)	(>50)	(>50)	—	—	—	—	—

Conditions: The absorbance at 490 nm of MTT, were measured using a microplate reader (Thermo). Data are shown as mean ± SD.

**FIGURE 2:**
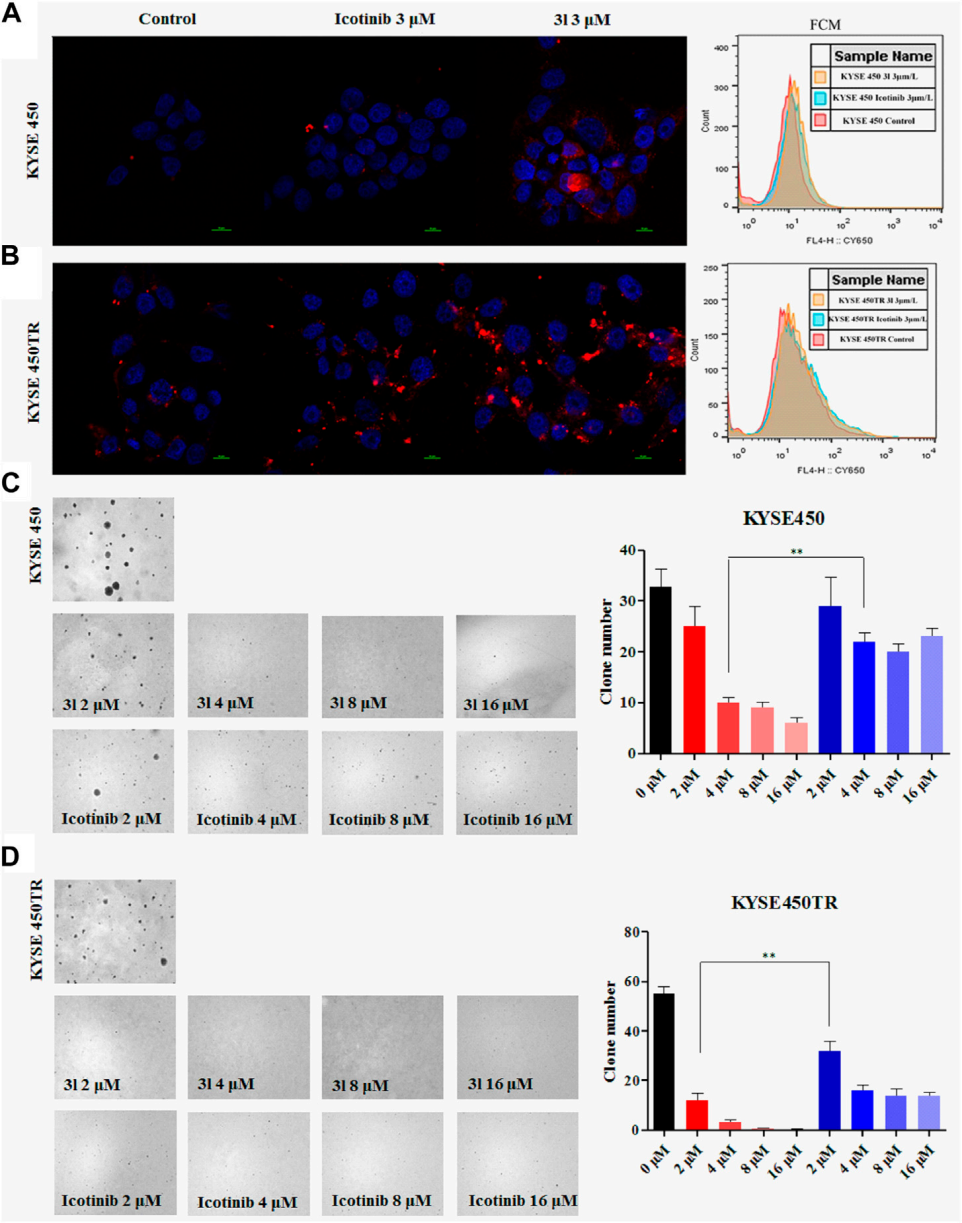
Carboxyfluorescein succinimidyl ester dilution assay and the soft-agar assay results **(A)** and **(B)** The results of CSFE. The proliferation inhibition of 3l and icotinib on KYSE450 and KYSE450TR cells compared with cells treated with 0.1% DMSO **(C)** and **(D)** The results of soft-agar. The proliferation inhibition of 3l and icotinib on KYSE450 and KYSE450TR cells. Unpaired Student’s t test was used in **(C)** and **(D)**. *p < 0.05, **p < 0.01, ***p < 0.001. Error bars represent the mean ± SD.

Similar results were obtained in soft-agar method (Von Hoff et al., 1980) when 3l was tested against KYSE450 (Figure 2C, at 4 μM, Control VS. 3l,n = 3,F = 0.221, t = −5.868, *p* = 0.004)and KYSE450TR (Figure 2D, at 2 μM, Control VS. 3l,n = 3, F = 0.727, t = -15.495, *p* = 0.002). To further verify the proliferation inhibition of 3l and icotinib against ECSS cells, cells were stained with carboxy fluorescein succinimidyl (CSFE) and were treated with 3l or icotinib for 48 h at 3 μM concentrations using 0.1% of DMSO as a control. Cells were observed with a fluorescence microscope and flow cytometry. Similar results were observed in CFSE experiments when compounds 3l and icotinib were tested against KYSE450/KYSE450TR (Figures 2A, B). The results of plate clone were given in (Supplementary Figure S23).

### Cell death induced by icotinib-1,2,3-triazole derivatives *via* cell cycle arrest

**FIGURE 3:**
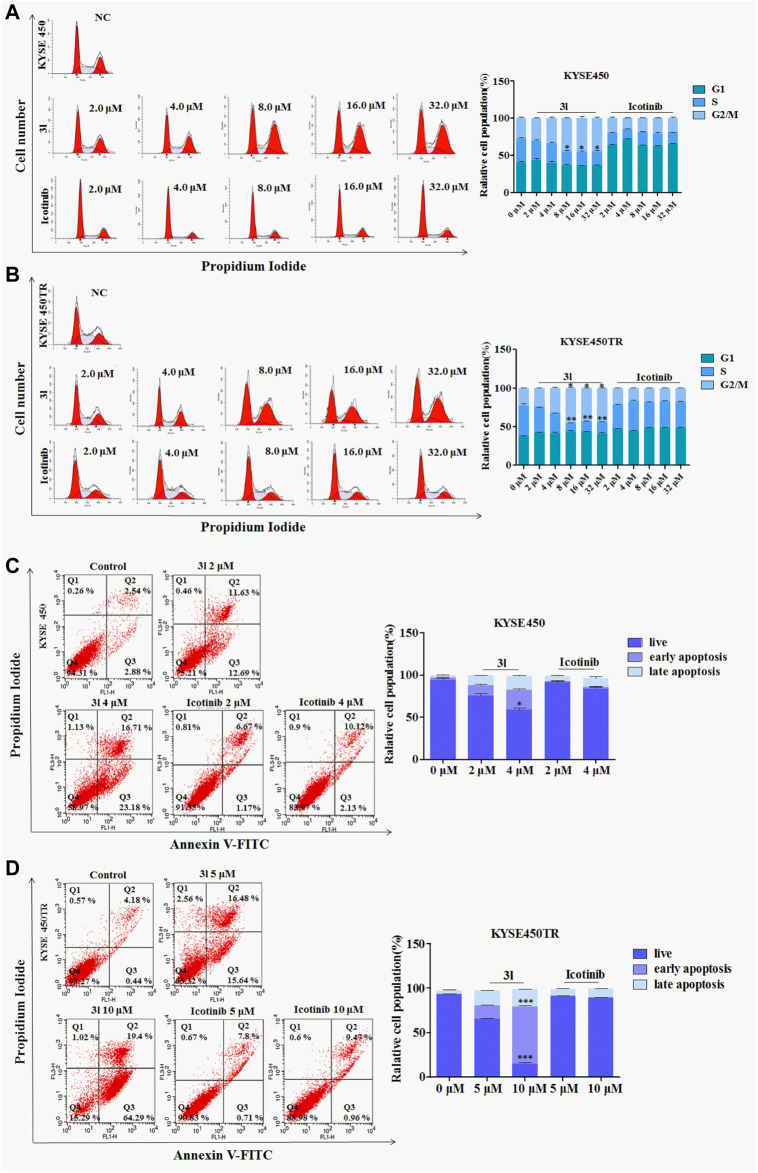
The result of apoptosis and cell cycle in ESCC cell lines induced by 3l and icotinib **(A)** and **(B)** The cell cycle of KYSE450 and KYSE450TR cells by 3l and icotinib, compared with cells treated with 0.1% DMSO **(C)** and **(D)** The apoptosis of KYSE450 and KYSE450TR cells by 3l and icotinib, compared with cells treated with 0.1% DMSO. Unpaired Student’s t test was used. *p < 0.05, **p < 0.01, ***p < 0.001. Error bars represent the mean ± SD.

To investigate the effects of 3l on the various phases of the cell cycle, KYSE450/KYSE450TR cells were treated with various concentrations of 3l for 24 h, and analyzed by flow cytometry using 0.1% DMSO as a control. The results are presented in Figure 3. As shown in Figures 3A, B, icotinib arrested KYSE450 cells in G_1_ phase at low concentration (2 μM) for 24 h. Compound 3l arrested KYSE450 cells in G_2_/M phase at a low concentration (8 μM) for 24 h (Figure 3A, Control Vs. 3l, n = 3, F = 0.000, t = −4.009, *p* = 0.016). Icotinib arrested KYSE450TR cells in G1 phase at low concentration (8 μM), and 3l arrested KYSE450TR cells in G2/M phase at low concentration (8 μM) (Figure 3B, Control Vs. 3l, n = 3, F = 0.450, t = −8.910, *p* = 0.007).

### Apoptosis in different ESCC cell lines induced by icotinib-1,2,3-triazole derivatives

To clarify whether the inhibitory effects of 3l on cell proliferation were related to apoptosis, one ESCC cell lines and its corresponding drug resistant cells were chosen for further experiments. KYSE450/KYSE450TR cells were treated with DMSO or different concentrations of icotinib or 3l for 48 h, cells were stained with Annexin-V and PI, and the proportion of apoptotic cells was detected by flow cytometry. As shown in Figures 3C, D, the proportions of KYSE450 apoptotic cells treated with 3l were 24.32% (2 μM) and 39.89% (4 μM) (Figure 3C, at 4 μM, Control Vs. 3l, n = 3, F = 1.798, t = −13.936, *p* = 0.003). The proportions of KYSE450 apoptotic cells treated with icotinib were 7.84% (2 μM) and 12.25% (4 μM). The proportions of KYSE450TR apoptotic cells treated with 3l were 32.12% (5 μM) and 83.69% (10 μM) (Figure 3D, at 10 μM, Control Vs. 3l, n = 3, F = 2.560, t = −34.311, *p* = 0.000). The proportions of KYSE450TR apoptotic cells treated with icotinib were 8.51% (5 μM) and 10.43% (10 μM).

### EGFR inhibition study

Compounds 3b, 3d, 3e and 3l were also tested for their *in vitro* inhibitory activities against ligand-induced EGFR tyrosine phosphorylation with enzyme-linked immunosorbent assay (ELISA) using icotinib as the positive control. The results are presented as IC_50_ values and are shown in Table 5.

**TABLE 5:** EGFR inhibitory activities of compounds 3b, 3d, 3e and 3l.

Compd no.	R^1^	R^2^	R^3^	R^4^	IC_50_ (μM)		
					EGFR		
					1 h	2 h	
3b	H	H	Cl	H	3.26		71.74
3d	OCH_3_	H	H	H	5.14		25.97
3e	Cl	H	H	H	1.29		4.41
3l	H	H	CF_3_	H	0.42		1.67
icotinib	—	—	—	—	0.00138		0.038

Kinase inhibitory activities of compounds were evaluated using the enzyme-linked immunosorbent assay (ELISA).

### Icotinib-1,2,3-triazole derivatives suppress cancer cell proliferation through the EGFR-TK pathway

**FIGURE 4:**
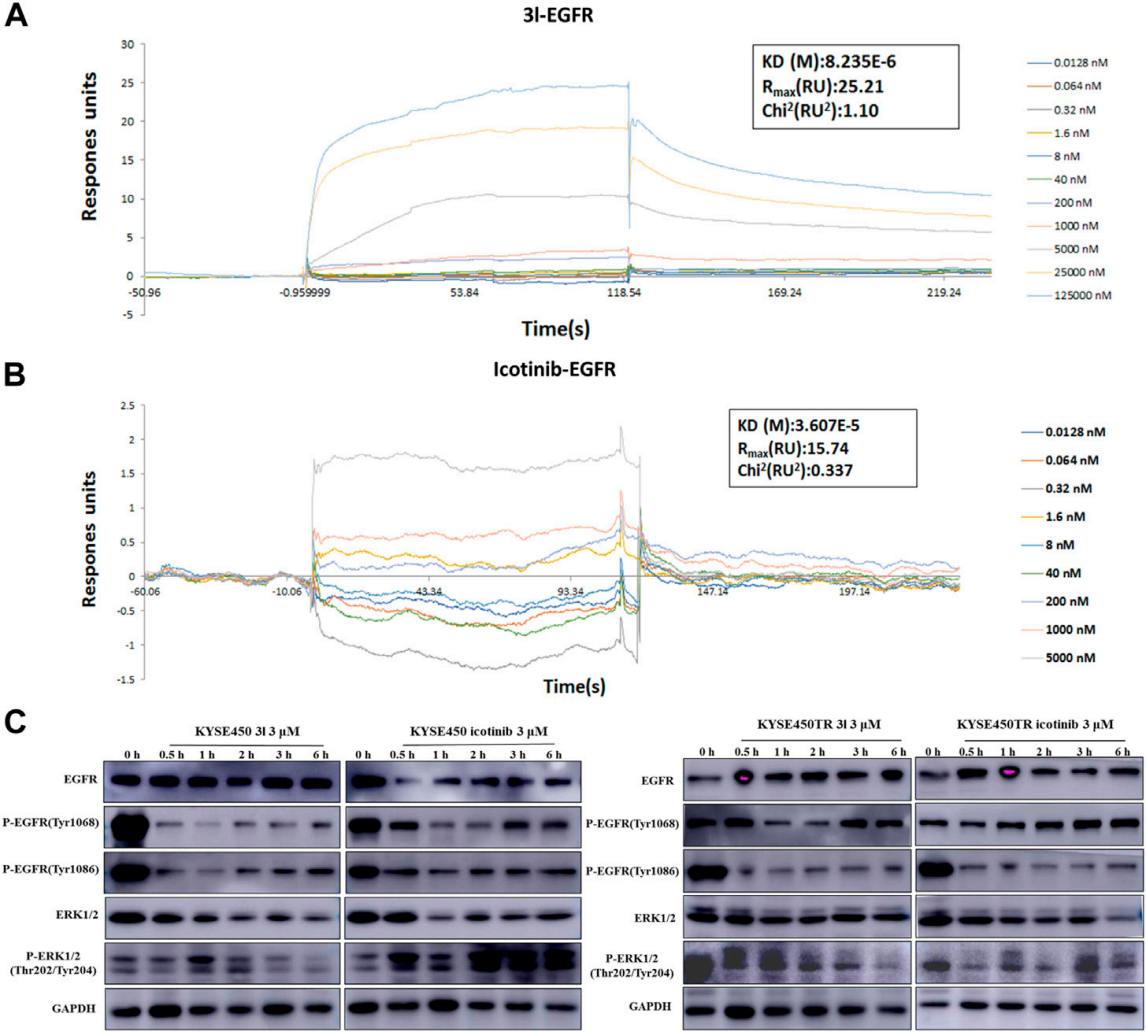
Regulation of cancer cells proliferation due to the binding of 3l with wild-type EGFR. **(A)** Binding sensorgrams for 3l interaction with immobilized wild-type of EGFR. The *K*_D_(M) value between 3l and wild-type EGFR is 8.23 × 10^–6^ **(B)** Binding sensorgrams for icotinib interaction with immobilized wild-type of EGFR. The *K*_D_(M) value between icotinib and wild-type EGFR is 3.60 × 10^–5^ **(C)** The influence of icotinib and 3l on the EGFR-RAS-Raf-MAPK pathway in KYSE450 and KYSE450TR cells.

To further investigate whether the action of 3l in suppressing cancer cell proliferation was related to the EGFR-TK pathway, surface plasmon resonance (SPR) experiments were carried out to study the interaction of 3l and icotinibwith wild-type EGFR proteins. The results are presented in Figures 4A, B. Preliminary results suggested that 3l can bind to EGFR wild protein (H672-A1210) with a *K*_D_ (M) value of 8.23 × 10^–6^, suggesting a strong interaction between 3l and H672-A1210. The *K*_D_ (M) value of icotinib is 3.607 × 10^–5^, the binding force between 3l and wild-type EGFR was significantly better than that of icotinib, and the result was consistent with the result of molecular docking.

Protein electrophoresis was carried out to measure the phosphorylated protein levels of EGFR-TK related marker proteins. The results are presented in Figure 4C. KYSE450/KYSE450TR cells were treated with DMSO or icotinib and 3l for 0.5 h,1 h,2 h,3 h and 6 h, respectively. Analysis of total cell proteins showed that in KYSE450 cells, the P-EGFR (Tyr1068) protein levels of 3l (3 μM) group were lower than the control at 1 h after administration. P-EGFR (Tyr1086), ERK1/2 and P-ERK1/2 (Thr202/Tyr204) protein levels of 3l group were lower than the control at 0.5, 1, 2, 3, and 6 h after administration. There is no obvious difference in EGFR (Tyr1068) protein levels between icotinib and 3l groups. In KYSE450TR cells, the P-EGFR (Tyr1068) protein levels in 3l (3 μM) group were lower than the control at 1 and 2 h after administration. P-ERK1/2 (Thr202/Tyr204) protein levels of 3l group were lower than the control at 6 h after administration. All these results suggest that compounds 3l can regulate cancer cell proliferation through combination with EGFR protein, and regulate the EGFR-TK pathway.

### Compound 3l inhibits tumor growth of cell-line-derived xenografts in ESCC

**FIGURE 5:**
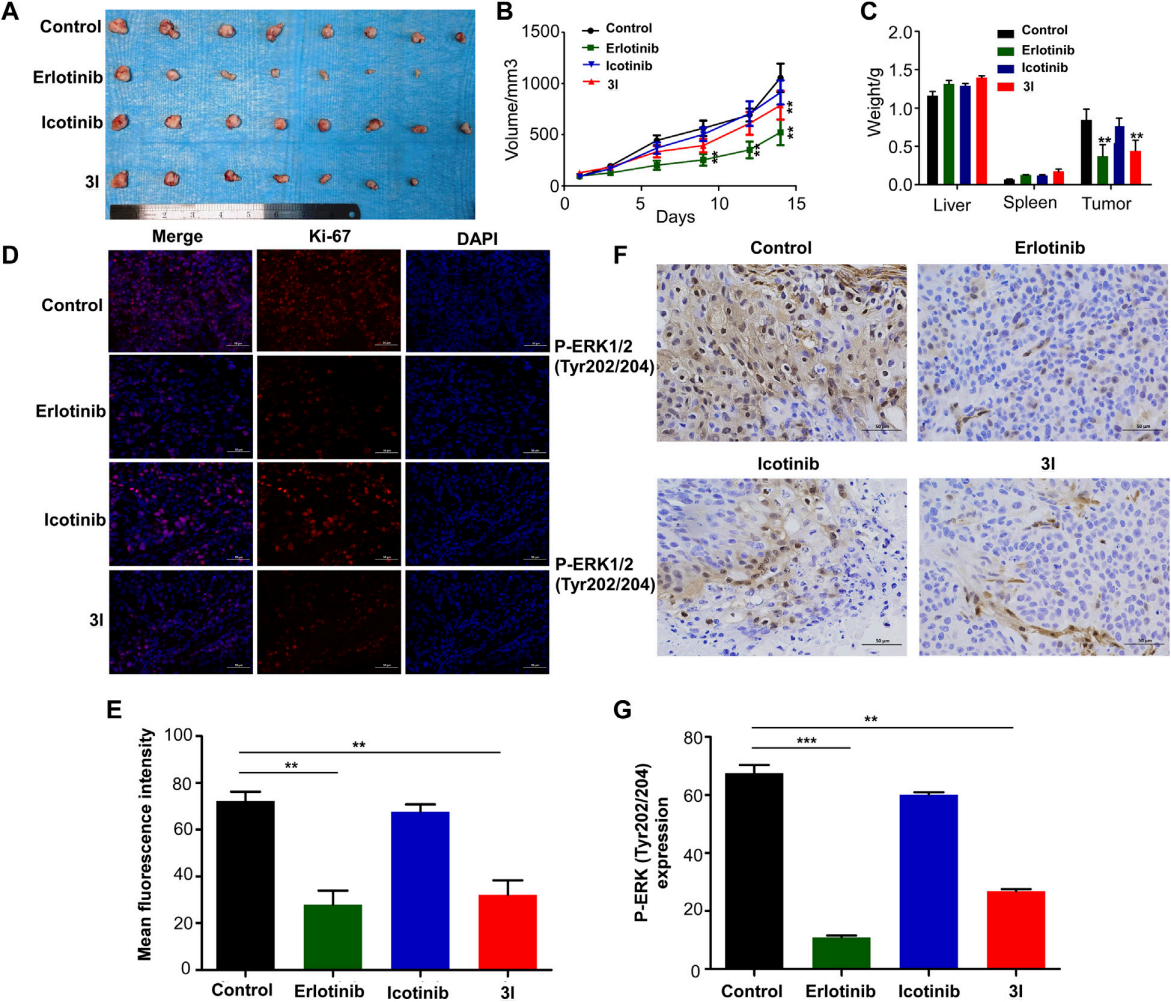
Compound 3l inhibits tumor growth of cell-line-derived xenografts in ESCC. **(A)** Tumor images from the indicated groups were shown **(B)** Tumor sizes were monitored every 3 days for more than two continuous weeks (14th days, Control VS. 3l, n = 8,F = 0.857, t = 5.670, *p* = 0.004) **(C)** Tumor weight, liver weight and spleen weight were measured at the end of the experiment (Tumor weight, Control VS. 3l, n = 8, F = 0.033, t = 8.386, *p* = 0.003) **(D)** Ki-67 expression in harvested xenograft tissues were assessed by immunofluorescence. Representative photographs in different groups were shown **(E)** The statistical analysis for the immunofluorescence images was shown (Control VS. 3l, n = 8, F = 0.210, t = 10.224, *p* = 0.002) **(F)** P-ERK (Tyr202/204) expression in harvested xenograft tissues were assessed by immunohistochemistry. Representative photographs in different groups were shown **(G)** The statistical analysis for the immunohistochemistry images was shown (Control VS. 3l, n = 8, F = 0.09, t = 11.285, *p* = 0.003). Unpaired Student’s t test was used in **(B,C,E and G)**. *p < 0.05, **p < 0.01, ***p < 0.001. Error bars represent the mean ± SD.

To further determine the effect of 3l on ESCC progression *in vivo*, a cell-line-derived xenograft (CDX) model was used. The cell line we used is ESCC cell KYSE450. Erlotinib and icotinib were used as positive controls. Tumors were excised from nude mice at the end of the experiment. Ki-67 and P-ERK (Tyr202/204) expressions in tissues were measured. The results of CDX showed that 3l and erlotinib markedly reduced ESCC tumor volume and average tumor weight compared with those of the control group and icotinib group (Figures 5A–C, Supplementary Figure S24). Next, to further check whether the antitumor effect was associated with its inhibition of EGFR signaling, tumor extracted from each group were prepared and analyzed for the expression levels of Ki-67 and P-ERK (Tyr202/204). Immunohistochemistry and immunofluorescence analysis results showed that the expression of P-ERK (Tyr202/204) and proliferation marker Ki-67 decreased significantly in 3l group and erlotinib group (Figures 5D–G). Overall, these data provide strong evidence that 3l suppresses CDX tumor growth in ESCC *in vivo*, the effect was associated with its inhibition of EGFR signaling.

## Discussion

Gefitinib and icotinib are the first-generation EGFR inhibitor, after about 9–14 months of treatment, the drug resistance problem gradually emerged, and almost all tumors entered the stage of progression again. Therefore, how to solve the problem of icotinib resistance has always been the focus of pharmaceutical researchers (Naruse et al., 2002). The structure of 1,2, 3-triazoles is a very important class of nitrogen-containing heterocyclic compounds, consisting of three nitrogen atoms and two carbon atoms to construct a five-membered heterocyclic ring (Mao et al., 2020b), the molecular formula is C2N3H3. 1, 2, 3-triazoles nitrogen has a special plane rigid structure, make its have strong ability of the DNA embedded, have large dipole moment at the same time, to be able to form hydrophobic, hydrogen bond, van der Waals force, dipole-dipole keys and other non covalent interaction with different biological targets. In addition, the structure features of 1,2, 3-triazoles nitrogen to allow its as amide, esters, carboxylic acid, as electronic equivalent substituents of rigid analogues of olefin, they have a broad spectrum of biological activity, such as antibacterial, antimalarial, antifungal, antiviral, anti-tuberculosis and anticancer active compounds (Mao et al., 2020b).

In this study, we introduced triazolium structures with different substituents into icotinib structure, and obtained 20 compounds. After anti-tumor activity test, it was found that compound 3l, which was obtained by introducing trifluoromethyl with the strongest electron absorption ability, had inhibitory effect on different kinds of tumor cell. In the field of pharmaceutical chemistry, trifluoromethyl is a kind of important chemical group, because of its high lipid solubility, good stability of metabolism, high electron activity and bioavailability, all of these make it as a biologically active molecules and has been widely used. At present, there are nearly hundred kinds of drugs containing trifluoromethyl in structureon the market, such as celecoxib, aptivus, Trifluoperazine Hydrochloride and so on. We first identified icotinib-triazole derivatives 3l, which has inhibitory activity against proliferation of NSCLC and ESCC drug-resistant cells. Preliminary results suggested that EGFR wild-type lung cancer cell H1650 and EGFR mutant lung cancer H1975 cells showed poor sensitivity to icotinib with IC_50_ values of >50 μM, and 3l exhibited stronger killing effects on the lung cancer cell lines than icotinib did. These results are in good agreement with our previous report that compounds 3l exhibited stronger killing effects on the lung EGFR wild-type cancer cell and EGFR mutant ones than icotinib did (Mao et al., 2020b). This study further confirms the important role of trifluoromethyl structure in drug development.

Literature reports show that the expression of EGFR is generally high in esophageal adenocarcinoma or squamous cell carcinoma (Wang et al., 2007; Lyu et al., 2014). The high expression of EGFR is closely related to the proliferation, invasion and poor prognosis of esophageal cancer (EC) as well as the chemoradiotherapy tolerance in EC treatment (Hanawa et al., 2006; Baras et al., 2009). So we also tested the proliferation inhibition activity of 3l on ESCC cells. Results in Table 4 suggested that KYSE70 and KYSE410 cells were less sensitive to icotinib, with IC_50_ values of over 50 μM. KYSE450 cell had an IC_50_ of 30.4 μM and the SHEE cell had an IC_50_ of 19.16 μM. These preliminary results suggested that icotinib had an inhibitory effect on normal esophageal cells before it reached the effective inhibitory dose on ESCC cells. Compound 3l gave the most promising result among the compounds tested, and the IC_50_ values of 3l on these ESCC cell lines were 4.25 μM (KYSE70), 5.94 μM (KYSE410) and 3.4 μM (KYSE450) respectively. Especially, the IC_50_ values of 3l against the drug resistant ESCC cell lines were 5.13 μM (KYSE70TR), 5.3 μM (KYSE410TR) and 8.59 μM (KYSE450TR) respectively (Table 2, data in parentheses). These preliminary results suggested that 3l was active for both EGFR wild-type and taxol-resistant ESCC cells. The IC_50_ of 3l on SHEE cell was 4.82 μM. This value is equivalent to the IC_50_ of 3l against ESCC cell lines, suggesting that further structure optimization is necessary to maintain or to improve the cytotoxicity of the compound against cancer cells and to reduce the cytotoxicity of the compound against normal cells.

We also tested the effect of 3l on apoptosis and cycle of ESCC cells. The results suggested that 3l considerably promoted the apoptosis of tumor cell line KYSE450 and their drug resistant ones in a concentration-dependent manner. In the presence of 3l, the cycle changes of drug-resistant cells were significantly different from control cells, but the decrease of proportion of S stage cells was similar.

Icotinib are the first-generation EGFR inhibitor, EGFR inhibition also be studied. At first, EGFR was allowed to react with the tested compounds for 1 h. The IC_50_ value of icotinib was 0.00138 μM. The icotinib derivatives showed slightly lower EGFR tyrosine phosphorylation inhibitory potency (IC_50_ values ranging from 0.42 μM to 5.14 μM). The preliminary data showed that the IC_50_ value of icotinib changed from 0.00138 to 0.038 μM, while the IC_50_ value for 3l was 0.42 μM at the first hour with slight increase at the second hour (IC_50_ value 1.67 μM). Enzyme activity experiment *in vitro* suggested that compound 3l showed more stable inhibition of EGFR activity, and may lead to betterEGFR-TK pathway inhibition with prolonged time of action. Study of the reason of this difference is in progress.

One of the major signaling pathways for EGFR activation is the RAS-Raf-MAPK pathway. The crude frame-mode pathway of the EGFR-mediated mitogen-activated protein kinase (MAPK) pathway is: EGFR ligand (EGF, TGF-α, *etc.*)-EGFR-adaptor protein (Grb2,*etc.*)-SOS (Ras-specific nucleotide exchange factor)-RAS-Raf (MAPK kinase)-MEK (MAPK kinase)-ERK-gene transcription that encodes nuclear transcription factors. For the study, the expression changes of EGFR, P-EGFR, ERK1/2 and P-ERK1/2, the related molecules in the EGFR-TK signal transduction pathway were measured to investigate the binding and regulatory relationship between 3l and EGFR in cancer cells (Chen et al., 2019). The result suggested that 3l combined with EGFR, down-regulated EGFR phosphorylation level, and regulated the proliferation of cancer cells through the EGFR-RAS-Raf-MAPK pathway. The result of western blot is similar to that of the *in vitro* enzyme activity experiment. When the cells were treated with 3l for 1 h, the inhibition effect of 3l on P-EGFR (Tyr1068 of KYSE450, Tyr1086 of KYSE450TR) expression was not better than that of icotinib. However, with the prolongation of time, the inhibition of 3l on P-EGFR and P-ERK expression was gradually superior to that of icotinib with different specific time for each cell line. These results suggest that the inhibition effect of 3l on EGFR-TK pathway is time-dependent.

To further determine the effect of 3l on ESCC progression *in vivo*, a cell-line-derived xenograft (CDX) model was used. The results of CDX showed that 3l and erlotinib markedly reduced ESCC tumor volume and average tumor weight compared with those of the control groups (Figures 5A–C). Next, to further check whether the antitumor effect was associated with its anti-proliferation and inhibition of EGFR signaling, tumor extracted from each group were prepared and analyzed for the expression levels of Ki-67 and P-ERK (Tyr202/204). Immunohistochemistry and immunofluorescence analysis results showed that the expression of P-ERK (Tyr202/204) and proliferation marker Ki-67 decreased significantly in 3l group and erlotinib group (Figures 5D–G). Overall, these data provide strong evidence that 3l suppresses CDX tumor growth in ESCC *in vivo*, the effect was associated with its inhibition of EGFR signaling.

**FIGURE 6:**
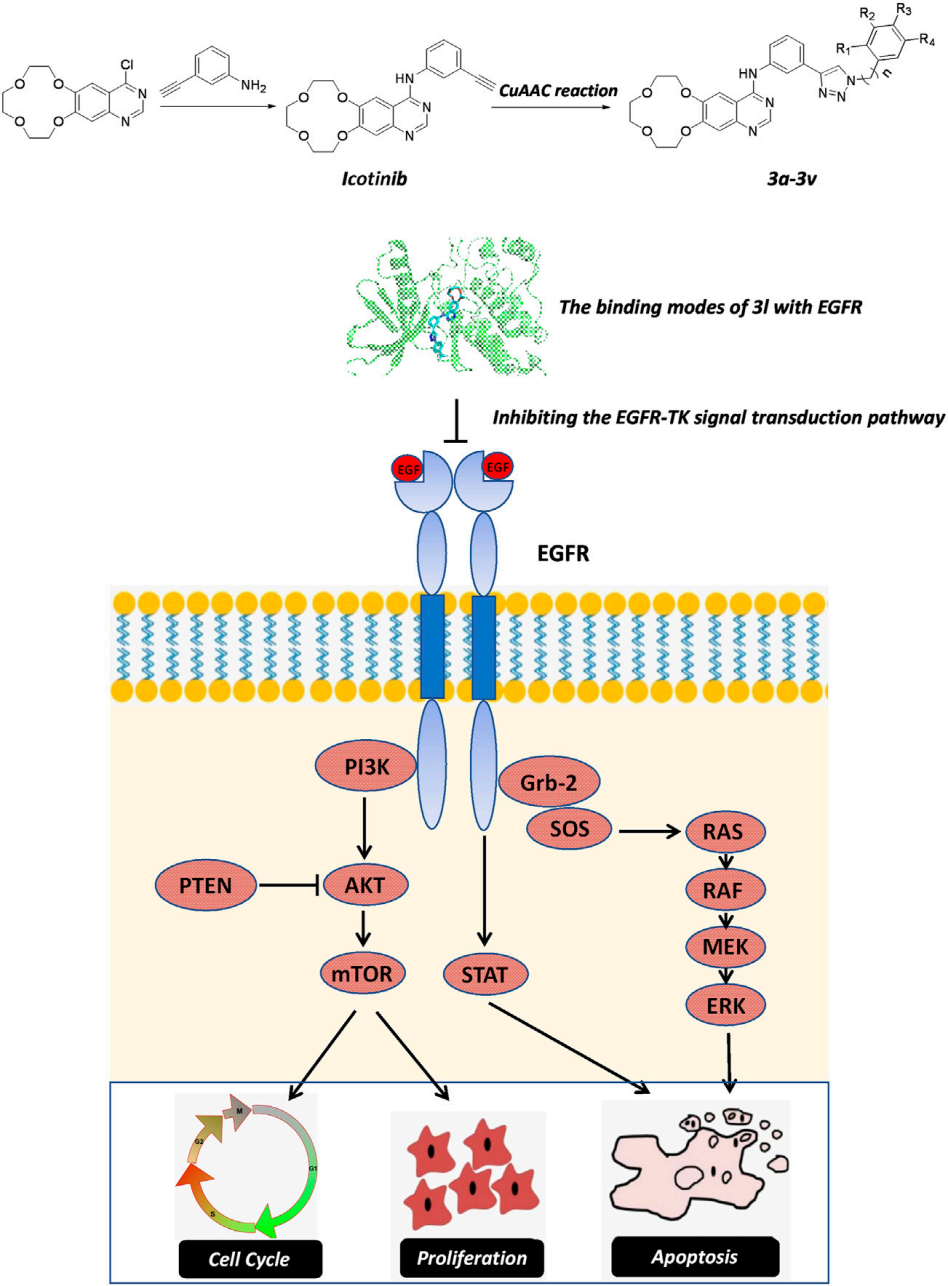
The proposed action mechanism oficotinib-triazole derivativesin ESCC cells. Icotinib-triazole derivatives inhibits cell proliferation, induces apoptosis and cell cycle arrest of ESCC cells *via* EGFR-mediated mitogen-activated protein kinase (MAPK) pathway, leading to the inactivation of RAS-Raf-MAPK pathway.

In summary, icotinib derivatives containing 1,2,3-triazole ring were active against three ESCC cell lines and their drug resistant cells. Several of these compounds exhibited remarkable antitumor activity than icotinib did. Among them, compound 3l demonstrated promising anti-proliferative effects and cytotoxicity against all the cell lines. The underlying mechanisms of 3l induced cancer cell death were explored *via* inducing apoptosis and cell cycle arrest. 3l can inhibit ESCC proliferation by interacting with EGFR protein, and regulate the EGFR-TK pathway (Figure 6). *In vivo* study also showed that 3l could inhibit tumor growth of cell-line-derived xenografts in ESCC. Combining these results, compound 3l represents a new type of lead compound for the study of new anticancer drugs, and future lead optimization study will focus on improving the anticancer activity, the pharmacokinetic property of the compounds and to improve the selectivity of the compounds against cancer cells.

## Data Availability

The original contributions presented in the study are included in the article/Supplementary Materials, further inquiries can be directed to the corresponding authors.

## References

[B1] Al-Quteimat O. M. Amer A. M. (2020). A review of Osimertinib in NSCLC and pharmacist role in NSCLC patient care. J. Oncol. Pharm. Pract. 26, 1452–1460. 10.1177/1078155220930285 32525442

[B2] Asokan S. Sridhar P. Qureshi M. M. Bhatt M. Truong M. T. Suzuki K. (2020). Presentation, treatment, and outcomes of vulnerable populations with esophageal cancer treated at a safety-net hospital. Semin. Thorac. Cardiovasc. Surg. 32, 347–354. doi:. 10.1053/j.semtcvs.2019.12.008 31866573

[B3] Baras A. Yu Y. Filtz M. Kim B. Moskaluk C. A. (2009). Combined genomic and gene expression microarray profiling identifies ECOP as an upregulated gene in squamous cell carcinomas independent of DNA amplification. Oncogene 28, 2919–2924. 10.1038/onc.2009.150 19525979

[B4] Chen X. Wu H. Chen H. Wang Q. Xie X. J. Shen J. (2019). Astragaloside VI promotes neural stem cell proliferation and enhances neurological function recovery in transient cerebral ischemic injury via activating EGFR/MAPK signaling cascades. Mol. Neurobiol. 56, 3053–3067. 10.1007/s12035-018-1294-3 30088176

[B5] Eskilsson E. Røsland G. V. Solecki G. Wang Q. Harter P. N. Graziani G. (2018). EGFR heterogeneity and implications for therapeutic intervention in glioblastoma. Neuro. Oncol. 20, 743–752. 10.1093/neuonc/nox191 29040782 PMC5961011

[B6] Hanawa M. Suzuki S. Dobashi Y. Yamane T. Kono K. Enomoto N. (2006). EGFR protein overexpression and gene amplification in squamous cell carcinomas of the esophagus. Int. J. Cancer 118, 1173–1180. 10.1002/ijc.21454 16161046

[B7] Huang J. Fan Q. Lu P. Ying J. Ma C. Liu W. (2016). Icotinib in patients with pretreated advanced esophageal squamous cell carcinoma with EGFR overexpression or EGFR gene amplification: A single-arm, multicenter phase 2 study. J. Thorac. Oncol. 11, 910–917. 10.1016/j.jtho.2016.02.020 26980473

[B8] Jia Y. Yun C. H. Park E. Ercan D. Manuia M. Juarez J. (2016). Overcoming EGFR(T790M) and EGFR(C797S) resistance with mutant-selective allosteric inhibitors. Nature 534, 129–132. 10.1038/nature17960 27251290 PMC4929832

[B9] Kros J. M. Huizer K. Hernández-Laín A. Marucci G. Michotte A. Pollo B. (2015). Evidence-based diagnostic algorithm for glioma: Analysis of the results of Pathology panel review and molecular parameters of EORTC 26951 and 26882 trials. J. Clin. Oncol. 33, 1943–1950. 10.1200/jco.2014.59.0166 25918297

[B10] Lee D. H. (2017). Treatments for EGFR-mutant non-small cell lung cancer (NSCLC): The road to a success, paved with failures. Pharmacol. Ther. 174, 1–21. 10.1016/j.pharmthera.2017.02.001 28167215

[B11] Liu Z. Chen Z. Wang J. Zhang M. Li Z. Wang S. (2018). Mouse avatar models of esophageal squamous cell carcinoma proved the potential for EGFR-TKI afatinib and uncovered Src family kinases involved in acquired resistance. J. Hematol. Oncol. 11, 109. 10.1186/s13045-018-0651-z 30157900 PMC6114252

[B12] Loibl S. Gianni L. (2017). HER2-positive breast cancer. Lancet (London, Engl. 389, 2415–2429. 10.1016/s0140-6736(16)32417-5 27939064

[B13] Lyu X. Huang J. Liu J. Wang W. Su Y. Zhang W. (2014). Detection and significance of epidermal growth factor receptor mutation in esophageal, esophagogastric junction and gastric cancers. Zhonghua Zhong Liu Za Zhi 36, 346–350. 25030589

[B14] Mao L. Sun G. Zhao J. Xu G. Yuan M. Li Y. M. (2020). Design, synthesis and antitumor activity of icotinib derivatives. Bioorg. Chem. 105, 104421. 10.1016/j.bioorg.2020.104421 33181408

[B15] Mao Y. S. Gao S. G. Wang Q. Shi X. T. Li Y. Gao W. W. (2020). Epidemiological characteristic and current status of surgical treatment for esophageal cancer by analysis of national registry database. Zhonghua Zhong Liu Za Zhi 42, 228–233. 10.3760/cma.j.cn112152-20191112-00729 32252202

[B16] Meluch A. A. Bendell J. C. Peyton J. D. Rudolph P. Rubin M. S. Webb C. D. (2010). A phase II trial of preoperative chemoradiation therapy plus bevacizumab and erlotinib in the treatment of localized esophageal cancer. J. Clin. Oncol. 28, 4108. 10.1200/jco.2010.28.15_suppl.4108 20697089

[B17] Morris G. M. Goodsell D. S. Halliday R. S. Huey R. Hart W. E. Belew R. K. Automated docking using a Lamarckian genetic algorithm and an empirical binding free energy function. J. Comput. Chem. 19 (1998) 1639–1662. 10.1002/(sici)1096-987x(19981115)19:14<1639::aid-jcc10>3.0.co;2-b

[B18] Morris G. M. Huey R. Lindstrom W. Sanner M. F. Belew R. K. Goodsell D. S. (2009). AutoDock4 and AutoDockTools4: Automated docking with selective receptor flexibility. J. Comput. Chem. 30, 2785–2791. 10.1002/jcc.21256 19399780 PMC2760638

[B19] Naruse I. Ohmori T. Ao Y. Fukumoto H. Kuroki T. Mori M. (2002). Antitumor activity of the selective epidermal growth factor receptor-tyrosine kinase inhibitor (EGFR-TKI) Iressa (ZD1839) in an EGFR-expressing multidrug-resistant cell line *in vitro* and *in vivo* . Int. J. Cancer 98, 310–315. 10.1002/ijc.10173 11857424

[B20] Ren W. Sha H. Yan J. Wu P. Yang J. Li R. (2018). Enhancement of radiotherapeutic efficacy for esophageal cancer by paclitaxel-loaded red blood cell membrane nanoparticles modified by the recombinant protein anti-EGFR-iRGD. J. Biomater. Appl. 33, 707–724. 10.1177/0885328218809019 30388386

[B21] Rizvi S. M. Shakil S. Haneef M. (2013). A simple click by click protocol to perform docking: AutoDock 4.2 made easy for non-bioinformaticians. Excli. J. 12, 831–857. 26648810 PMC4669947

[B22] Russo A. Lopes A. R. McCusker M. G. Garrigues S. G. Ricciardi G. R. Arensmeyer K. E. (2020). New targets in lung cancer (excluding EGFR, ALK, ROS1). Curr. Oncol. Rep. 22, 48. 10.1007/s11912-020-00909-8 32296961

[B23] Sutter A. P. Höpfner M. Huether A. Maaser K. Scherübl H. (2010). Targeting the epidermal growth factor receptor by erlotinib (Tarceva) for the treatment of esophageal cancer. Int. J. Cancer 118, 1814–1822. 10.1002/ijc.21512 16217753

[B24] Then E. O. Lopez M. Saleem S. Gayam V. Sunkara T. Culliford A. (2020). Esophageal cancer: An updated surveillance epidemiology and end results database analysis. World J. Oncol. 11, 55–64. 10.14740/wjon1254 32284773 PMC7141161

[B25] Von Hoff D. D. Casper J. Bradley E. Trent J. M. Hodach A. Reichert C. (1980). Direct cloning of human neuroblastoma cells in soft agar culture. Cancer Res. 40, 3591–3597. 7002289

[B26] Wang K. L. Wu T. T. Choi I. S. Wang H. Resetkova E. Correa A. M. (2007). Expression of epidermal growth factor receptor in esophageal and esophagogastric junction adenocarcinomas: Association with poor outcome. Cancer 109, 658–667. 10.1002/cncr.22445 17211865

[B27] Xu Y. Xie Z. Lu H. (2018). Detection of epidermal growth factor receptor mutation in the peripheral blood of patients with esophageal carcinoma to guide epidermal growth factor receptor-tyrosine kinase inhibitor treatment. J. Cancer Res. Ther. 14, 103–105. 10.4103/jcrt.JCRT_735_17 29516969

[B28] Zhang L. Ma J. Han Y. Liu J. Zhou W. Hong L. (2016). Targeted therapy in esophageal cancer. Expert Rev. Gastroenterol. Hepatol. 10, 595–604. 10.1586/17474124.2016.1140036 26895097

