# Supplementary material for: Icotinib derivatives as tyrosine kinase inhibitors with anti-esophageal squamous carcinoma activity
Source: Front Pharmacol. 2022 Nov 16;13:1028692. doi: 10.3389/fphar.2022.1028692 (PMC9709406; doi:10.3389/fphar.2022.1028692)
Supplement: Supplementary file 1 [file Presentation1.pdf]

Supporting Information

Icotinib Derivatives as Tyrosine Kinase inhibitors with anti-Esophageal Squamous Carcinoma Activity

Table of content:	Page #
1. Chemistry:<br>1.1. Materials and instrumentation	S1
1.2. Experimental protocols for the preparation of compounds 3a-3v	S2-S7
2. Supplementary figures (S1-S2231)	S8-S712
3. Biological studies:<br>3.1. Plate clone formation assay (Supplementary figures S23)<br>3.2. Body weight changes in nude mice during drug administration(Supplementary figures S24)	S72

1. Chemistry:

   1.1. Materials and instrumentation.

   All reagents and solvents obtained from commercially available source were used without further treatment. ^{1}H NMR and ^{13}C NMR spectra were acquired in DMSO-d_6 or $CDCl_3$ solution with a Bruker 400 spectrometer at 400.13 and 100.61 MHz, respectively. Chemical shifts (d) were given in parts per million with tetramethylsilane as internal reference and coupling constants were expressed in hertz. High-resolution mass spectra (HR MS) measurements were carried out using an Bruker MicrOTOF-Q II mass spectrometer.

   1.2. Experimental protocols for the preparation of compounds 1-22.

   1.2.1.Preparation of N-(3-ethynylphenyl)-7,8,10,11,13,14-hexahydro-[1,4,7,10] tetraoxacyclododecino [2,3-g] quinazolin-4-amine (icotinib, 6)

Compound 1 (31 g, 0.1 mol) was suspended in 500 mL isopropanol (**Scheme 1.**). To the solution was added 3-aminophenylacetylene. The suspension was stirred at room temperature for 5 min. It was then heated to 85 ℃ and was stirred under nitrogen for 5h. Solid would gradually formed, and the course of the reaction was monitored with TLC. After the completion of the reaction, the reaction mixture was transferred to ice water and the mixture was stirred for half an hour. The solid was collected by filtration and was washed twice with isopropanol (50 mL) to give 27 g of icotinib. 1H NMR (600 MHz, DMSO-d6): 11.68 (s, 1H, NH), 8.87 (s, 1H, CH), 8.84 (s, 1H, Ar-H), 7.93 (s, 1H, Ar-H), 7.84 (d, J=8.1Hz, 1H, Ar-H), 7.49 (dd, J1=10.0 Hz, J2=5.8 Hz, 2H, Ar-H), 7.40 (d, J=7.7Hz, 1H, Ar-H), 4.44-4.32 (m, 4H, CH2CH2), 4.28 (s, 1H, CH), 3.82-3.71 (m, 4H, CH2CH2), 3.60 (dd, J1=9.4 Hz, J2= 5.3 Hz, 4H,

CH_2CH_2); 13C NMR (101 MHz, DMSO-d6): 158.9, 158.7, 151.7, 149.7, 137.8, 136.4, 129.8, 129.6, 127.9, 125.7, 122.4, 112.9, 108.6, 104.6, 83.4, 81.8, 73.6, 71.6, 70.7, 70.4, 68.9, 68.7.

1.2.2.General procedure for the preparation of compound 3a-3v

Aryl-azido (1.2 mmol) and icotinib(1.0 mmol) were added to 15 mL mixed solvent (water/tert-butanol =2:1). Copper sulfate pentahydrate (0.1 mmol) and sodium ascorbate (0.2 mmol) were added and the mixture was stirred at 80 ℃. After the completion of the reaction (monitored by TLC), the mixture was extracted with dichloromethane (15 mL×3). The combined organic phase was washed successively with water and brine, dried over sodium sulfate and concentrated in vacuo. The residue was purified by through column chromatography (CH_2Cl_2/MeOH=20:1) to give the desired compound a as a crystalline powder.

{3-[1-(3-Fluoro-phenyl)-1H-[1,2,3]triazol-4-yl]-phenyl}-(7,8,10,11,13,14-hexahydro-6,9,12,15-tetraoxa-1,3-diaza-cyclododeca[b]naphthalen-4-yl)-amine (3a):

Yellow solid, yield 81%,m.p. 123–126 ℃; 1H NMR (600 MHz, DMSO‑d6): δ 9.66 (s, 1H, CH),9.38 (s, 1H, CH), 8.56 (s, 1H, NH), 8.44 (s, 1H, Ar-H), 8.23 (s, 1H, Ar-H),7.98 (d, J = 7.9 Hz, 1H, Ar-H), 7.91 (dd, J1 = 19.0 Hz, J2 = 9.0 Hz,2H, Ar-H), 7.70 (dd, J1 = 14.8 Hz, J2 = 7.8 Hz, 1H, Ar-H), 7.65 (d,J = 7.5 Hz, 1H, Ar-H), 7.53 (t, J = 7.8 Hz, 1H, Ar-H), 7.39 (t,J = 8.3 Hz, 1H, Ar-H), 7.33 (s, 1H, Ar-H), 4.32 (d, J = 12.4 Hz, 4H,CH_2CH_2), 3.79 (d, J = 19.9 Hz, 4H, CH_2CH_2), 3.65 (s, 4H, CH_2CH_2); 13CNMR (150 MHz, DMSO‑d6):δ 163.8, 162.1, 157.1, 156.5, 153.8, 150.3,147.9, 140.6, 138.4, 132.4, 130.8, 129.7, 122.6, 121.0, 120.3, 119.3,116.4, 115.8, 112.1, 110.7, 108.1, 107.9, 73.4, 70.9, 70.9, 70.5, 69.3,68.9; HR MS(ESI) m/z: calcd for $C_{28}H_{26}O_4N_6F$ [M + H]+ 529.1994,found 529.2000.

{3-[1-(4-Chloro-phenyl)-1H-[1,2,3]triazol-4-yl]-phenyl}-(7,8,10,11,13,14-hexahydro-6,9,12,15-tetraoxa-1,3-diaza-cyclododeca[b]naphthalen-4-yl)-amine (3b):

Brown solid, yield 92%,m.p. 132–135 ℃; 1H NMR (600 MHz, DMSO‑d6): δ 9.84 (s, 1H, CH),9.37 (s, 1H, CH), 8.59 (s, 1H, NH), 8.44 (s, 1H, ArH), 8.30 (s, 1H, Ar-H), 8.04 (d, J = 7.7 Hz, 2H, Ar-H), 7.95 (d,J = 8.0 Hz, 1H, Ar-H), 7.74 (d, J = 7.7 Hz, 2H, Ar-H), 7.67 (d,J = 7.6 Hz, 1H, Ar-H), 7.53 (t, J = 7.8 Hz, 1H, Ar-H), 7.34 (s, 1H,Ar-H), 4.33 (d, J = 13.2 Hz, 4H, CH_2CH_2), 3.79 (d, J = 22.6 Hz, 4H,CH_2CH_2), 3.65 (s, 4H, CH_2CH_2); 13C NMR (150 MHz, DMSO‑d6):δ 157.3,156.7, 150.4, 147.9, 140.4, 135.9, 133.6, 130.8, 130.4, 129.6, 126.5,125.7, 122.7, 122.1, 121.2, 119.5, 110.9, 73.4, 70.9, 70.5, 69.2, 68.9,64.3, 45.9, 8.9; HR MS(ESI) m/z: calcd for $C_{28}H_{26}O_4N_6Cl$ [M + H]+545.1699, found 545.1703.

{3-[1-(4-Fluoro-phenyl)-1H-[1,2,3]triazol-4-yl]-phenyl}-(7,8,10,11,13,14-hexahydro-6,9,12,15-tetraoxa-1,3-diaza-cyclododeca[b]naphthalen-4-yl)-amine (3c):

White solid, yield 74%,m.p. 129–132 ℃; 1H NMR (600 MHz, DMSO‑d6): δ 9.70 (s, 1H, CH),9.31 (s, 1H, CH), 8.62 (s, 1H, NH), 8.43 (s, 1H, Ar-H), 8.25 (s, 1H, Ar-H),8.04 (d, J1 = 8.4 Hz, J2 = 4.7 Hz, 2H, Ar-H), 7.96 (d, J = 7.9 Hz, 1H,Ar-H),

7.66 (d, J = 7.5 Hz, 1H, Ar-H), 7.63–7.39 (m, 3H, Ar-H), 7.35 (s,1H, Ar-H), 4.32 (s, 4H, CH2CH2), 3.80 (d, J = 21.6 Hz, 4H, CH2CH2),3.65 (s, 4H, CH2CH2); 13C NMR (150 MHz, DMSO‑d6):δ 162.9, 161.4,157.2, 156.5, 150.3, 147.8, 140.5, 133.7, 130.9, 129.6, 122.9, 122.8,122.6, 121.1, 120.5, 119.4, 117.4, 117.2, 111.9, 110.8, 73.4, 70.9, 70.9,70.5, 69.3, 68.9; HR MS (ESI) m/z: calcd for C28H26O4N6F [M + H]+529.1994, found 529.2000.

(7,8,10,11,13,14-Hexahydro-6,9,12,15-tetraoxa-1,3-diaza-cyclododeca[b]naphthalen-4-yl)-{3-[1-(2-methoxy-phenyl)-1H-[1,2,3]triazol-4-yl]-phenyl}-amine (3d):

White solid, yield 82%, m.p. 121–124 ℃;1H NMR (600 MHz, DMSO‑d6): δ 9.63 (s, 1H, CH), 8.92 (s, 1H, CH), 8.53(s, 1H, NH), 8.41 (s, 1H, Ar-H), 8.25 (s, 1H, Ar-H), 7.97 (d, J = 8.0 Hz,1H, Ar-H), 7.70 (d, J = 7.8 Hz, 1H, Ar-H), 7.66 (d, J = 7.6 Hz, 1H,Ar-H), 7.57 (t, J = 7.9 Hz, 1H, Ar-H), 7.50 (t, J = 7.8 Hz, 1H, Ar-H),7.36 (d, J = 8.4 Hz, 1H, Ar-H), 7.32 (s, 1H, Ar-H), 7.19 (t, J = 7.6 Hz,1H, Ar-H), 4.32 (d, J = 13.1 Hz, 4H, CH2CH2), 3.90 (s, 3H, OCH3),3.80 (d, J = 20.7 Hz, 4H, CH2CH2), 3.65 (s, 4H, CH2CH2); 13C NMR(150 MHz, DMSO‑d6): δ 157.1, 156.4, 153.9, 152.3, 150.2, 148.1, 146.7,140.6, 131.4, 131.2, 129.5, 126.4, 126.2, 123.9, 122.3, 121.4, 121.0,119.2, 113.5, 112.1, 110.8, 110.3, 73.4, 70.9, 70.8, 70.5, 69.3, 68.9;HR MS (ESI) m/z: calcd for C29H29O5N6 [M + H]+ 541.2194, found541.2197.

{3-[1-(2-Chloro-phenyl)-1H-[1,2,3]triazol-4-yl]-phenyl}-(7,8,10,11,13,14-hexahydro-6,9,12,15-tetraoxa-1,3-diaza-cyclododeca[b]naphthalen-4-yl)-amine (3e):

Yellow solid, yield 94%,m.p. 125–127 ℃; 1H NMR (600 MHz, DMSO‑d6): δ 9.63 (s, 1H, CH),9.07 (s, 1H, CH), 8.53 (s, 1H, NH), 8.45 (s, 1H, Ar-H), 8.22 (s, 1H,Ar-H), 7.97 (d, J = 8.1 Hz, 1H, Ar-H), 7.82 (t, J = 9.0 Hz, 2H, Ar-H),7.69–7.63 (m, 3H, Ar-H), 7.51 (t, J = 7.9 Hz, 1H, Ar-H), 7.32 (s, 1H,Ar-H), 4.31 (d, J = 14.9 Hz, 4H, CH2CH2), 3.80 (d, J = 21.4 Hz, 4H,CH2CH2), 3.65 (s, 4H, CH2CH2); 13C NMR (150 MHz, DMSO‑d6): δ157.1, 156.5, 153.9, 150.2, 148.2, 147.0, 140.6, 131.1, 130.9, 129.6,129.1, 129.0, 128.9, 124.2, 119.3, 112.2, 110.7, 110.2, 73.4, 70.9, 70.8,70.5, 69.3, 68.9; HR MS (ESI) m/z: calcd for C28H26O4N6Cl [M + H]+545.1699, found 545.1704.

(7,8,10,11,13,14-Hexahydro-6,9,12,15-tetraoxa-1,3-diaza-cyclododeca[b]naphthalen-4-yl)-[3-(1-phenyl-1H-[1,2,3]triazol-4-yl)-phenyl]-amine (3f):

Yellow solid, yield 87%, m.p. 148–150 ℃; 1H NMR (600 MHz, DMSO‑d6): δ9.68 (s, 1H, CH), 9.33 (s, 1H, CH), 8.53 (s, 1H, NH), 8.45 (s, 1H, Ar-H),8.24 (s, 1H, Ar-H), 7.99 (t, J = 10.1 Hz, 3H, Ar-H), 7.67–7.64 (m,3H, Ar-H), 7.53 (q, J = 7.5 Hz, 2H, Ar-H), 7.32 (s, 1H, Ar-H), 4.32(d, J = 13.6 Hz, 4H, CH2CH2), 3.79 (d, J = 20.2 Hz, 4H, CH2CH2),3.65 (s, 4H, CH2CH2); 13C NMR (150 MHz, DMSO‑d6): δ 157.2, 156.4,153.9, 150.2, 148.1, 147.8, 140.6137.1, 131.0, 130.4, 129.6, 129.2, 122.5, 121.0, 120.5, 120.2, 119.3,112.2, 110.7, 110.3, 73.4, 70.9, 70.9, 70.5, 69.3, 68.9; HR MS (ESI) m/z:calcd for C28H26O4NaN6 [M + Na]+ 533.1913, found 533.1915.

{3-[1-(3-Chloro-phenyl)-1H-[1,2,3]triazol-4-yl]-phenyl}-(7,8,10,11,13,14-hexahydro-6,9,12,15-tetraoxa-1,3-diaza-cyclododeca[b]naphthalen-4-yl)-amine (3g):

Yellow solid, yield 79%,m.p. 116–119 ℃; 1H NMR (600 MHz, DMSO‑d6): δ 9.70 (s, 1H, CH),9.41 (s, 1H, CH), 8.68 (s, 1H, NH), 8.44 (s, 1H, Ar-H), 8.28 (s, 1H, Ar-H),8.13 (s, 1H, Ar-H), 7.99 (dd, J1 = 27.4 Hz, J2 = 8.0 Hz, 2H, Ar-H), 7.67(dd, J1 = 16.5 Hz, J2 = 8.0 Hz, 2H, Ar-H), 7.60 (d, J = 8.0 Hz, 1H,Ar-H), 7.53 (t, J = 7.8 Hz, 1H, Ar-H), 7.37 (s, 1H, Ar-H), 4.32 (s, 4H,CH2CH2), 3.79 (d, J = 21.8 Hz, 4H, CH2CH2), 3.65 (s, 4H, CH2CH2); 13CNMR (150 MHz, DMSO‑d6): δ 157.2, 156.5, 153.7, 150.3, 147.9, 140.5,138.2, 134.7, 132.2, 130.8, 130.1, 129.7, 129.0, 122.6, 121.1, 120.4,120.3, 119.4, 119.0, 112.0, 110.8, 93.3, 73.4, 70.9, 70.9, 70.5, 69.3,68.9; HR MS (ESI) m/z: calcd for C28H26O4N6Cl [M + H]+ 545.1699,found 545.1705.

(7,8,10,11,13,14-Hexahydro-6,9,12,15-tetraoxa-1,3-diaza-cyclododeca[b]naphthalen-4-yl)-{3-[1-(2-iodo-phenyl)-1H-[1,2,3]triazol-4-yl]-phenyl}-amine (3h):

White solid, yield 88%, m.p. 137–140 ℃;1H NMR (600 MHz, DMSO‑d6): δ 9.65–9.60 (m, 1H, CH), 8.99 (s, 1H,CH), 8.58–8.46 (m, 2H, Ar-H), 8.32–8.14 (m, 2H, Ar-H), 7.96 (d,J = 10.5 Hz, 1H, Ar-H), 7.68–7.65 (m, 2H, Ar-H), 7.57–7.40 (m, 2H,Ar-H), 7.32 (d, J = 6.1 Hz, 1H, Ar-H), 5.70 (m, 1H, NH), 4.31 (s, 4H,CH2CH2), 3.79 (d, J = 25.0 Hz, 4H, CH2CH2), 3.65 (s, 4H, CH2CH2);13C NMR (150 MHz, DMSO‑d6): δ 157.1, 156.5, 153.9, 150.3, 148.0,147.0, 140.6, 140.3, 140.3, 140.0, 131.1, 130.2, 129.9, 129.6, 129.4,128.6, 124.0, 122.3, 121.0, 119.2, 112.1, 110.8, 110.2, 96.5, 73.4, 70.9,70.8, 70.5, 69.3, 68.9, 58.0; HR MS (ESI) m/z: calcd for C28H26O4N6I[M + H]+ 637.1060, found 637.1065.

(7,8,10,11,13,14-Hexahydro-6,9,12,15-tetraoxa-1,3-diaza-cyclododeca[b]naphthalen-4-yl)-{3-[1-(3-methoxy-phenyl)-1H-[1,2,3]triazol-4-yl]-phenyl}-amine (3i):

Brown solid, yield 72%, m.p. 122–125 ℃;1H NMR (600 MHz, DMSO‑d6): δ 9.69 (s, 1H, CH), 9.39 (s, 1H, CH), 8.58(s, 1H, NH), 8.47 (s, 1H, Ar-H), 8.27 (s, 1H, Ar-H), 8.02 (d, J = 7.9 Hz,1H, Ar-H), 7.70 (d, J = 7.6 Hz, 1H, Ar-H), 7.63–7.56 (m, 4H, Ar-H),7.37 (s, 1H, Ar-H), 7.14 (d, J = 7.9 Hz, 1H, Ar-H), 4.36 (d, J = 11.7 Hz,4H, CH2CH2), 3.94 (s, 3H, OCH3), 3.84 (d, J = 20.1 Hz, 4H, CH2CH2),3.70 (s, 4H, CH2CH2); 13C NMR (150 MHz, DMSO‑d6): δ 160.7, 157.1,156.5, 153.9, 150.3, 148.1, 147.8, 140.6, 138.2, 131.4, 131.0, 129.6,122.5, 121.0, 120.2, 119.3, 114.9, 112.4, 112.2, 110.7, 110.2, 106.1,73.4, 70.9, 70.9, 70.5, 69.3, 68.9, 56.1; HR MS (ESI) m/z: calcd forC29H28O5N6Na [M + Na]+ 563.2013, found 563.2017.

{3-[1-(4-Bromo-phenyl)-1H-[1,2,3]triazol-4-yl]-phenyl}-(7,8,10,11,13,14-hexahydro-6,9,12,15-tetraoxa-1,3-diaza-cyclododeca[b]naphthalen-4-yl)-amine (3j):

White solid, yield 69%,m.p. 158–161 ℃; 1H NMR (600 MHz, DMSO‑d6): δ 9.63 (s, 1H, CH),9.36 (s, 1H, CH), 8.53 (s, 1H, NH), 8.44 (s, 1H, Ar-H), 8.22 (s, 1H, Ar-H),7.97 (d, J = 8.6 Hz, 3H, Ar-H), 7.86 (d, J = 8.6 Hz, 2H, Ar-H), 7.65 (d,J = 7.6 Hz, 1H, Ar-H), 7.52 (t, J = 7.9 Hz, 1H, Ar-H), 7.32 (s, 1H,Ar-H), 4.32 (d, J = 11.2 Hz, 4H, CH2CH2), 3.79 (d, J = 20.5 Hz, 4H,CH2CH2), 3.65 (s, 4H, CH2CH2); 13C NMR (150 MHz, DMSO‑d6): δ157.1, 156.5, 153.9, 150.3, 148.1, 148.0, 140.6, 136.3, 133.3, 130.8,129.6, 122.5, 122.4, 121.8, 121.0, 120.2, 119.3, 112.2, 110.7, 110.2,73.4,

70.9, 70.9, 70.5, 69.3, 68.9; HR MS (ESI) m/z: calcd forC28H25O4N6BrNa [M + Na]+ 611.1013, found 611.1020.

{3-[1-(2-Bromo-phenyl)-1H-[1,2,3]triazol-4-yl]-phenyl}-(7,8,10,11,13,14-hexahydro-6,9,12,15-tetraoxa-1,3-diaza-cyclododeca[b]naphthalen-4-yl)-amine (3k):

Yellow solid, yield85%, m.p. 129–132 ℃; 1H NMR (600 MHz, DMSO‑d6): δ 9.64 (s, 1H,CH), 9.04 (s, 1H, CH), 8.54 (s, 1H, NH), 8.45 (s, 1H, Ar-H), 8.22 (s,1H, Ar-H), 7.97 (d, J = 8.0 Hz, 2H, Ar-H), 7.77 (d, J = 7.8 Hz, 1H,Ar-H), 7.71–7.65 (m, 2H, Ar-H), 7.60 (dd, J1 = 17.2 Hz, J2 = 9.5 Hz,1H, Ar-H), 7.51 (t, J = 7.8 Hz, 1H, Ar-H), 7.32 (s, 1H, Ar-H), 4.32(d, J = 10.4 Hz, 4H, CH2CH2), 3.79 (d, J = 21.8 Hz, 4H, CH2CH2),3.65 (s, 4H, CH2CH2); 13C NMR (150 MHz, DMSO‑d6): δ 157.1, 156.5,153.9, 150.2, 148.1, 146.9, 140.6, 136.7, 134.1, 132.6, 130.9, 129.6,129.5, 129.2, 124.2, 122.4, 121.0, 119.5, 119.3, 112.1, 110.8, 110.2,73.4, 70.9, 70.8, 70.5, 69.3, 68.9; HR MS (ESI) m/z: calcd forC28H25O4N6BrNa [M + Na]+ 611.1013, found 611.1021.

(7,8,10,11,13,14-Hexahydro-6,9,12,15-tetraoxa-1,3-diaza-cyclododeca[b]naphthalen-4-yl)-{3-[1-(4-trifluoromethyl-phenyl)-1H-[1,2,3]triazol-4-yl]-phenyl}-amine (3l):

White solid, yield 77%, m.p. 144–147 ℃;1H NMR (600 MHz, DMSO‑d6): δ 9.69 (s, 1H, CH), 9.48 (s, 1H, CH), 8.45(s, 1H, NH), 8.36 (s, 1H, Ar-H), 8.25 (d, J = 8.0 Hz, 2H, Ar-H), 8.04 (d,J = 8.0 Hz, 2H, Ar-H), 7.97 (d, J = 7.5 Hz, 1H, Ar-H), 7.68–7.52 (m,3H, Ar-H), 4.32 (s, 4H, CH2CH2), 3.79 (d, J = 19.9 Hz, 4H, CH2CH2),3.64 (s, 4H, CH2CH2); 13C NMR (150 MHz, DMSO‑d6): δ 156.7, 156.3,150.4, 148.1, 140.7, 139.9, 130.7, 129.9, 129.3, 129.1, 128.8, 127.8,125.2, 123.4, 122.6, 121.6, 121.1, 120.9, 120.4, 119.4, 110.9, 73.4,70.9, 70.9, 70.5, 69.3, 68.9; HR MS (ESI) m/z: calcd for C29H26O4N6F3[M + H]+ 579.1962, found 579.1972.

{3-[1-(2-Fluoro-benzyl)-1H-[1,2,3]triazol-4-yl]-phenyl}-(7,8,10,11,13,14-hexahydro-6,9,12,15-tetraoxa-1,3-diaza-cyclododeca[b]naphthalen-4-yl)-amine (3m):

Yellow solid, yield93%, m.p. 118–121 ℃; 1H NMR (600 MHz, DMSO‑d6): δ 9.99 (s, 1H,CH), 9.05 (s, 1H, CH), 8.93 (s, 1H, NH), 8.73 (t, J = 1.8 Hz, 1H, Ar-H),8.61 (s, 1H, Ar-H), 8.36 (d, J = 7.3 Hz, 1H, Ar-H), 7.99 (d, J = 7.7 Hz,1H, Ar-H), 7.89–7.82 (m, 3H, Ar-H), 7.73–7.66 (m, 3H, Ar-H), 6.16(s, 2H, CH2), 4.73 (d, J = 16.9 Hz, 4H, CH2CH2), 4.23–4.18 (m, 4H,CH2CH2), 4.07 (s, 4H, CH2CH2); 13C NMR (150 MHz, DMSO‑d6): δ161.8, 160.2, 157.5, 156.9, 154.3, 150.6, 148.5, 147.5, 140.9, 131.7,131.7, 129.9, 125.8, 123.7, 122.6, 122.5, 121.2, 119.5, 116.6, 116.5,112.6, 111.1, 110.6, 73.8, 71.4, 71.3, 70.9, 69.7, 69.3, 48.0; HR MS(ESI) m/z: calcd for C29H28O4N6F [M + H]+ 543.2151, found543.2158.

(7,8,10,11,13,14-Hexahydro-6,9,12,15-tetraoxa-1,3-diaza-cyclododeca[b]naphthalen-4-yl)-{3-[1-(2-methyl-benzyl)-1H-[1,2,3]triazol-4-yl]-phenyl}-amine (3n):

Yellow solid, yield 82%, m.p. 114–117 ℃;1H NMR (600 MHz, DMSO‑d6): δ 9.61 (s, 1H, CH), 8.70 (s, 1H, NH), 8.56(s, 1H, CH), 8.31 (s, 1H, Ar-H), 8.25 (s, 1H, Ar-H), 7.93 (d, J = 8.0 Hz,1H, Ar-H), 7.57 (d, J = 7.6 Hz, 1H, Ar-H), 7.45 (t, J = 7.9 Hz,

1H,Ar-H), 7.37 (s, 1H, Ar-H), 7.28–7.21 (m, 3H, Ar-H), 7.16 (d, J = 7.5 Hz,1H, Ar-H), 5.68 (s, 2H, CH2), 4.31 (s, 4H, CH2CH2), 3.78 (d,J = 24.3 Hz, 4H, CH2CH2), 3.64 (s, 4H, CH2CH2), 2.36 (s, 3H, CH3); 13CNMR (150 MHz, DMSO‑d6): δ 157.1, 156.4, 153.7, 150.3, 147.0, 140.5,136.8, 134.6, 131.4, 130.9, 129.5, 129.2, 128.8, 126.8, 122.1, 120.9,119.1, 112.3, 110.8, 73.4, 70.9, 70.9, 70.5, 69.3, 68.9, 51.7, 19.2;HR MS (ESI) m/z: calcd for C30H31O4N6 [M + H]+ 539.2401, found539.2406.

(7,8,10,11,13,14-Hexahydro-6,9,12,15-tetraoxa-1,3-diaza-cyclododeca[b]naphthalen-4-yl)-{3-[1-(4-methyl-benzyl)-1H-[1,2,3]triazol-4-yl]-phenyl}-amine (3o):

Brown solid, yield 75%, m.p. 124–127 ℃;1H NMR (600 MHz, DMSO‑d6): δ 9.59 (s, 1H, CH), 8.61 (s, 1H, CH),8.30 (s, 1H, Ar-H), 8.24 (s, 1H, Ar-H), 7.93 (d, J = 8.0 Hz, 1H, Ar-H), 7.55 (d, J = 7.6 Hz, 1H, Ar-H),7.45 (t, J = 7.9 Hz, 1H, Ar-H), 7.35 (s, 1H, Ar-H), 7.28 (d, J = 7.7 Hz,2H, Ar-H), 7.21 (d, J = 7.7 Hz, 2H, Ar-H), 5.61 (s, 2H, CH2), 4.31(s, 4H, CH2CH2), 3.79 (d, J = 23.8 Hz, 4H, CH2CH2), 3.65 (s, 4H,CH2CH2), 2.30 (s, 3H, CH3); 13C NMR (150 MHz, DMSO‑d6): δ 157.1,156.5, 153.7, 150.3, 147.1, 140.5, 138.0, 133.5, 131.4, 129.8, 129.5,128.5, 122.1, 121.9, 120.8, 119.1, 112.3, 110.8, 73.4, 70.9, 70.9, 70.5,69.3, 68.9, 53.3, 21.2; HR MS (ESI) m/z: calcd for C30H31O4N6[M + H]+ 539.2401, found 539.2408.

(7,8,10,11,13,14-Hexahydro-6,9,12,15-tetraoxa-1,3-diaza-cyclododeca[b]naphthalen-4-yl)-{3-[1-(2-iodo-benzyl)-1H-[1,2,3]triazol-4-yl]-phenyl}-amine (3p):

Yellow solid, yield 89%, m.p. 121–123 ℃;1H NMR (600 MHz, DMSO‑d6): δ 9.61 (s, 1H, CH), 8.57 (d, J = 16.0 Hz,2H, CH, NH), 8.32 (s, 1H, Ar-H), 8.21 (s, 1H, Ar-H), 7.95 (dd,J1 = 13.5 Hz, J2 = 8.0 Hz, 2H, Ar-H), 7.58 (d, J = 7.6 Hz, 1H, Ar-H),7.45 (dd, J1 = 14.9 Hz, J2 = 7.5 Hz, 2H, Ar-H), 7.32 (s, 1H, Ar-H),7.17–7.13 (m, 2H, Ar-H), 5.70 (s, 2H, CH2), 4.31 (s, 4H, CH2CH2),3.79 (d, J = 23.6 Hz, 4H, CH2CH2), 3.65 (s, 4H, CH2CH2); 13C NMR(101 MHz, DMSO‑d6): δ 157.12, 156.48, 153.74, 150.25, 147.91,146.95, 140.47, 140.00, 138.37, 131.33, 130.78, 130.15, 129.51,129.37, 122.49, 122.14, 120.90, 119.14, 112.01, 110.77, 110.31, 99.69,73.43, 70.94, 70.84, 70.46, 69.27, 68.87, 58.03; HR MS (ESI) m/z: calcdfor C29H28O4N6I [M + H]+ 651.1211, found 651.1220.

{3-[1-(3-Bromo-benzyl)-1H-[1,2,3]triazol-4-yl]-phenyl}-(7,8,10,11,13,14-hexahydro-6,9,12,15-tetraoxa-1,3-diaza-cyclododeca[b]naphthalen-4-yl)-amine (3q):

White solid, yield 70%,m.p. 113–116 ℃; 1H NMR (600 MHz, DMSO‑d6): δ 9.59 (s, 1H, CH),8.68 (s, 1H, CH), 8.53 (s, 1H, NH), 8.32 (s, 1H, Ar-H), 8.20 (s, 1H, Ar-H),7.93 (d, J = 8.0 Hz, 1H, Ar-H), 7.62 (s, 1H, Ar-H), 7.56 (d, J = 6.9 Hz,2H, Ar-H), 7.46 (t, J = 7.9 Hz, 1H, Ar-H), 7.38 (d, J = 4.4 Hz, 2H,Ar-H), 7.31 (s, 1H, Ar-H), 5.69 (s, 2H, CH2), 4.31 (s, 4H, CH2CH2),3.79 (d, J = 22.7 Hz, 4H, CH2CH2), 3.65 (s, 4H, CH2CH2); 13C NMR(101 MHz, DMSO‑d6): δ 157.1, 156.5, 153.8, 150.3, 148.0, 147.2, 140.5,139.1, 131.6, 131.5, 131.2, 129.5, 127.6, 122.4, 122.3, 122.1, 120.8,119.1, 112.1, 110.7, 110.3, 73.4, 70.9, 70.8, 70.5, 69.3, 68.9, 52.7; HRMS (ESI) m/z: calcd for C29H28O4N6Br [M + H]+ 603.1350, found603.1356.

[3-(1-Benzyl-1H-[1,2,3]triazol-4-yl)-phenyl]-(7,8,10,11,13,14-hexahydro-6,9,12,15-tetraoxa-1,3-diaza-cyclododeca[b]naphthalen-4-yl)-amine(3r):

Brown solid, yield 74%, m.p. 146–149 ℃; 1H NMR (600 MHz,DMSO‑d6): δ 9.60 (s, 1H, CH), 8.66 (s, 1H, CH), 8.51 (s, 1H, NH), 8.32 (s,1H, Ar-H), 8.21 (s, 1H, Ar-H), 7.93 (d, J = 7.9 Hz, 1H, Ar-H), 7.56(d, J = 7.6 Hz, 1H, Ar-H), 7.47–7.35 (m, 6H), 7.31 (s, 1H, Ar-H),5.67 (s, 2H, CH2), 4.31 (s, 4H, CH2CH2), 3.81–3.76 (m, 4H, CH2CH2),3.65 (s, 4H, CH2CH2); 13C NMR (150 MHz, DMSO‑d6): δ 157.1, 156.4,153.9, 150.2, 148.1, 147.1, 140.5, 136.5, 131.4, 129.5, 129.3, 128.7,128.4, 122.1, 122.1, 120.8, 119.1, 112.1, 110.7, 73.4, 70.9, 70.9, 70.5,69.3, 68.9, 53.5; HR MS (ESI) m/z: calcd for C29H29O4N6 [M + H]+525.2245, found 525.2254.

{3-[1-(2-Bromo-benzyl)-1H-[1,2,3]triazol-4-yl]-phenyl}-(7,8,10,11,13,14-hexahydro-6,9,12,15-tetraoxa-1,3-diaza-cyclododeca[b]naphthalen-4-yl)-amine (3s):

White solid, yield 86%,m.p. 161–164 ℃; 1H NMR (600 MHz, DMSO‑d6): δ 9.59 (s, 1H, CH),8.62 (s, 1H, CH), 8.51 (s, 1H, NH), 8.32 (s, 1H, Ar-H), 8.20 (s, 1H, Ar-H),7.94 (d, J = 8.3 Hz, 1H, Ar-H), 7.73 (d, J = 7.8 Hz, 1H, Ar-H), 7.57(d, J = 7.5 Hz, 1H, Ar-H), 7.45 (dd, J1 = 14.4 Hz, J2 = 7.4 Hz, 2H,Ar-H), 7.35 (t, J = 7.7 Hz, 1H, Ar-H), 7.31 (s, 1H, Ar-H), 7.26 (d,J = 7.7 Hz, 1H, Ar-H), 5.76 (s, 2H, CH2), 4.31 (s, 4H, CH2CH2), 3.79 (d,J = 23.7 Hz, 4H, CH2CH2), 3.63 (s, 4H, CH2CH2); HR MS (ESI) m/z:calcd for C29H27O4N6BrNa [M + Na]+ 625.1169, found 625.1178.

(7,8,10,11,13,14-Hexahydro-6,9,12,15-tetraoxa-1,3-diaza-cyclododeca[b]naphthalen-4-yl)-{3-[1-(3-methoxy-benzyl)-1H-[1,2,3]triazol-4-yl]-phenyl}-amine (3t):

Yellow solid, yield 74%, m.p. 131–134 ℃;1H NMR (600 MHz, DMSO‑d6): δ 9.72 (s, 1H, CH), 8.66 (s, 1H, CH), 8.50(s, 1H, NH), 8.35 (s, 1H, Ar-H), 8.31 (s, 1H, Ar-H), 7.95 (d, J = 8.0 Hz,1H, Ar-H), 7.55 (d, J = 7.5 Hz, 1H, Ar-H), 7.44 (t, J = 7.9 Hz, 1H,Ar-H), 7.35–7.28 (m, 2H, Ar-H), 6.98 (s, 1H, Ar-H), 6.93 (d, J = 7.9 Hz,2H, Ar-H), 5.63 (s, 2H, CH2), 4.32 (d, J = 23.7 Hz, 4H, CH2CH2), 3.80(s, 2H, CH2), 3.76 (s, 5H, CH2, OCH3), 3.64 (s, 4H, CH2CH2); 13C NMR(150 MHz, DMSO‑d6): δ 159.9, 157.1, 156.4, 153.9, 150.2, 148.1, 147.1,140.6, 137.9, 131.3, 130.5, 129.4, 122.1, 120.7, 120.5, 119.2, 114.3,114.0, 112.2, 110.8, 110.3, 73.3, 70.9, 70.4, 69.2, 68.8, 55.6, 53.4; HRMS (ESI) m/z: calcd for C30H30O5N6Na [M + Na]+ 577.2170, found577.2176.

{3-[1-(3,5-Dibromo-benzyl)-1H-[1,2,3]triazol-4-yl]-phenyl}-(7,8,10,11,13,14-hexahydro-6,9,12,15-tetraoxa-1,3-diaza-cyclododeca[b]naphthalen-4-yl)-amine (3u):

Yellow solid, yield88%, m.p. 151–154 ℃; 1H NMR (600 MHz, DMSO‑d6): δ 9.62 (s, 1H,CH), 8.71 (s, 1H, CH), 8.51 (s, 1H, NH), 8.33 (s, 1H, Ar-H), 8.22 (s, 1H,Ar-H), 7.93 (d, J = 8.2 Hz, 1H, Ar-H), 7.86 (s, 1H, Ar-H), 7.64 (s, 2H,Ar-H), 7.57 (d, J = 7.5 Hz, 1H, Ar-H), 7.46 (t, J = 7.8 Hz, 1H, Ar-H),7.31 (s, 1H, Ar-H), 5.70 (s, 2H, CH2), 4.32 (s, 4H, CH2CH2), 3.78 (d,J = 26.7 Hz, 4H, CH2CH2), 3.65 (s, 4H, CH2CH2); 13C NMR (150 MHz,DMSO‑d6): δ 157.1, 156.4, 153.9, 150.2, 148.1, 147.2, 140.8, 140.5,133.7, 131.2, 130.7, 129.5, 123.2, 122.4, 122.2, 120.8, 119.1, 112.2,110.7,

110.2, 73.4, 70.9, 70.9, 70.5, 69.2, 68.9, 52.0; HR MS (ESI) m/z:calcd for $C_{29}H_{26}O_4N_6Br_2Na$ [M + Na]+703.0274, found 703.0281.

(3-{1-[2-(4-Fluoro-phenyl)-ethyl]-1H-[1,2,3]triazol-4-yl}-phenyl)-(7,8,10,11,13,14-hexahydro-6,9,12,15-tetraoxa-1,3-diaza-cyclododeca[b]naphthalen-4-yl)-amine (3v):

Brown solid, yield 79%,m.p. 110–113 ℃; 1H NMR (600 MHz, DMSO‑d6): δ 9.58 (s, 1H, CH),8.52 (s, 2H, CH, NH), 8.30 (s, 1H, Ar-H), 8.20 (s, 1H, Ar-H), 7.92(d, J = 7.9 Hz, 1H, Ar-H), 7.51 (d, J = 7.5 Hz, 1H, Ar-H), 7.45 (t,J = 7.8 Hz, 1H, Ar-H), 7.32 (s, 1H, Ar-H), 7.29–7.16 (m, 2H, Ar-H),7.12 (t, J = 8.7 Hz, 2H, Ar-H), 4.67 (t, J = 7.2 Hz, 2H, CH2), 4.31(s, 4H, CH2CH2), 3.79 (d, J = 21.0 Hz, 4H, CH2CH2), 3.65 (s, 4H,CH2CH2), 3.24 (t, J = 7.2 Hz, 2H, CH2); 13C NMR (150 MHz, DMSO‑d6):δ 162.4, 160.7, 157.1, 156.4, 153.9, 150.2, 148.1, 148.1, 146.6, 140.5,134.3, 131.5, 131.1, 131.0, 121.9, 121.9, 120.7, 119.0, 115.7, 115.6,112.2, 110.7, 110.2, 73.4, 70.9, 70.9, 70.5, 69.3, 68.9, 51.1, 35.2; HRMS (ESI) m/z: calcd for $C_{30}H_{29}O_4N_6FNa$ [M + Na]+ 579.2127, found579.2134.

Figure S1-1. ^{1}H NMR spectrum (600 MHz, DMSO-d_6) of compound 3a

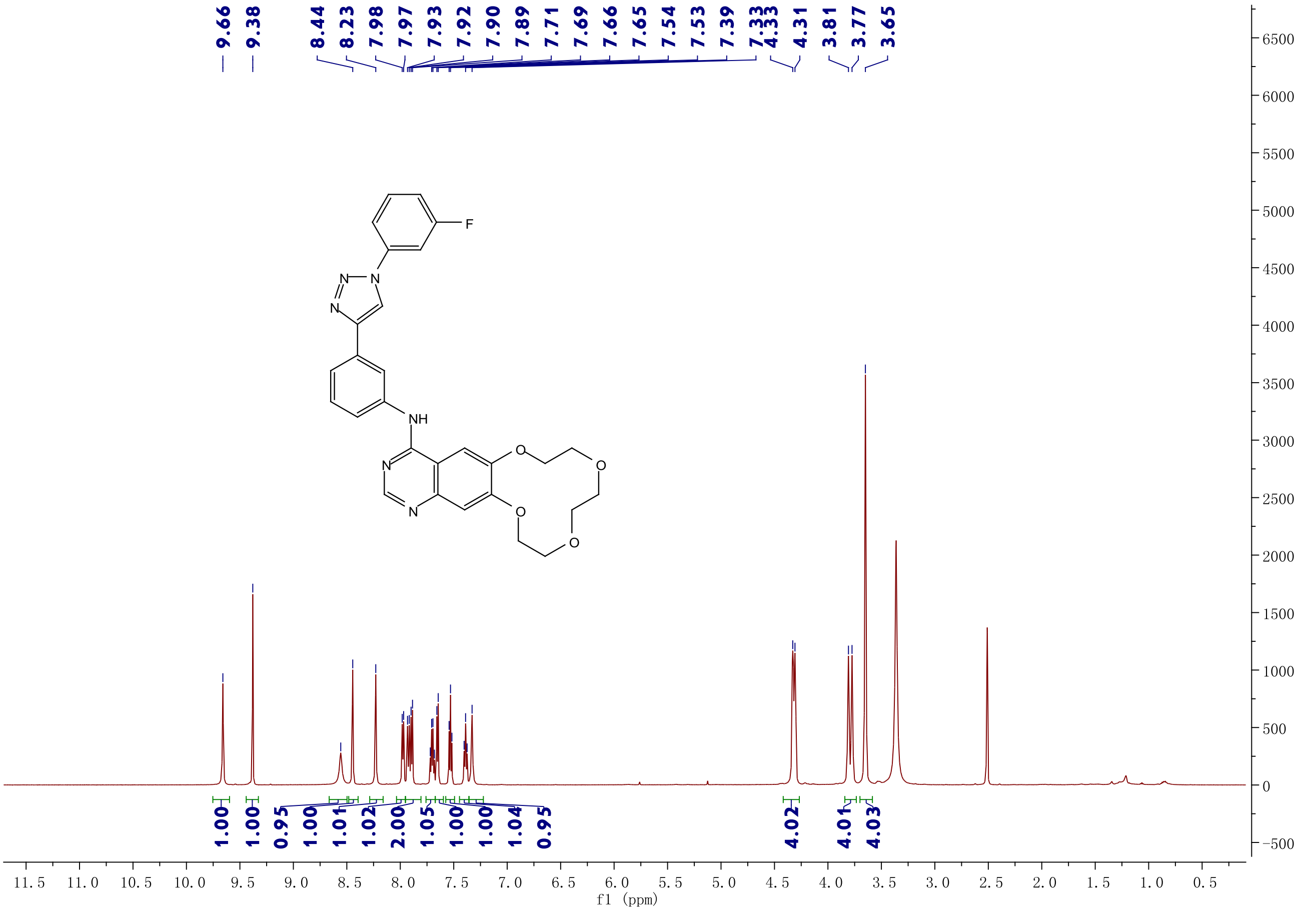

Figure S1-2. ^{13}C NMR spectrum (150 MHz, DMSO-d_6) of compound 3a

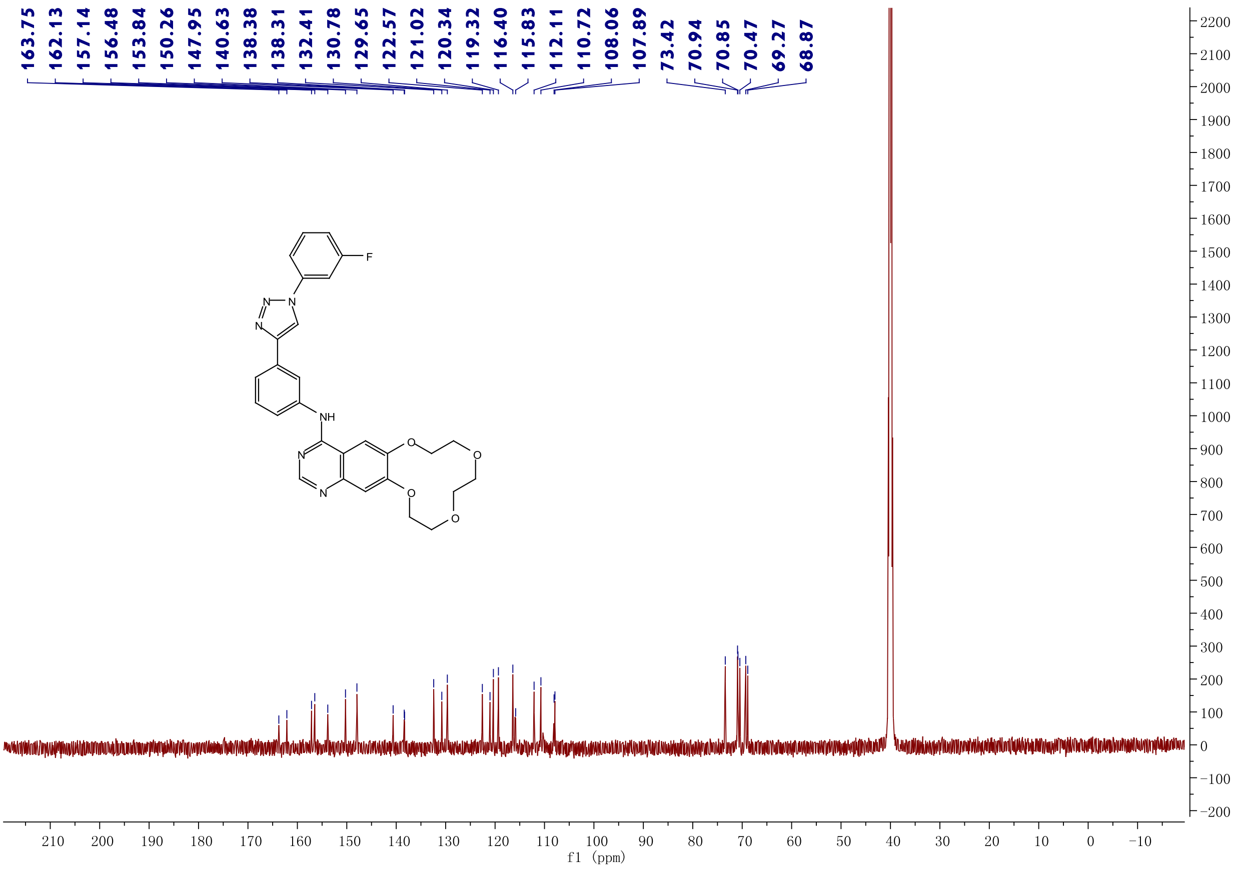

Figure S1-3. HR MS of compound 3a

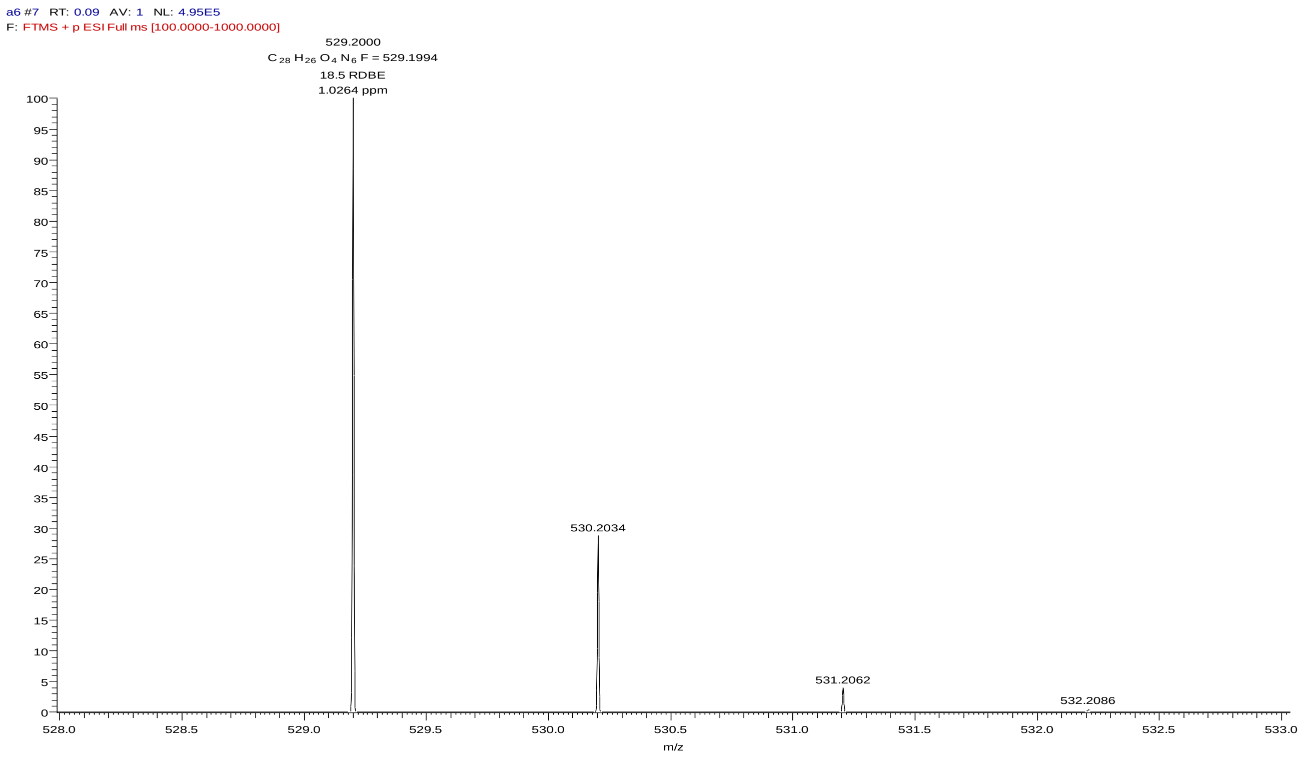

Figure S2-1. ^{1}H NMR spectrum (600 MHz, DMSO-d_6) of compound 3b

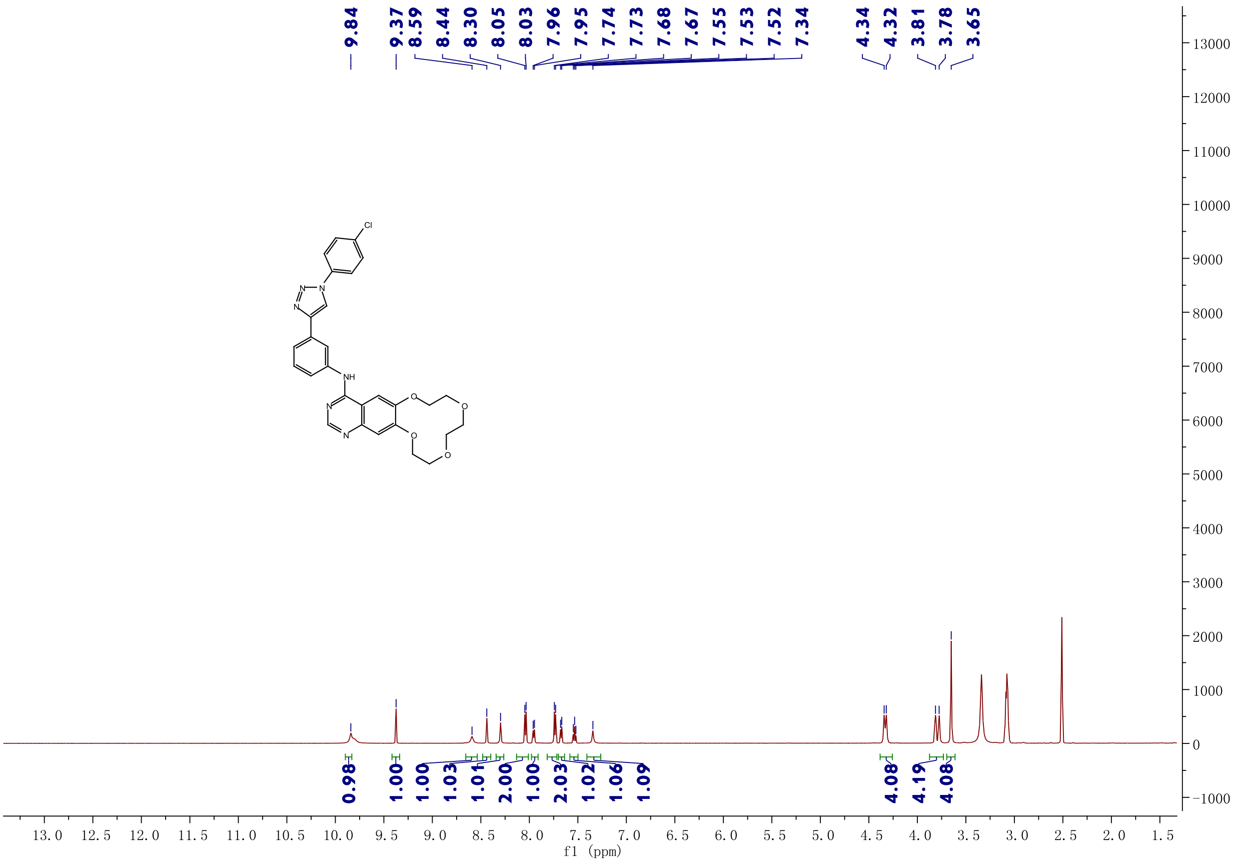

Figure S2-2. HR MS of compound 3b

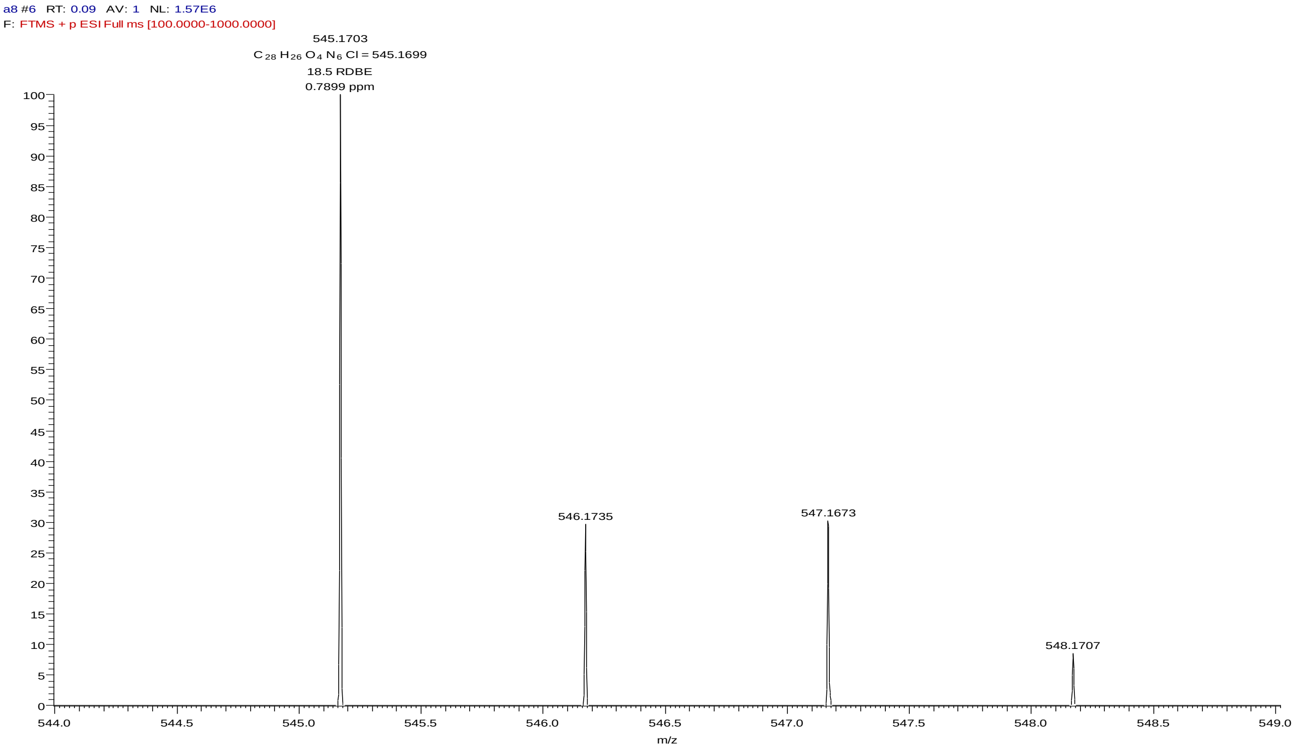

Figure S3-1. ^{1}H NMR spectrum (600 MHz, DMSO-d_6) of compound 3c

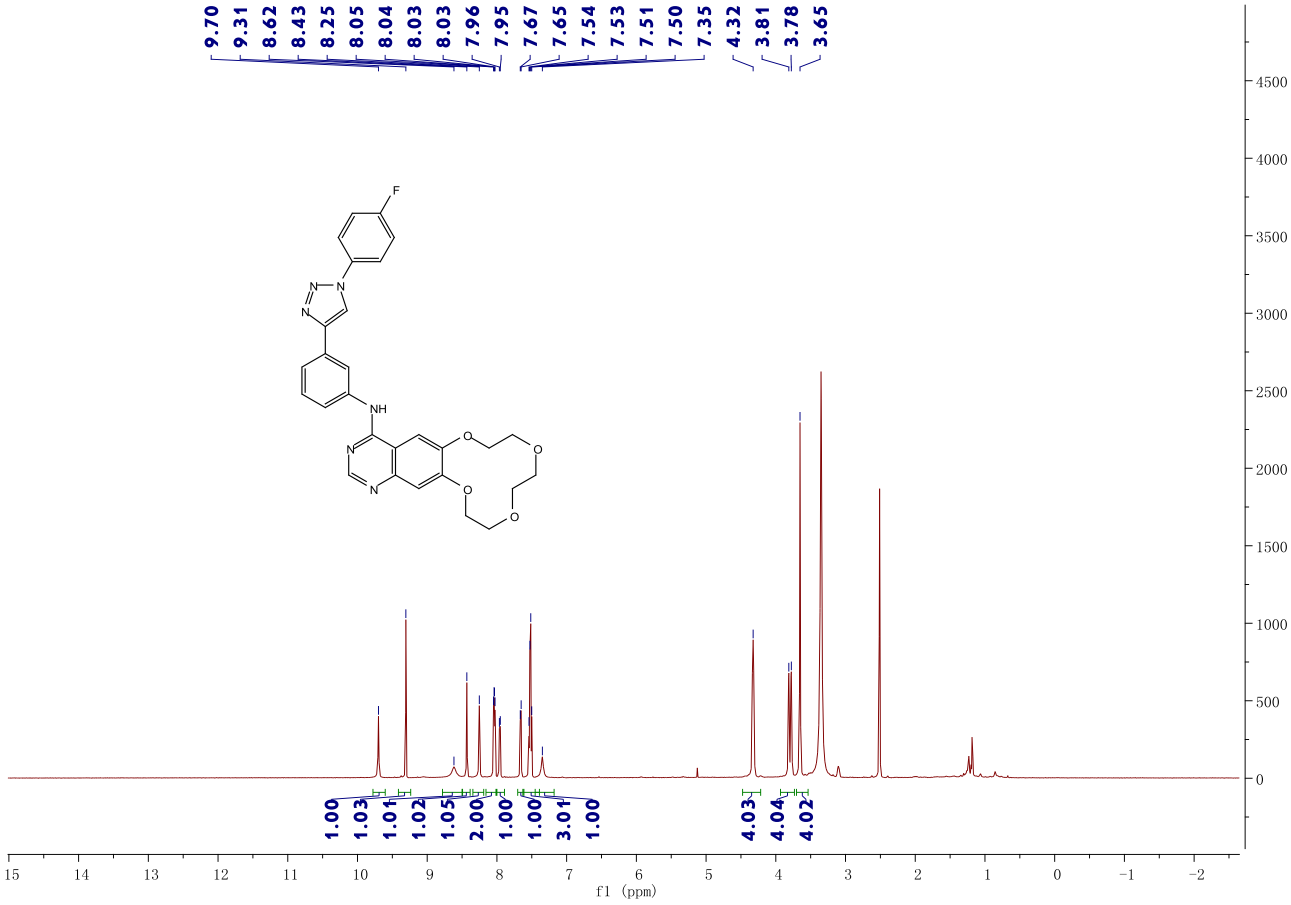

Figure S3-2. ^{13}C NMR spectrum (150 MHz, DMSO-d_6) of compound 3c

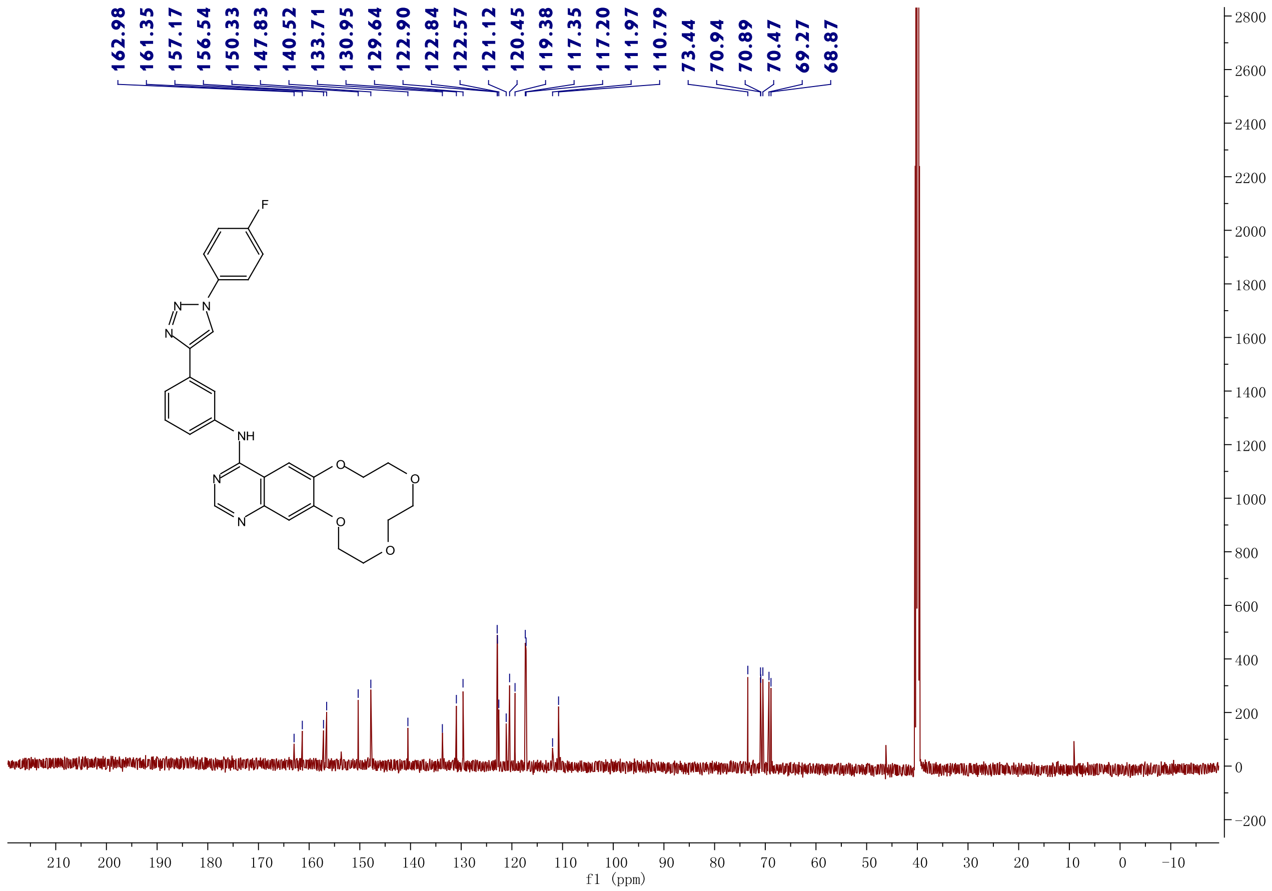

Figure S3-3. HR MS of compound 3c

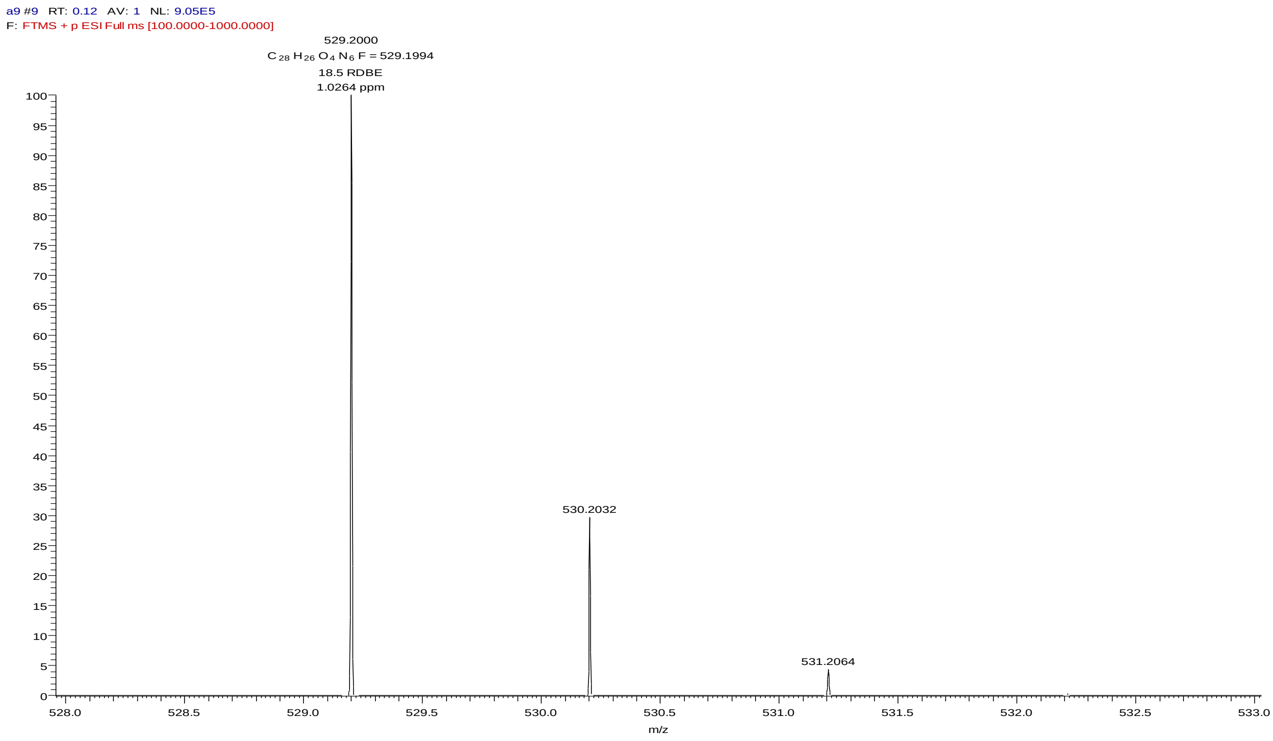

Figure S4-1. ^{1}H NMR spectrum (600 MHz, DMSO-d_6) of compound 3d

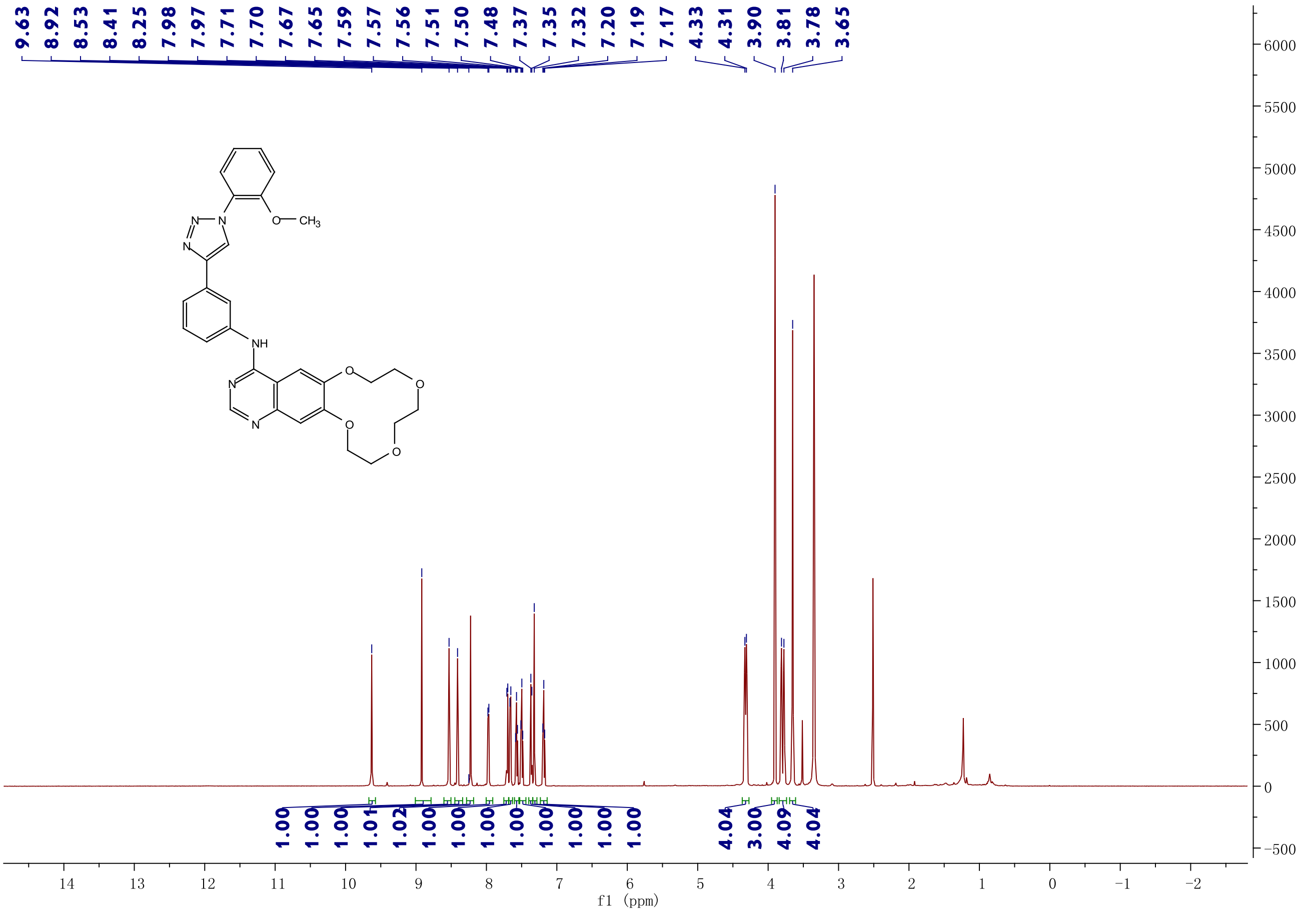

Figure S4-2. ^{13}C NMR spectrum (150 MHz, DMSO-d_6) of compound 3d

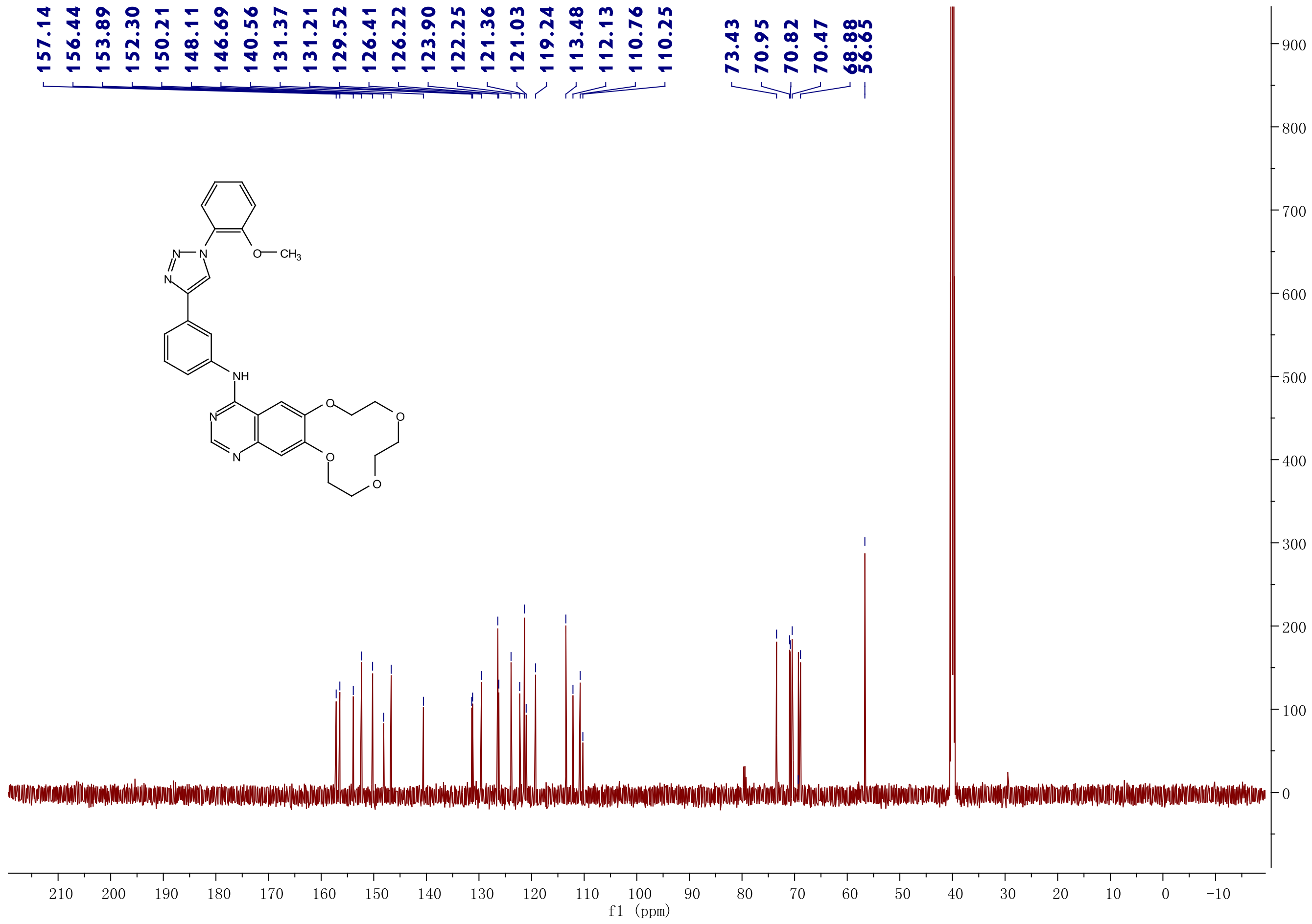

Figure S4-3. HR MS of compound 3d

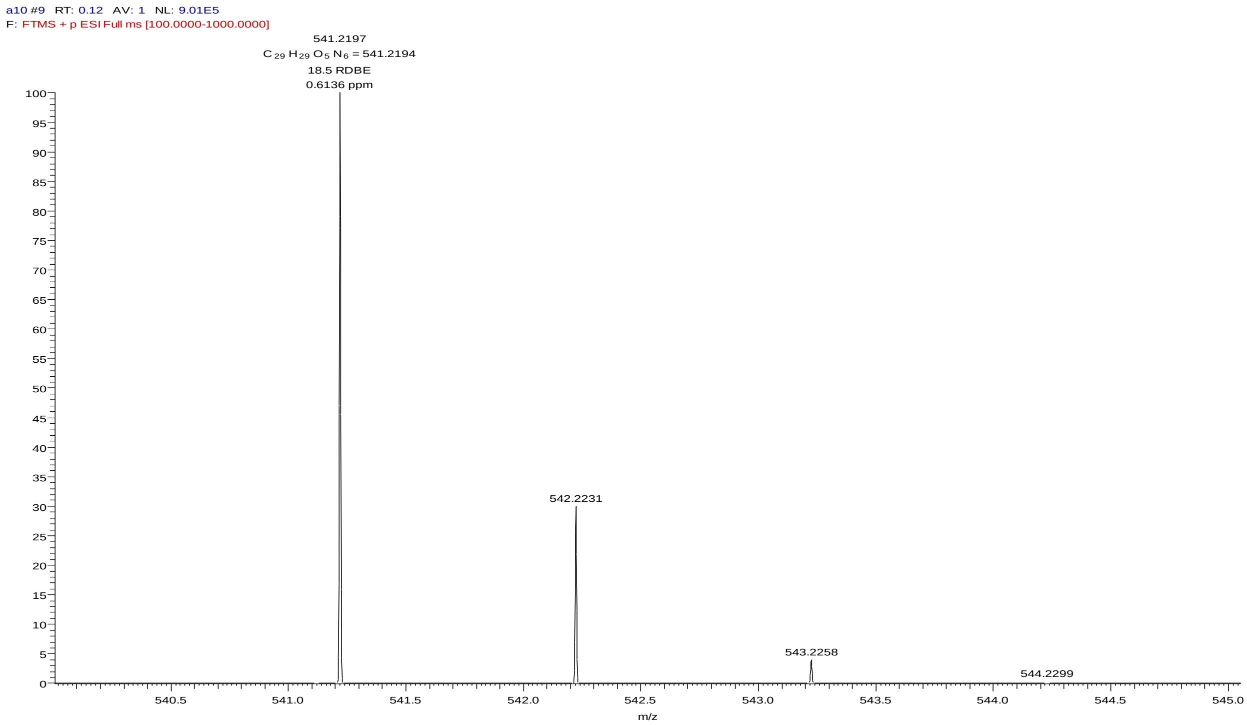

Figure S5-1. ^{1}H NMR spectrum (600 MHz, DMSO-d_6) of compound 3e

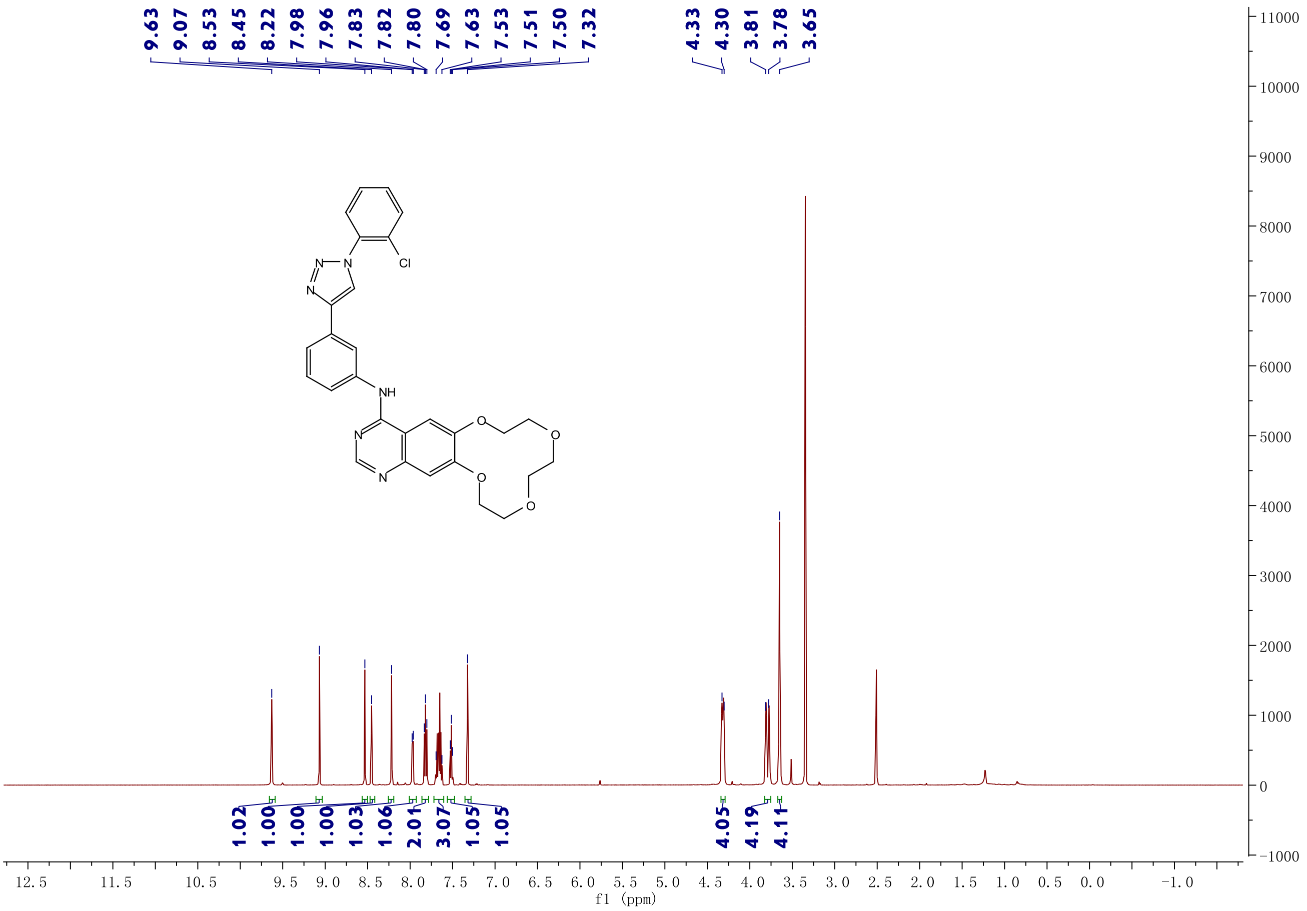

Figure S5-2. ^{13}C NMR spectrum (150 MHz, DMSO-d_6) of compound 3e

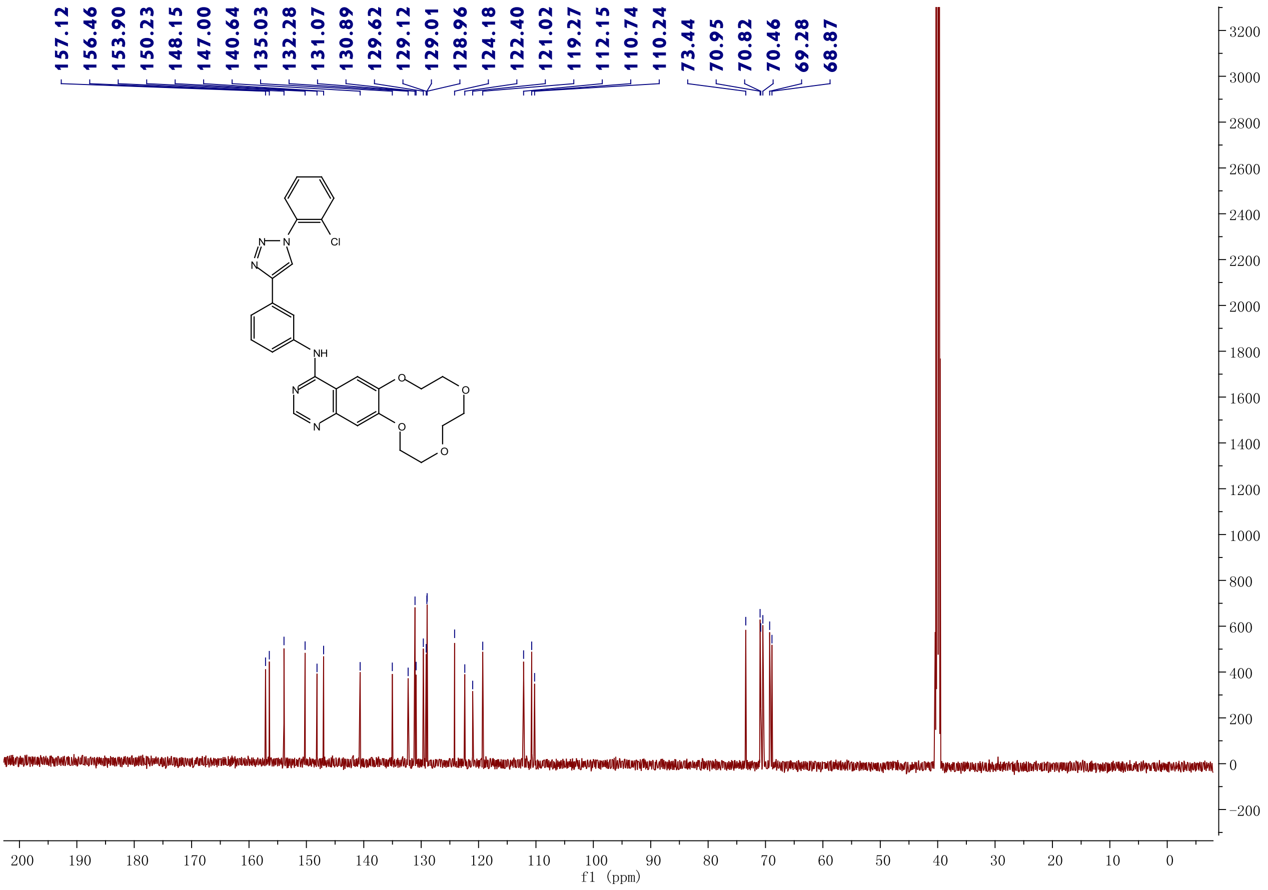

Figure S5-3. HR MS of compound 3e

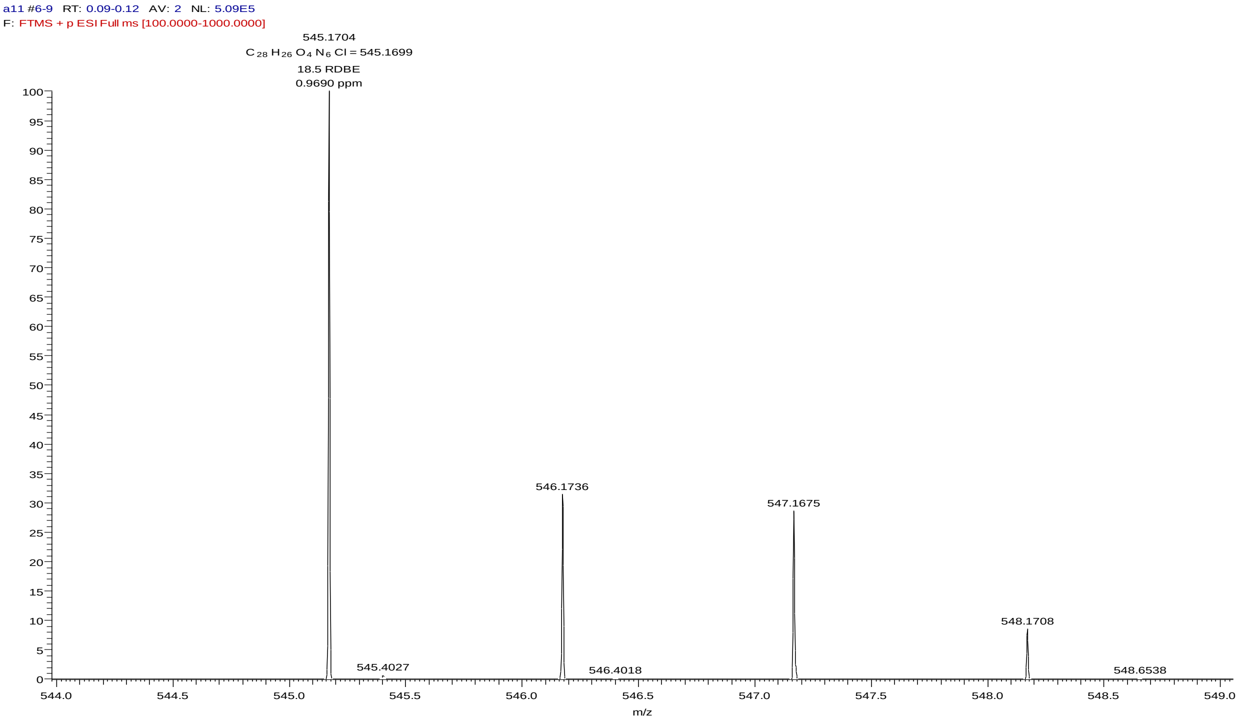

Figure S6-1. ^{1}H NMR spectrum (600 MHz, DMSO-d_6) of compound 3f

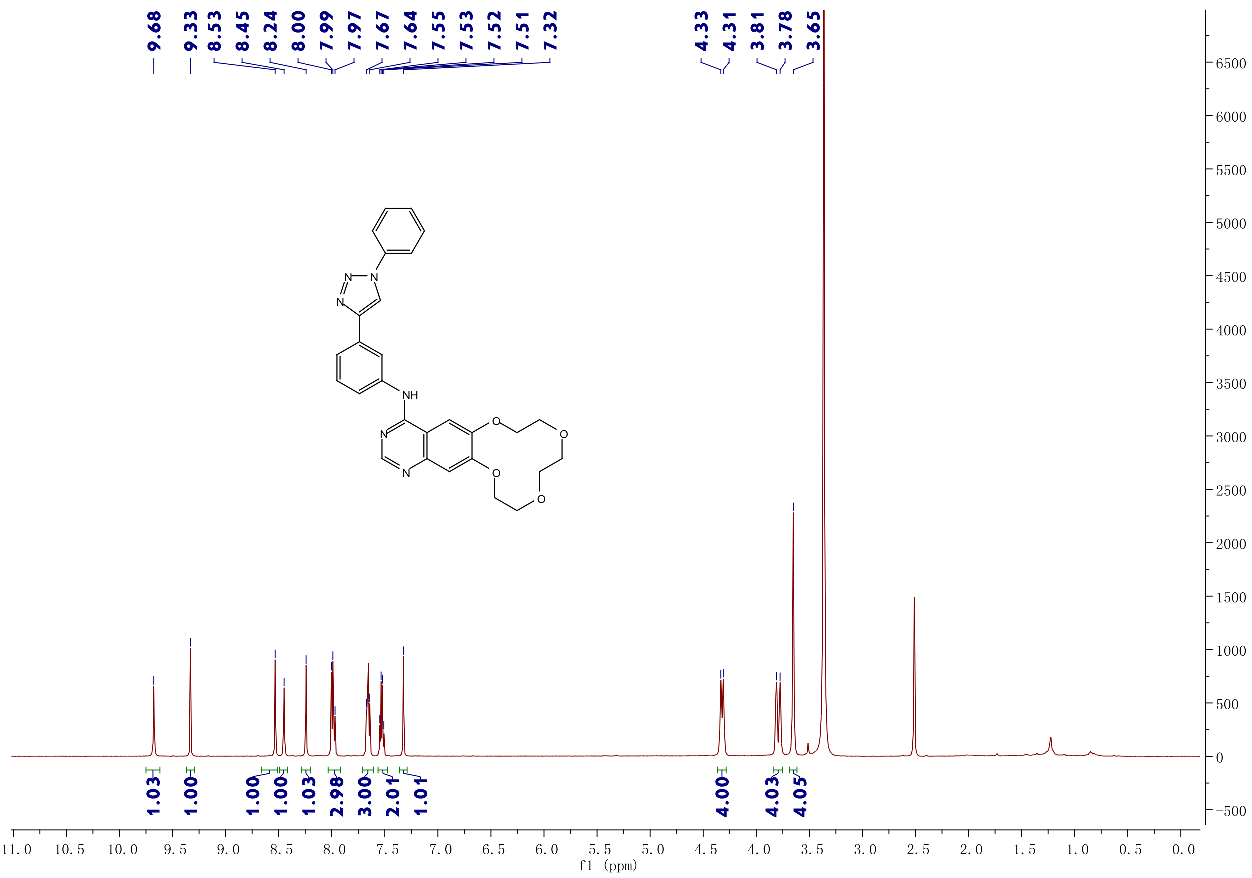

Figure S6-2. ^{13}C NMR spectrum (150 MHz, DMSO-d_6) of compound 3f

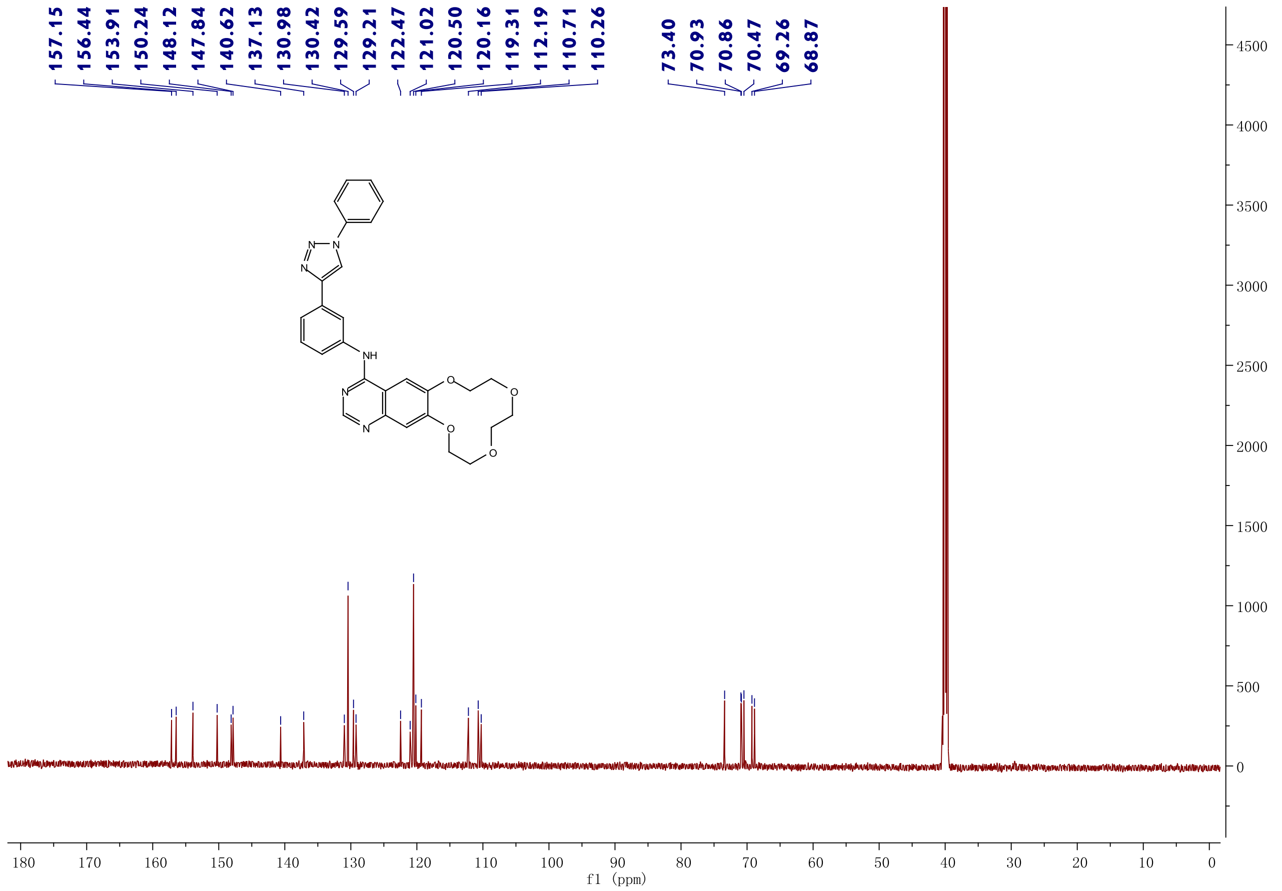

Figure S6-3. HR MS of compound 3f

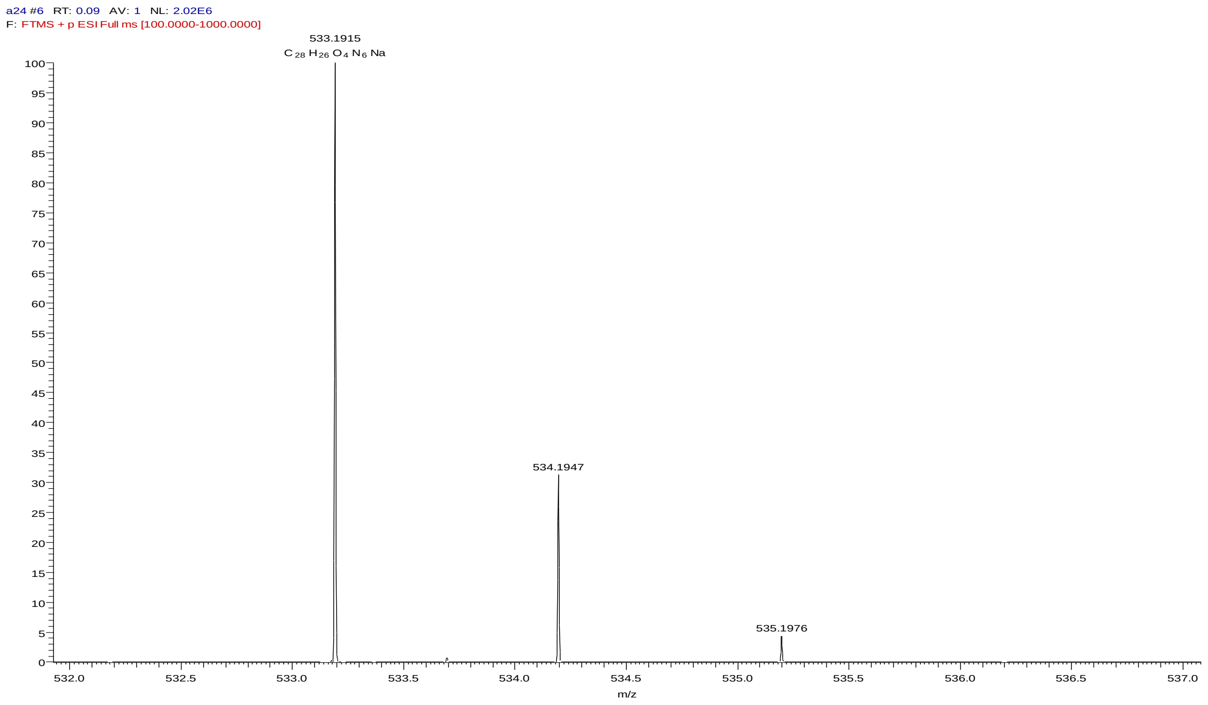

Figure S7-1. ^{1}H NMR spectrum (600 MHz, DMSO-d_6) of compound 3g

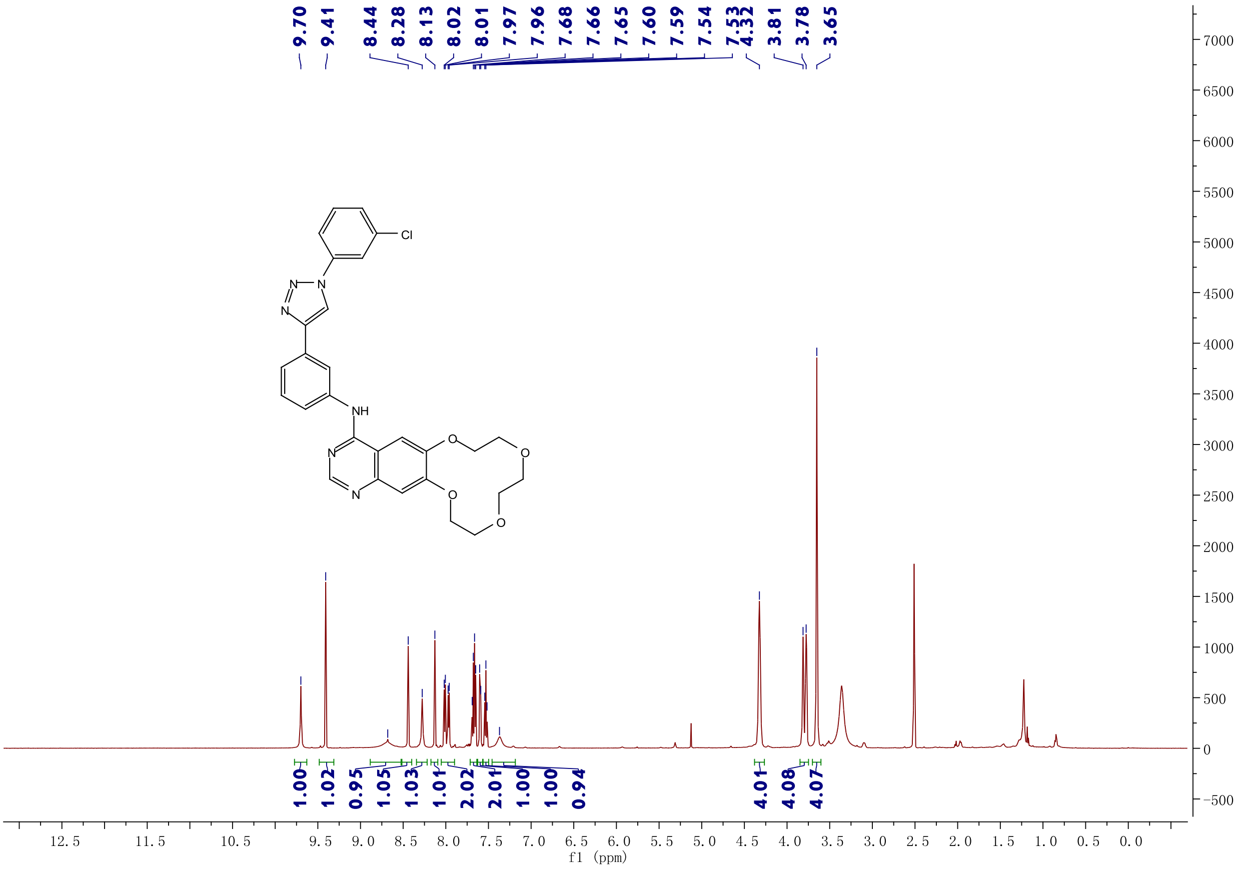

Figure S7-2. ^{13}C NMR spectrum (150 MHz, DMSO-d_6) of compound 3g

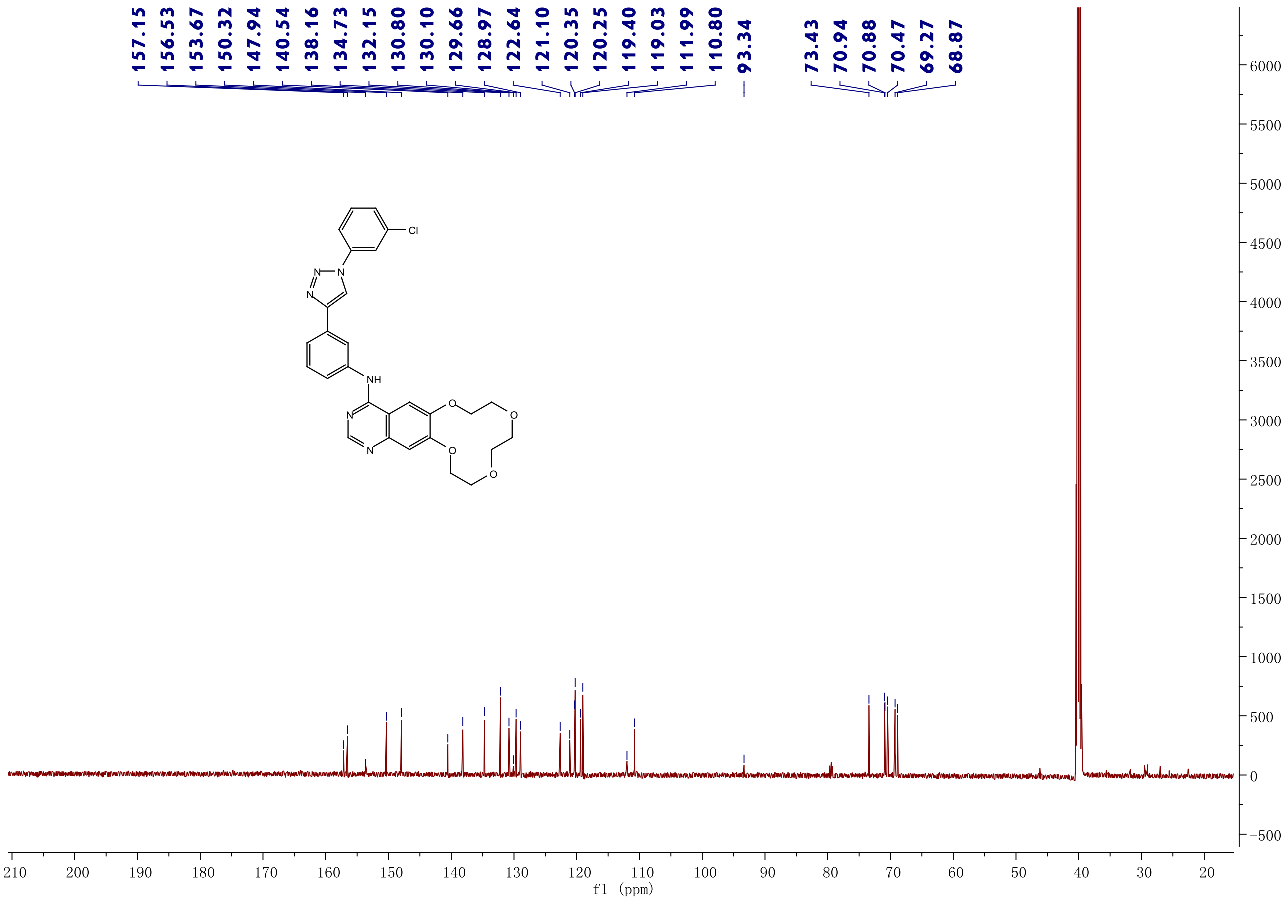

Figure S7-3. HR MS of compound 3g

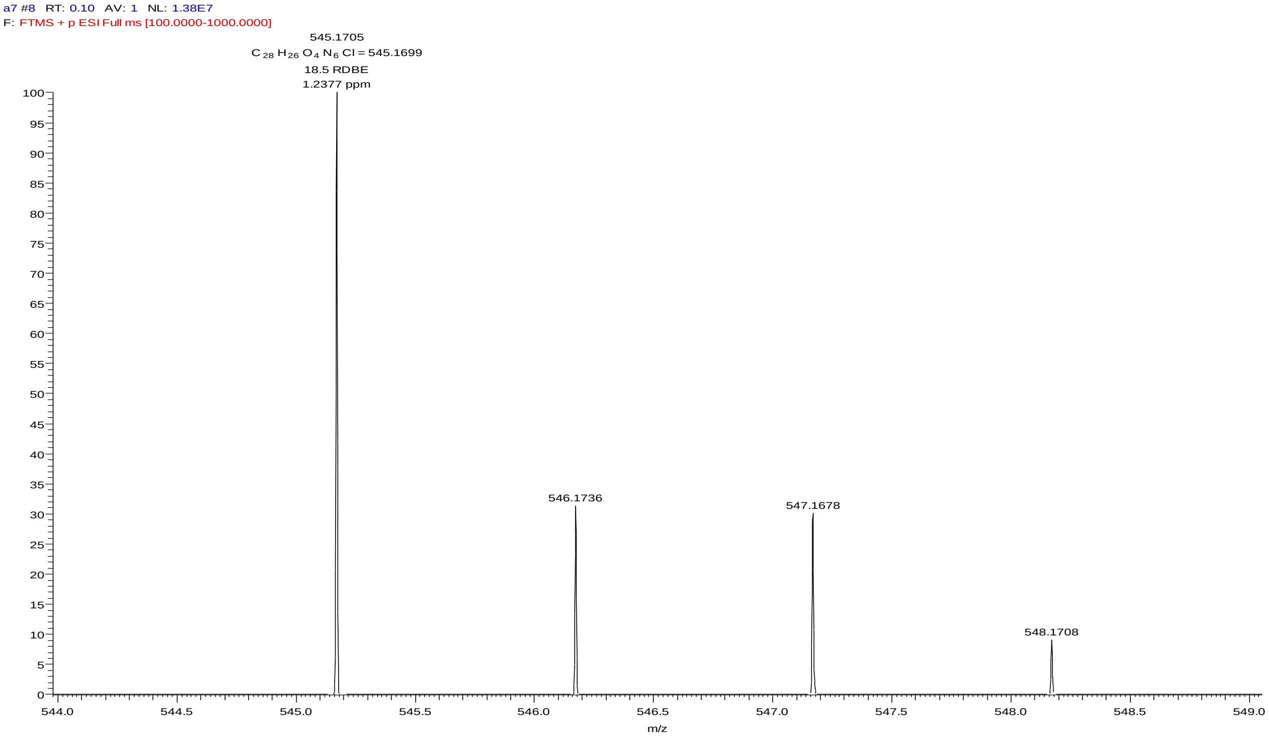

Figure S8-1. ^{1}H NMR spectrum (600 MHz, DMSO-d_6) of compound 3h

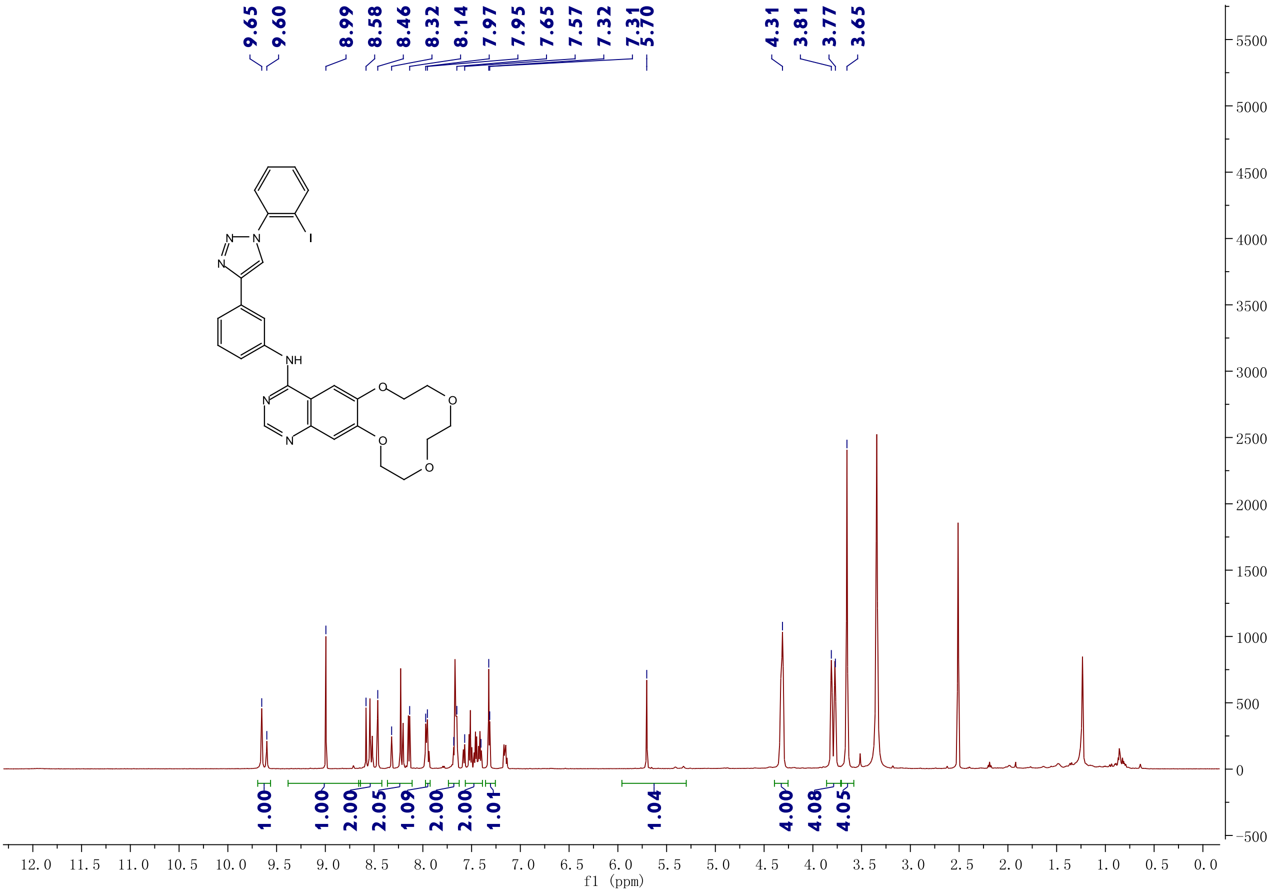

Figure S8-2. ^{13}C NMR spectrum (150 MHz, DMSO-d_6) of compound 3h

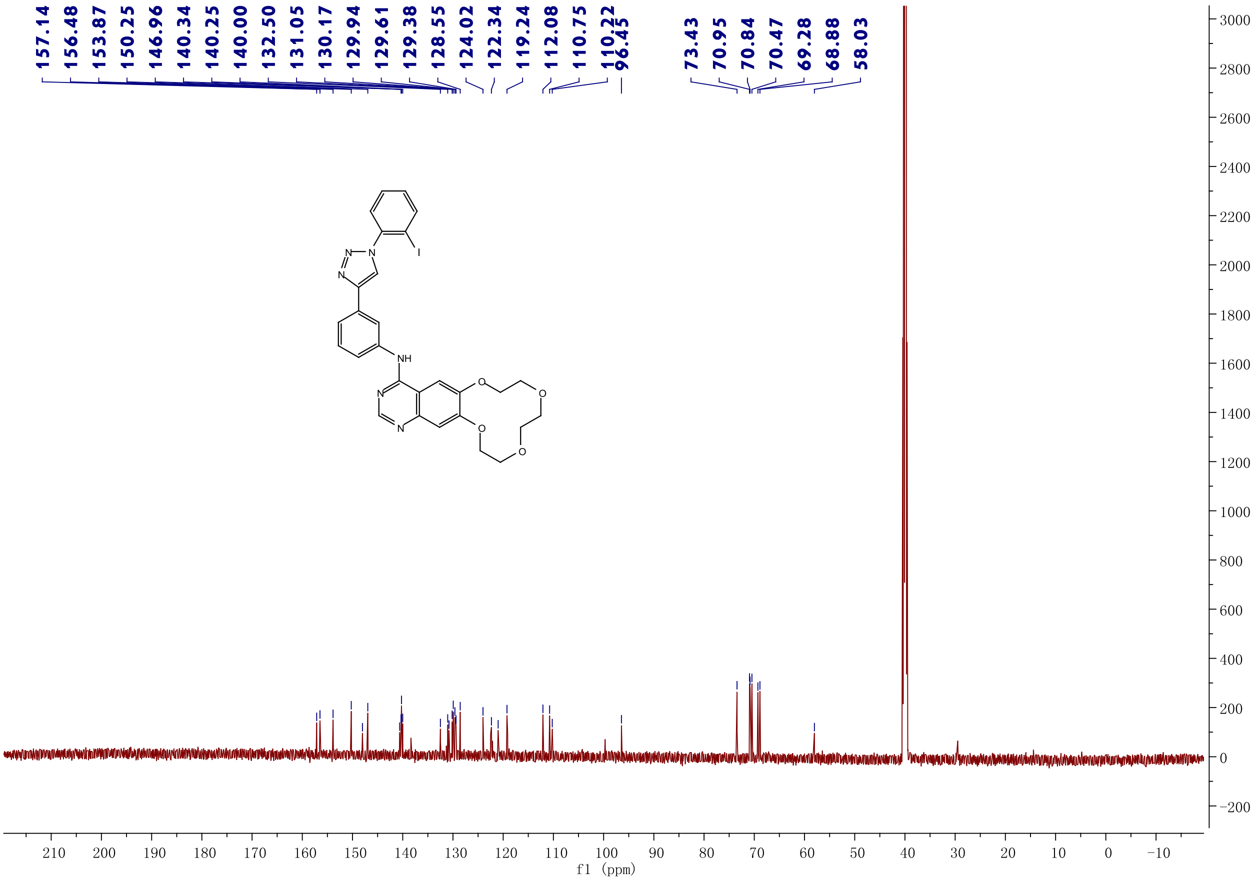

Figure S8-3. HR MS of compound 3h

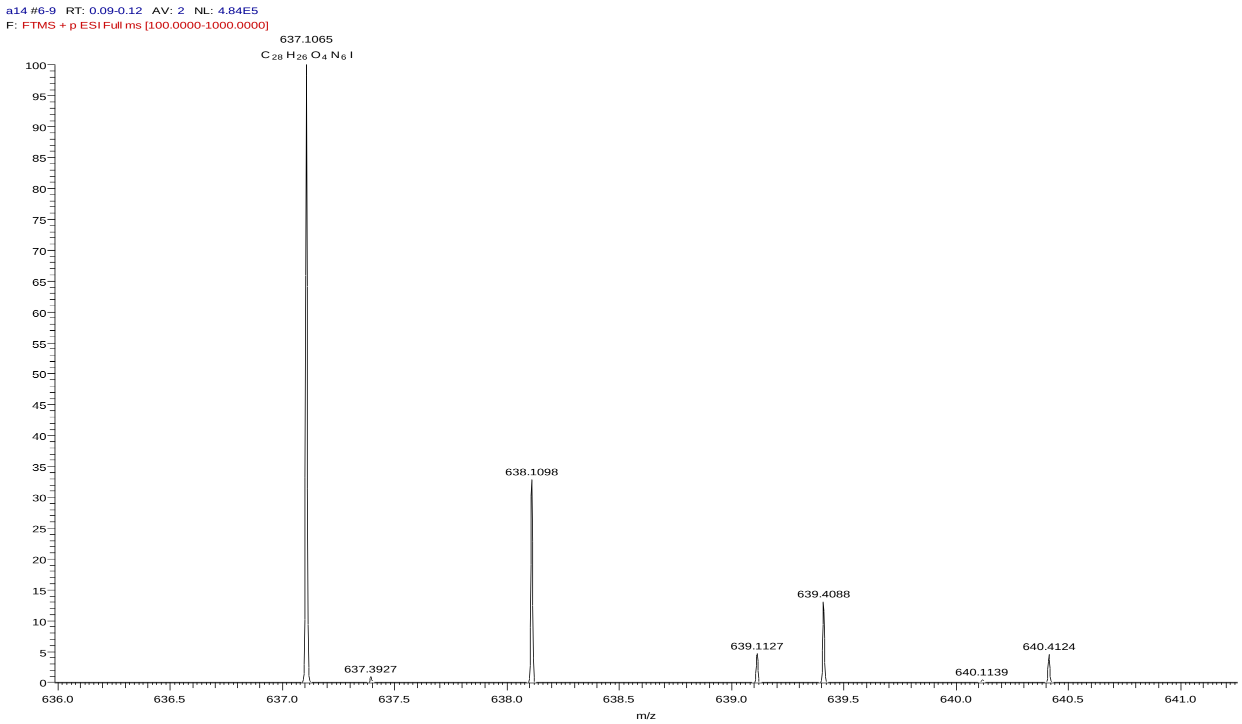

Figure S9-1. ^{1}H NMR spectrum (600 MHz, DMSO-d_6) of compound 3i

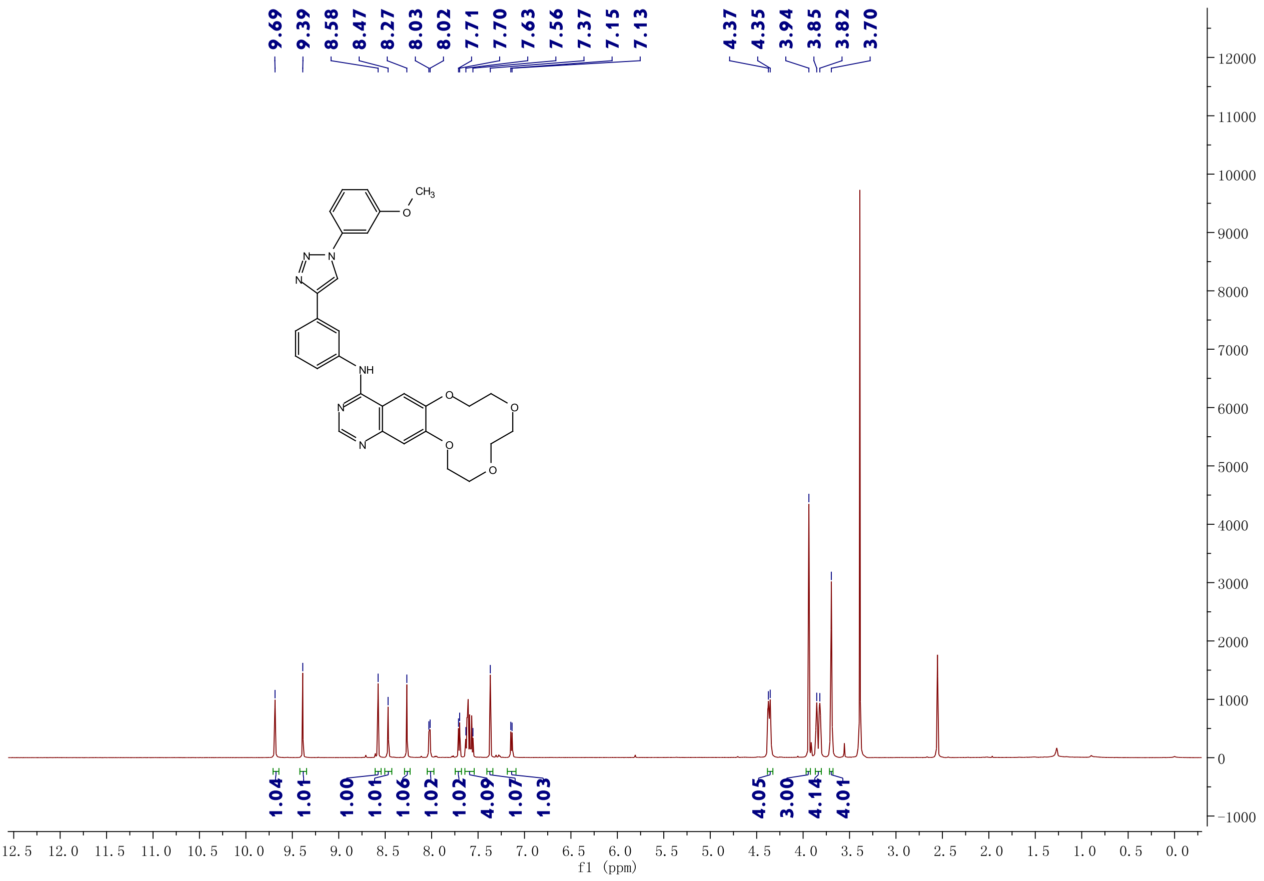

Figure S9-2. ^{13}C NMR spectrum (150 MHz, DMSO-d_6) of compound 3i

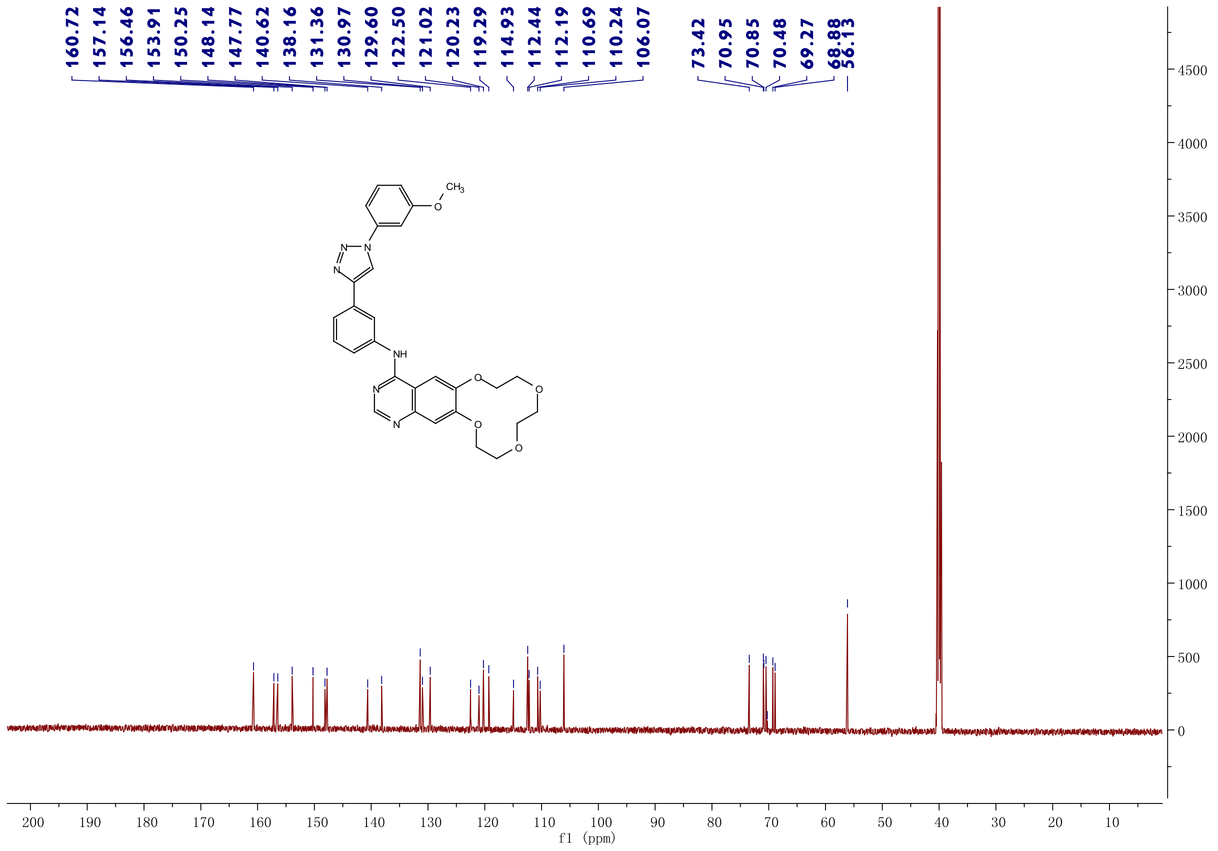

Figure S9-3. HR MS of compound 3i

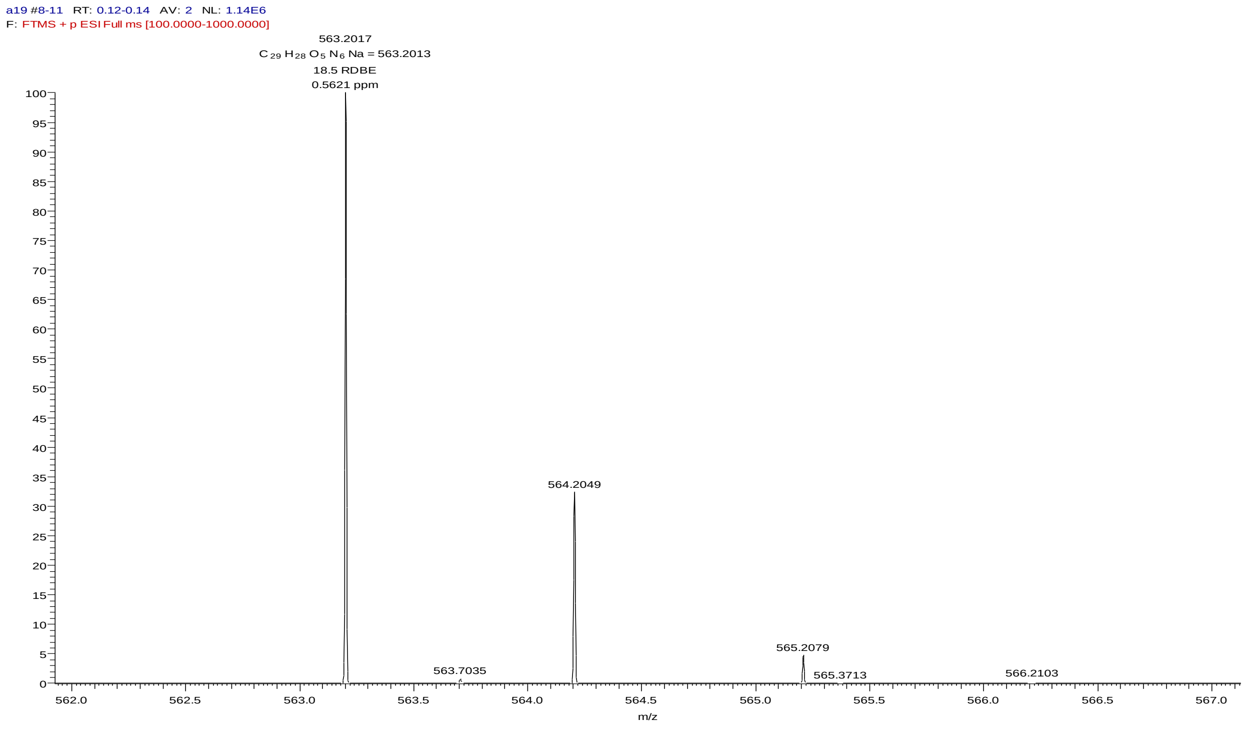

Figure S10-1. ^{1}H NMR spectrum (600 MHz, DMSO-d$_6$) of compound 3j

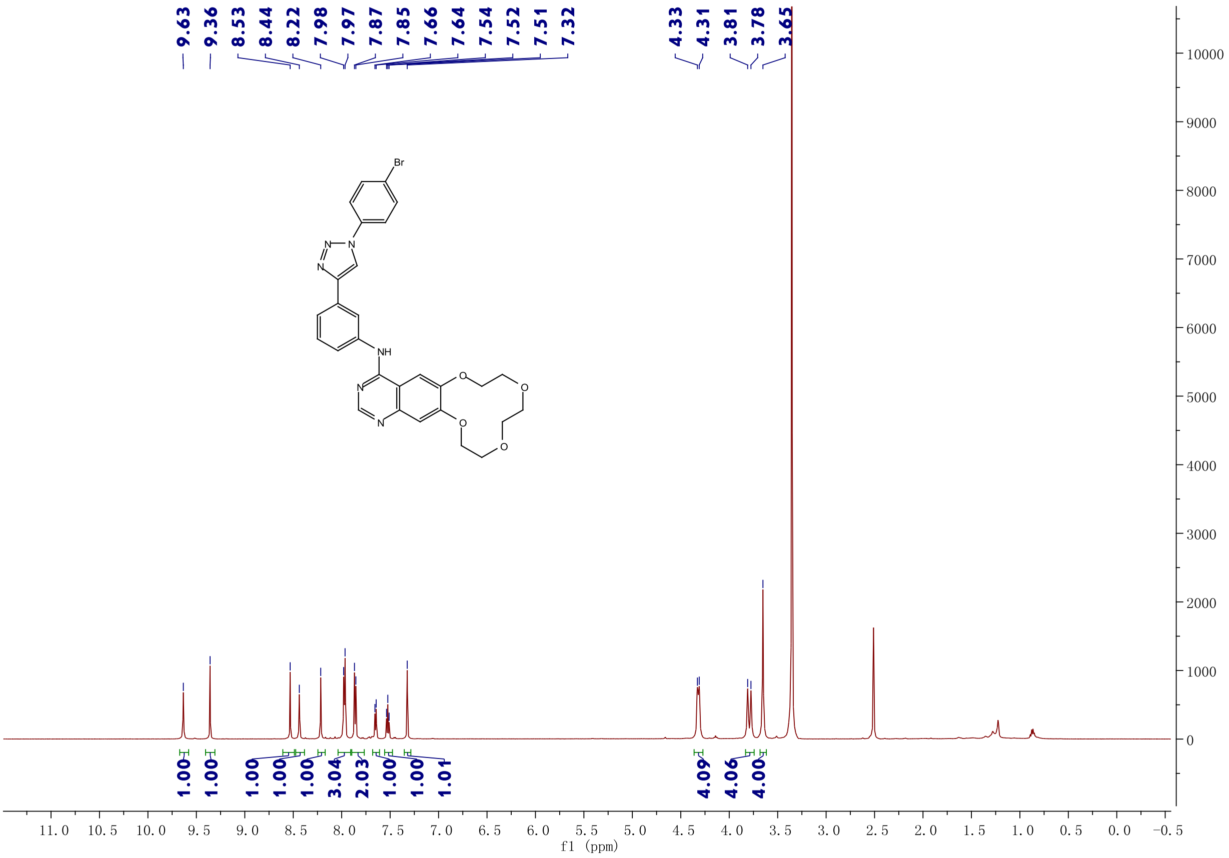

Figure S10-2. ^{13}C NMR spectrum (150 MHz, DMSO-d_6) of compound 3j

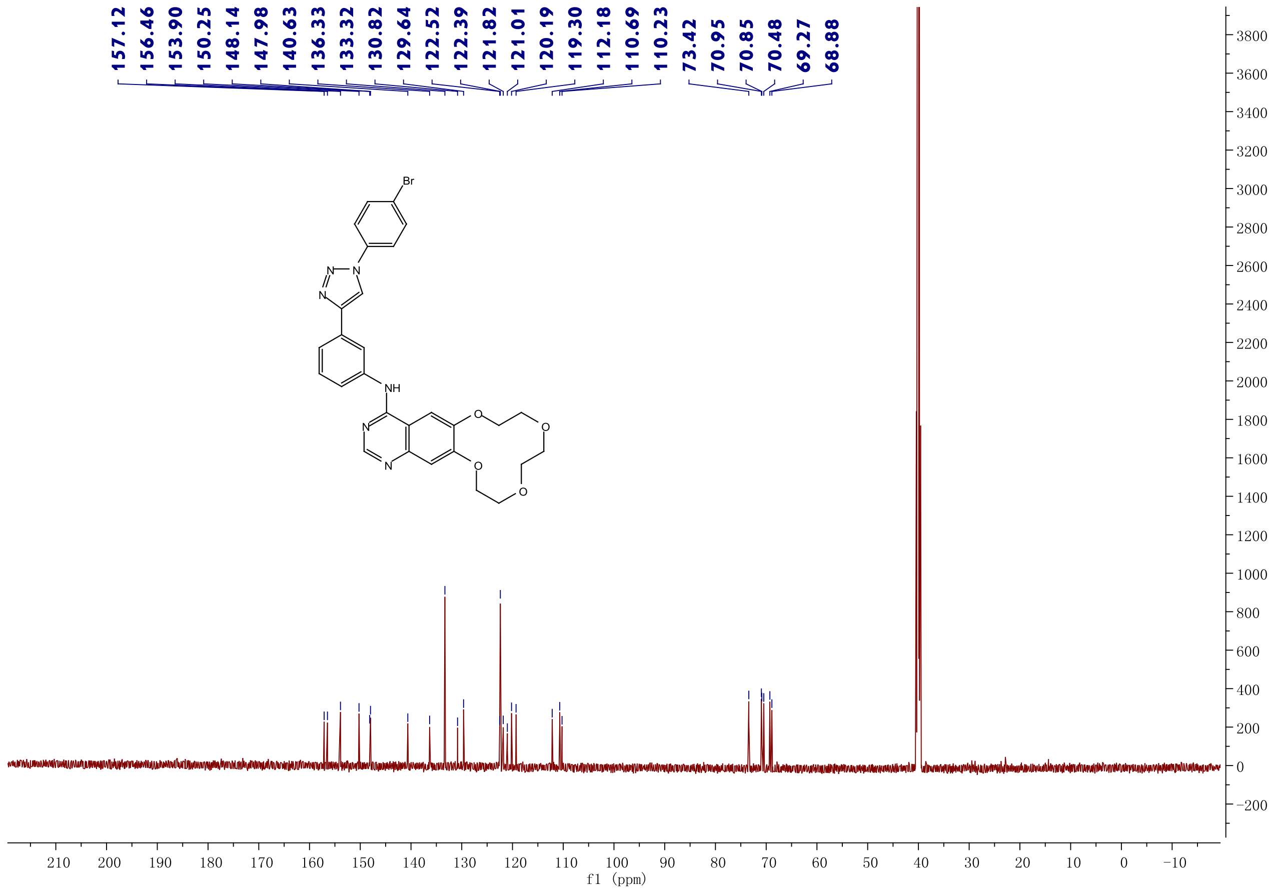

Figure S10-3. HR MS of compound 3j

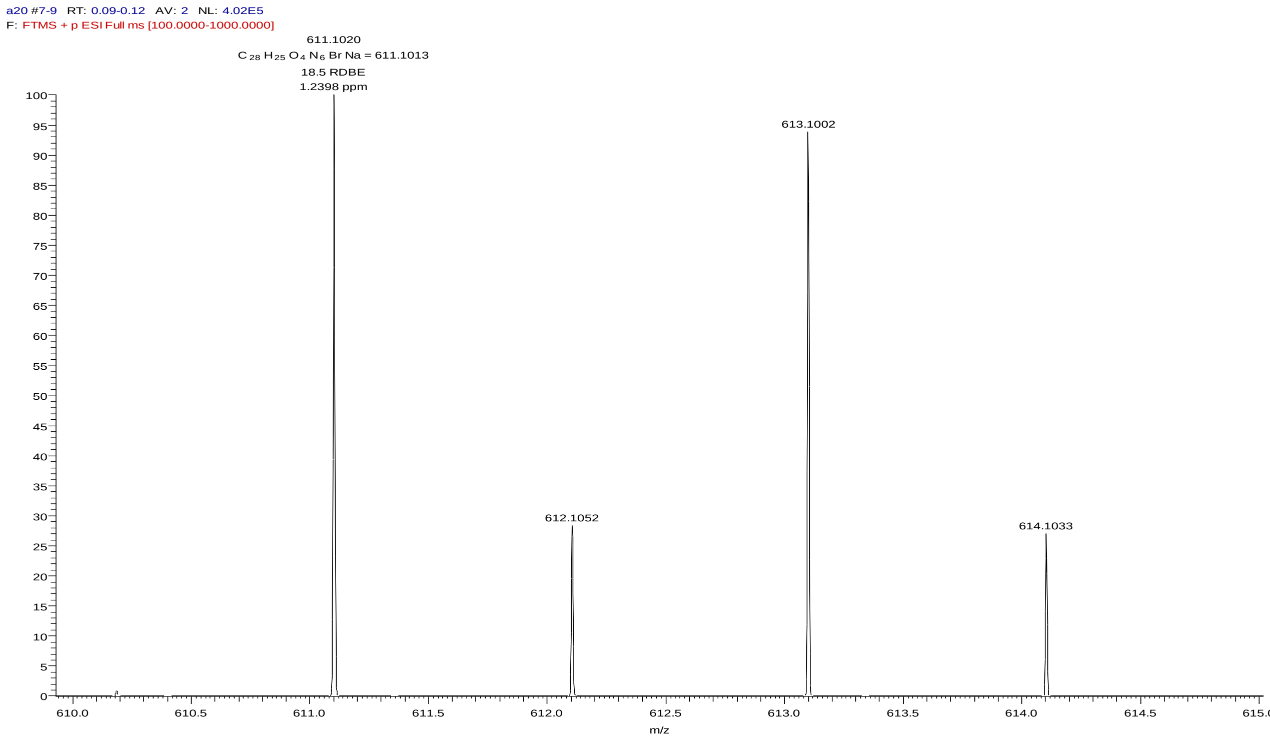

Figure S11-1. ^{1}H NMR spectrum (600 MHz, DMSO-d_6) of compound 3k

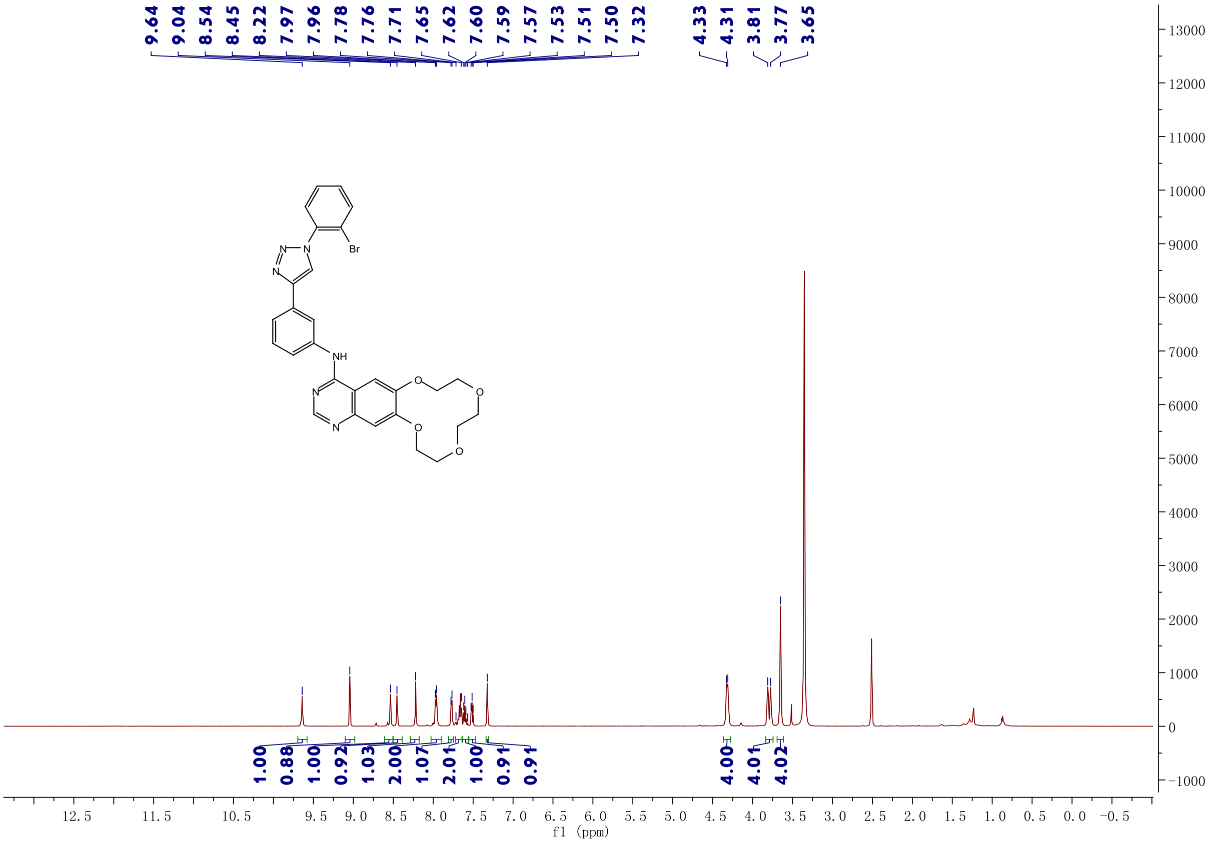

Figure S11-2. ^{13}C NMR spectrum (150 MHz, DMSO-d_6) of compound 3k

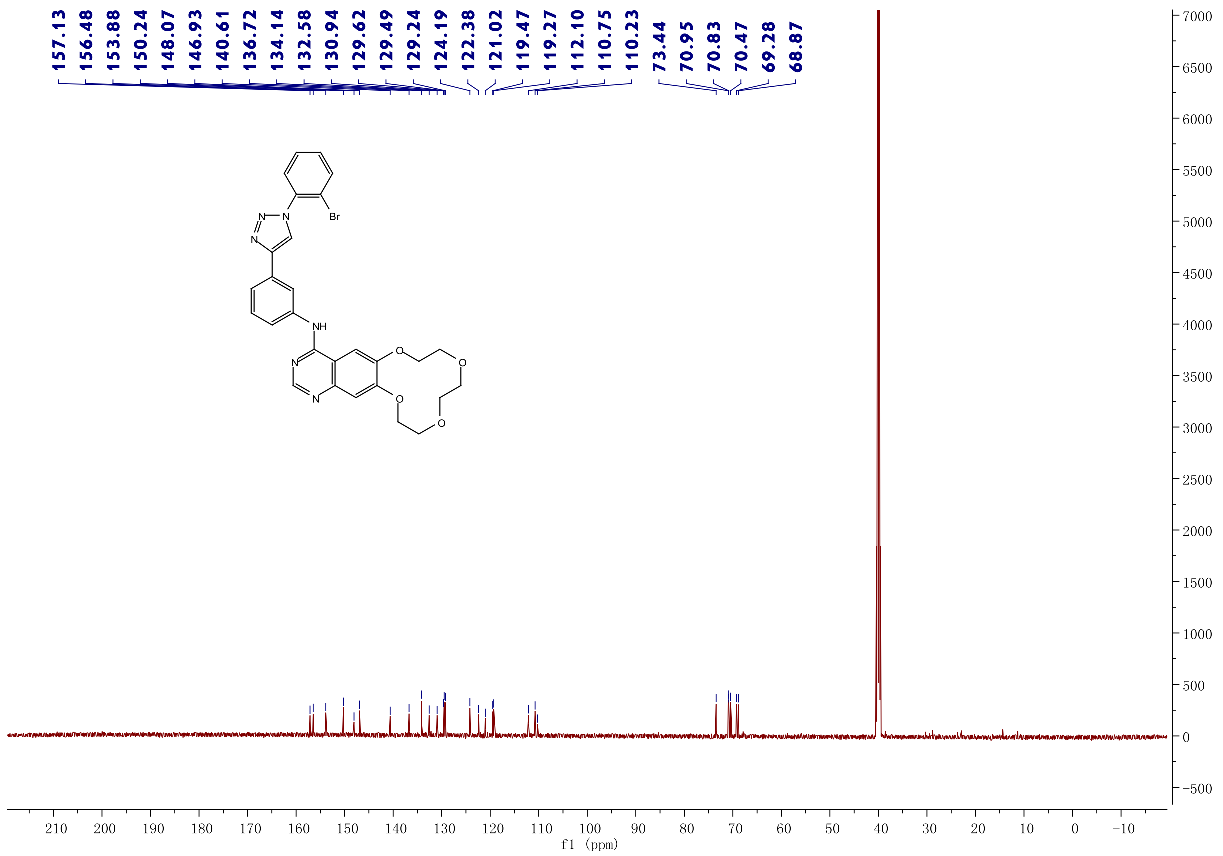

Figure S11-3. HR MS of compound 3k

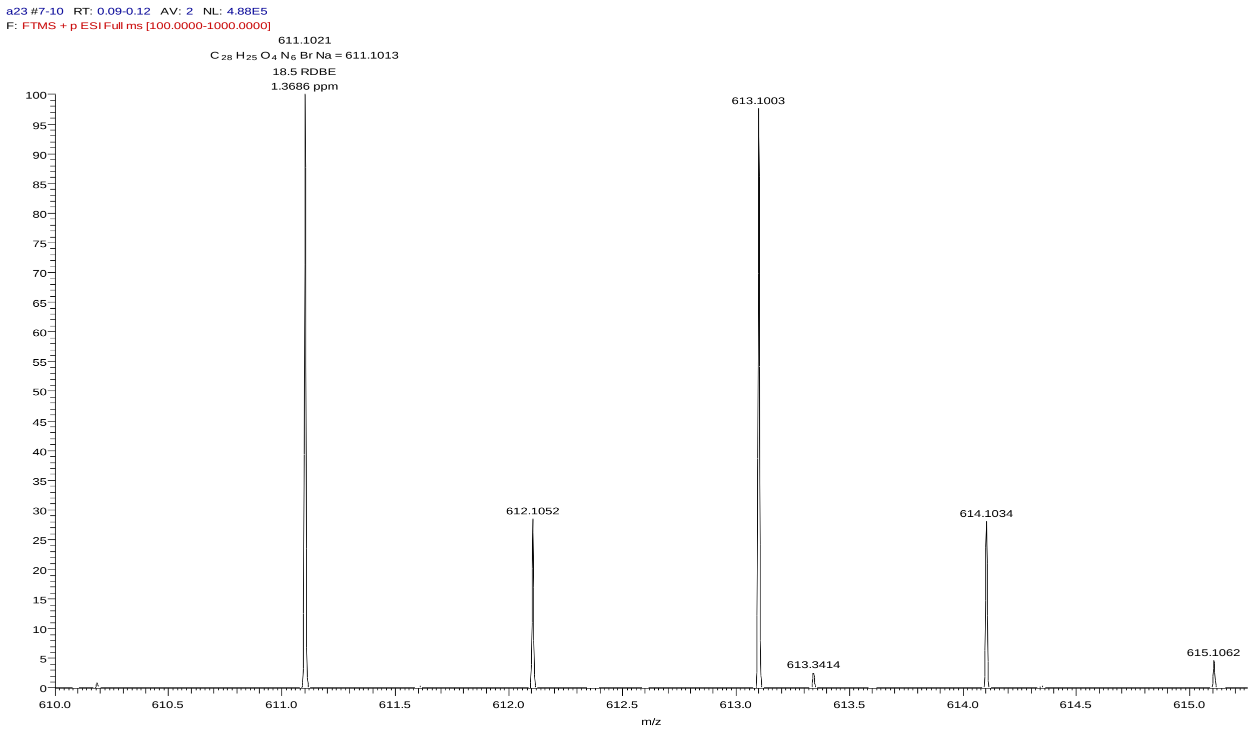

Figure S12-1. ^{1}H NMR spectrum (600 MHz, DMSO-d$_6$) of compound 3l

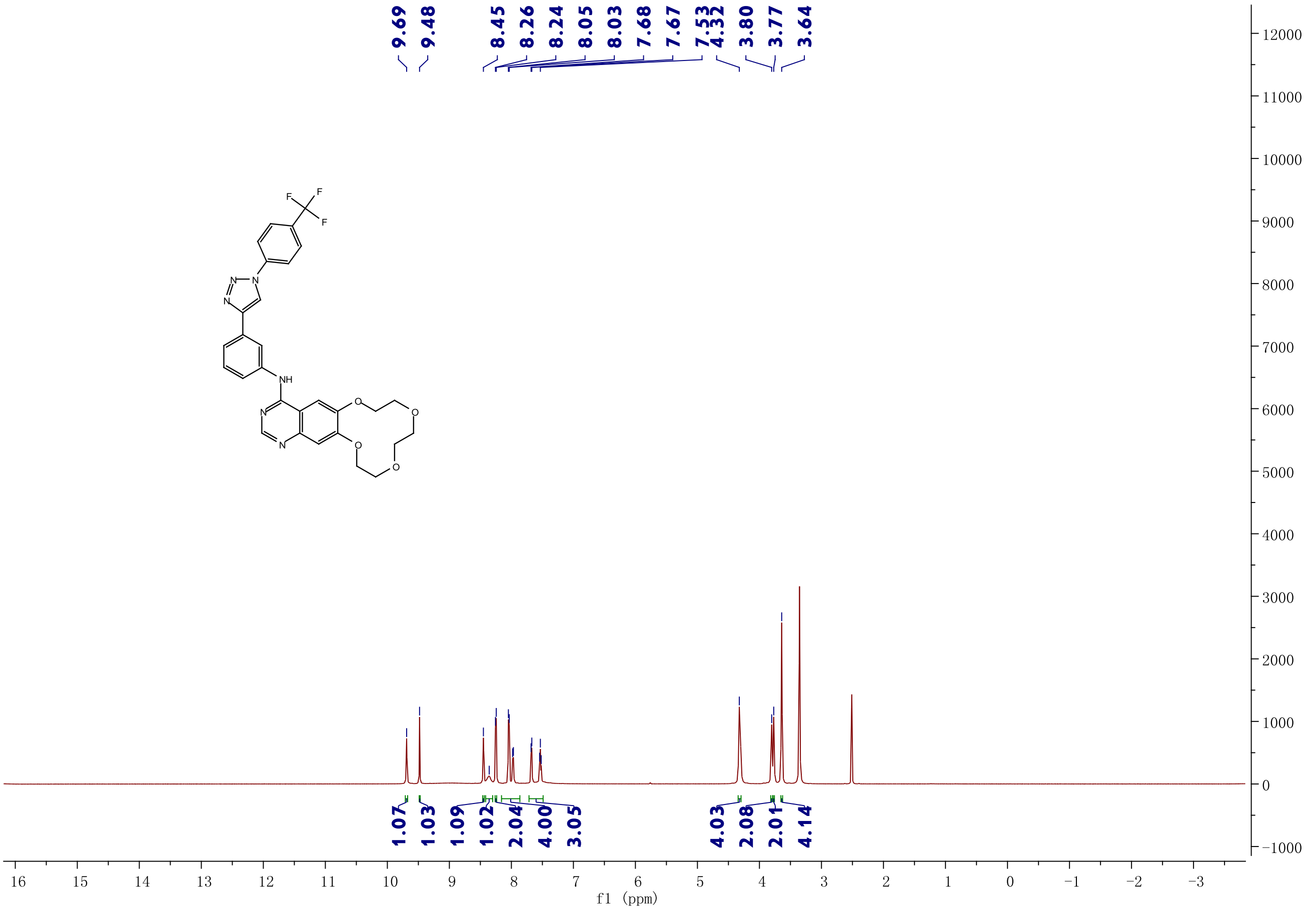

Figure S12-2. ^{12}C NMR spectrum (150 MHz, DMSO-d_6) of compound 3l

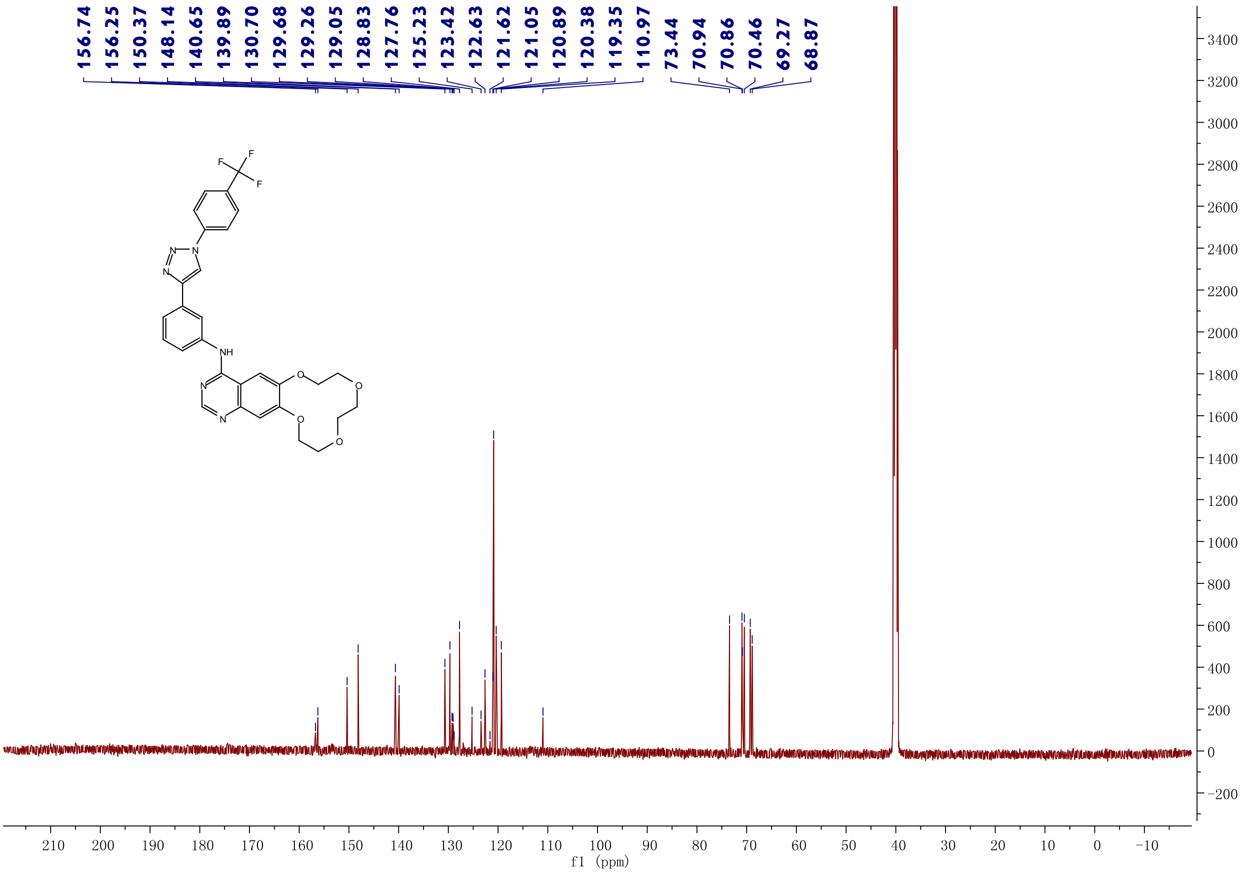

Figure S12-3. HR MS of compound 3l

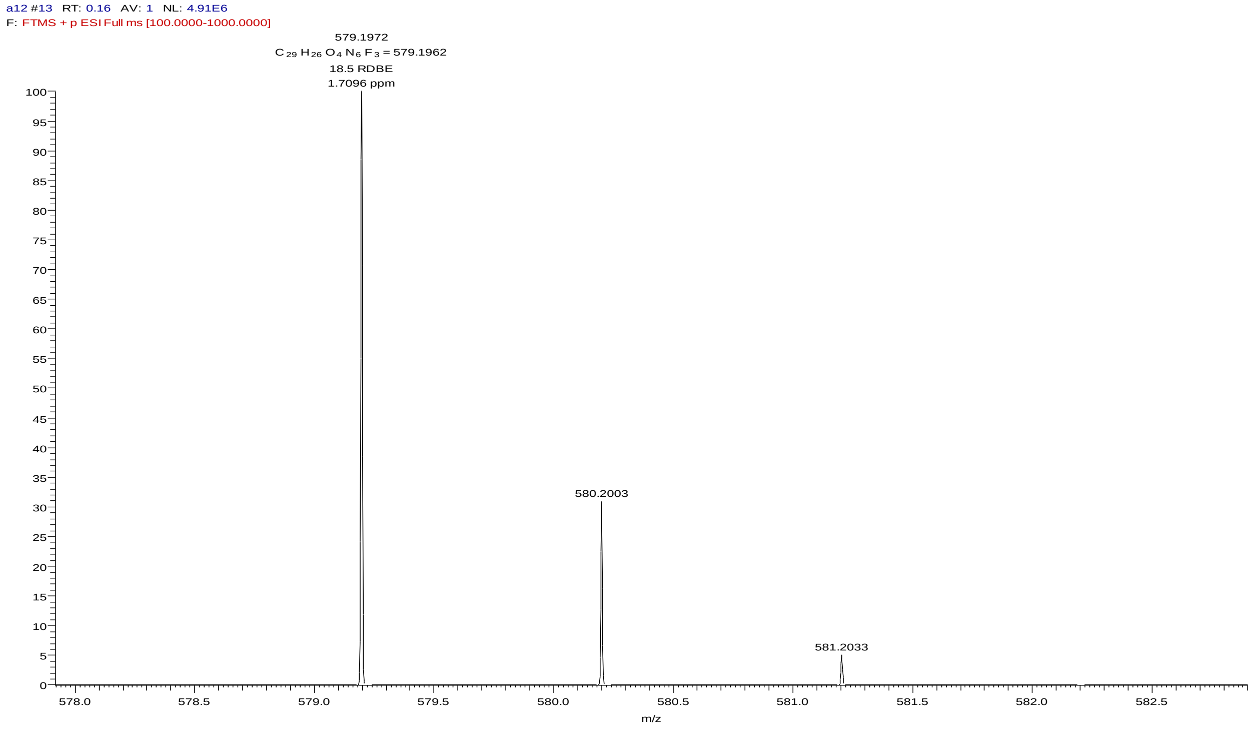

Figure S13-1. ^{1}H NMR spectrum (600 MHz, DMSO-d_6) of compound 3m

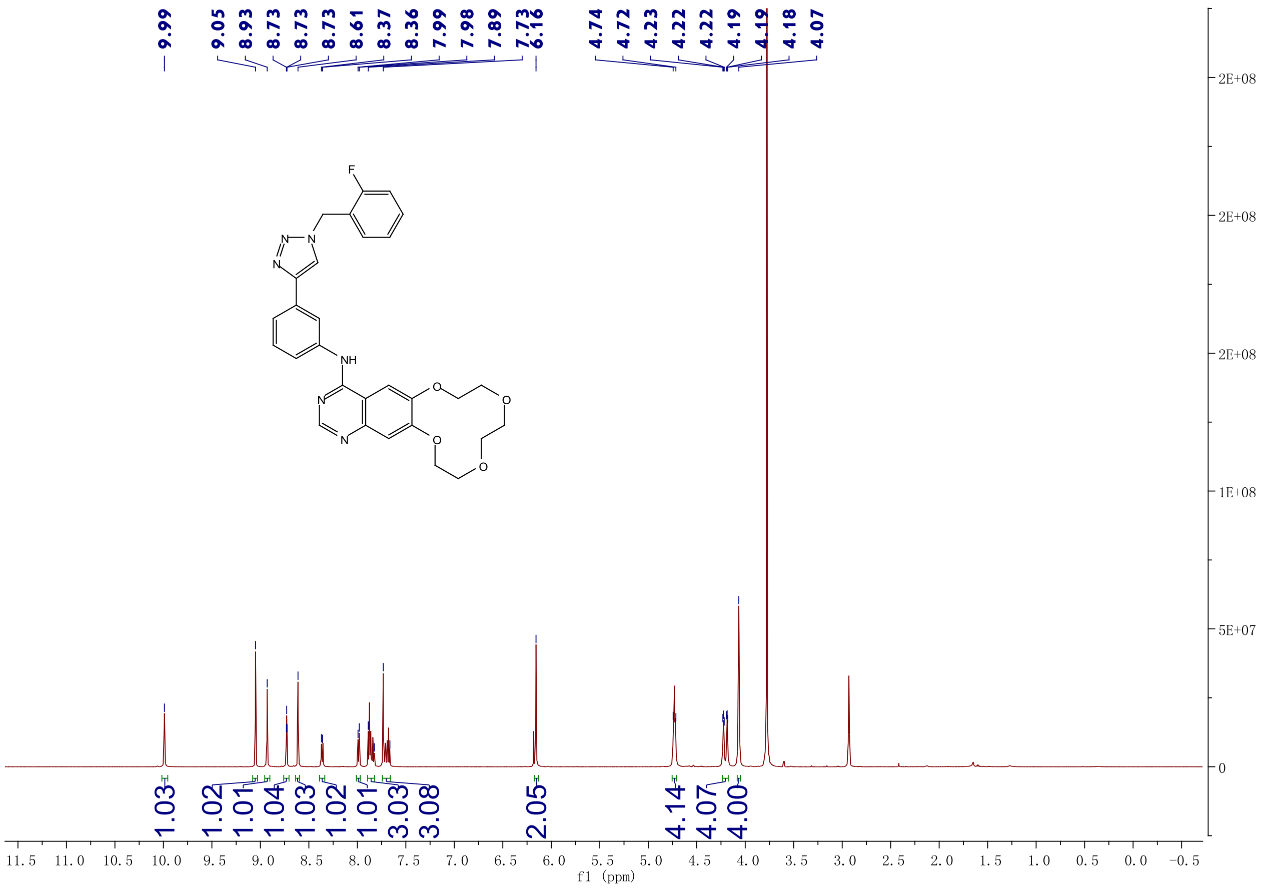

Figure S13-2. ^{13}C NMR spectrum (150 MHz, DMSO-d$_6$) of compound 3m

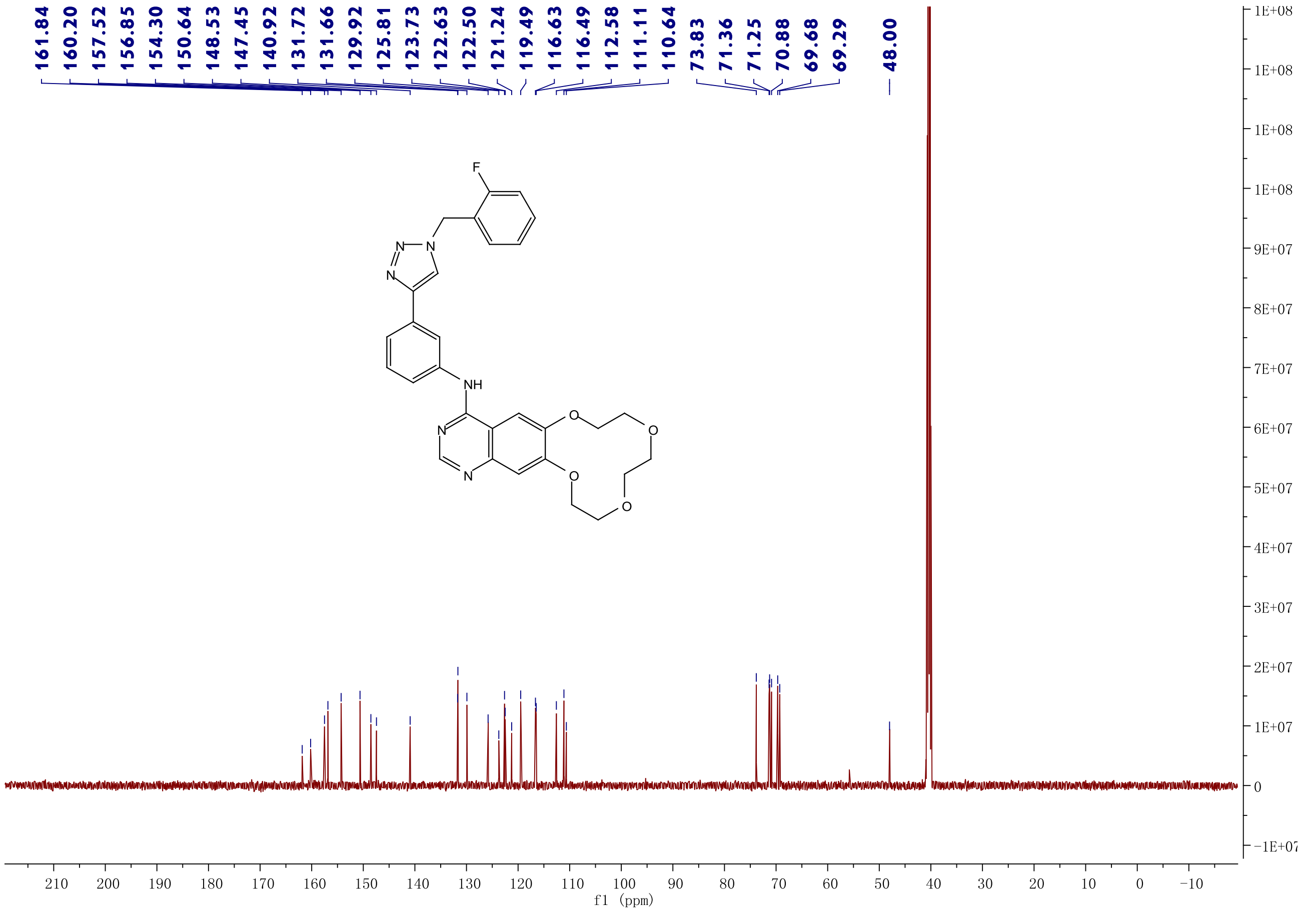

Figure S13-3. HR MS of compound 3m

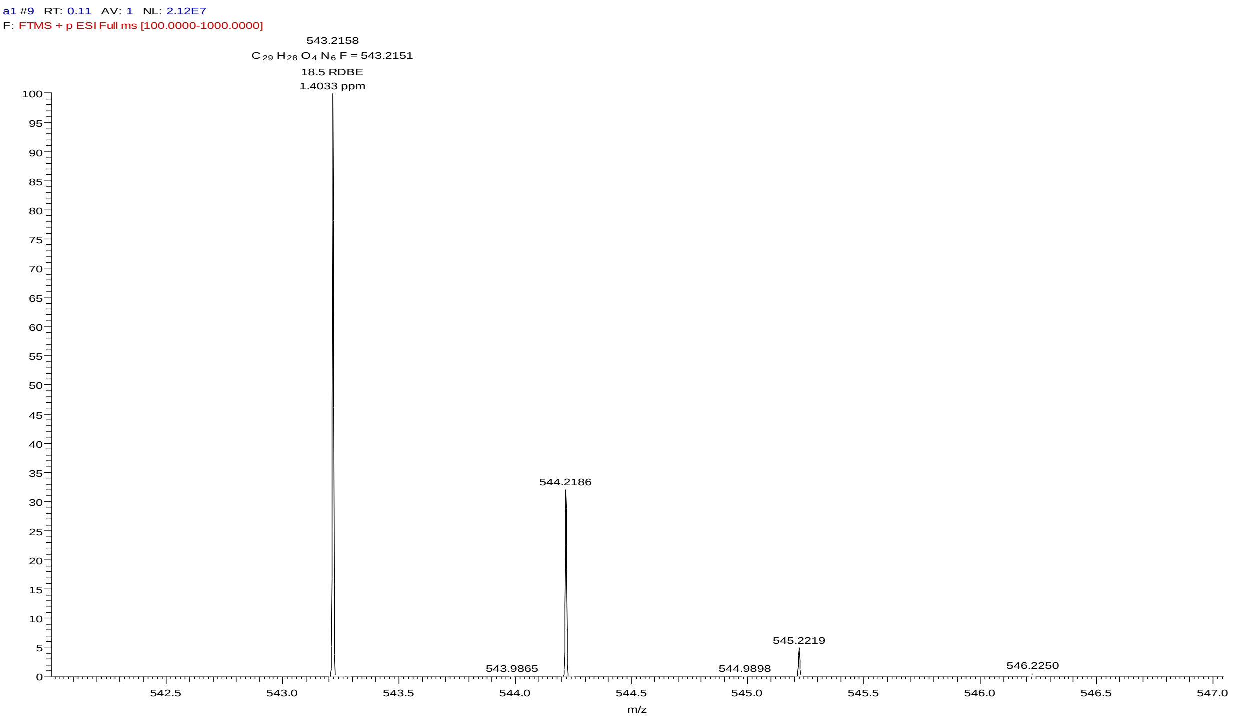

Figure S14-1. ^{1}H NMR spectrum (600 MHz, DMSO-d_6) of compound 3n

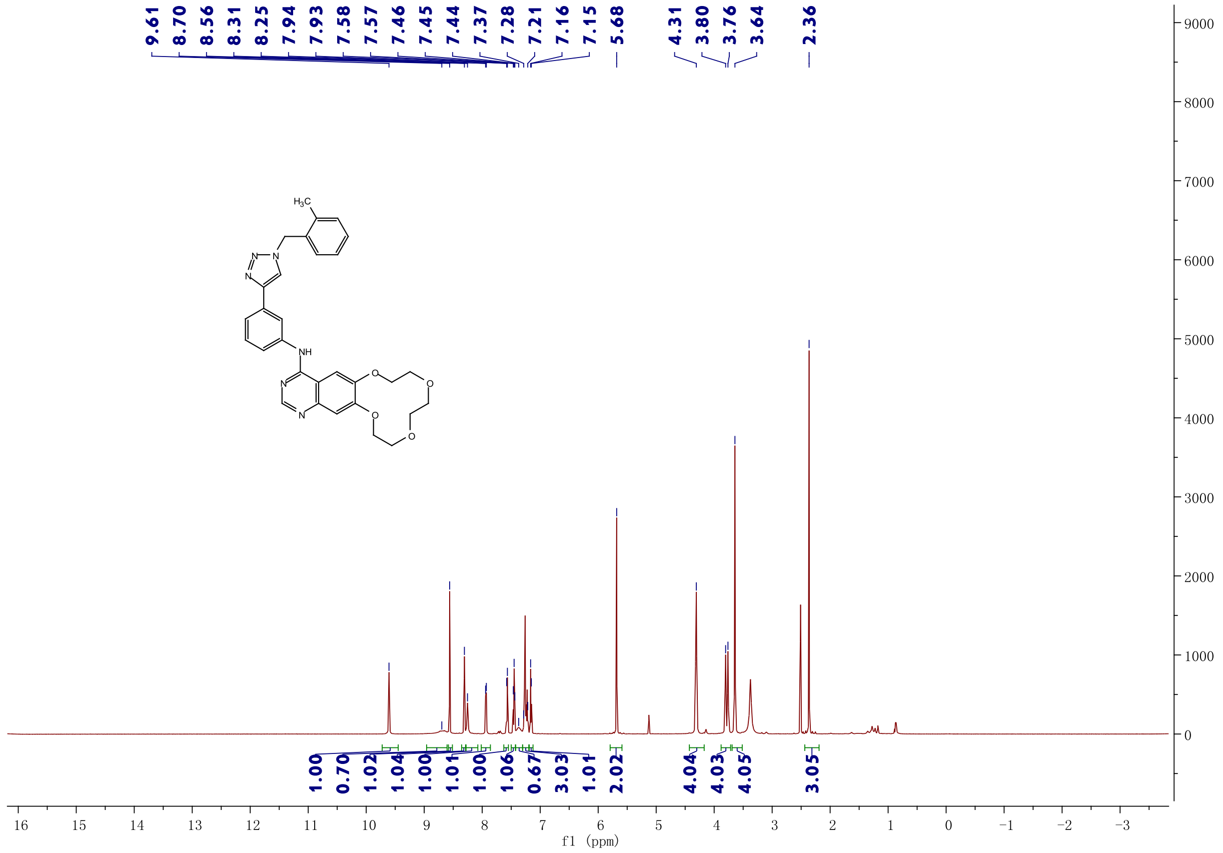

Figure S14-2. ^{13}C NMR spectrum (150 MHz, DMSO-d_6) of compound 3n

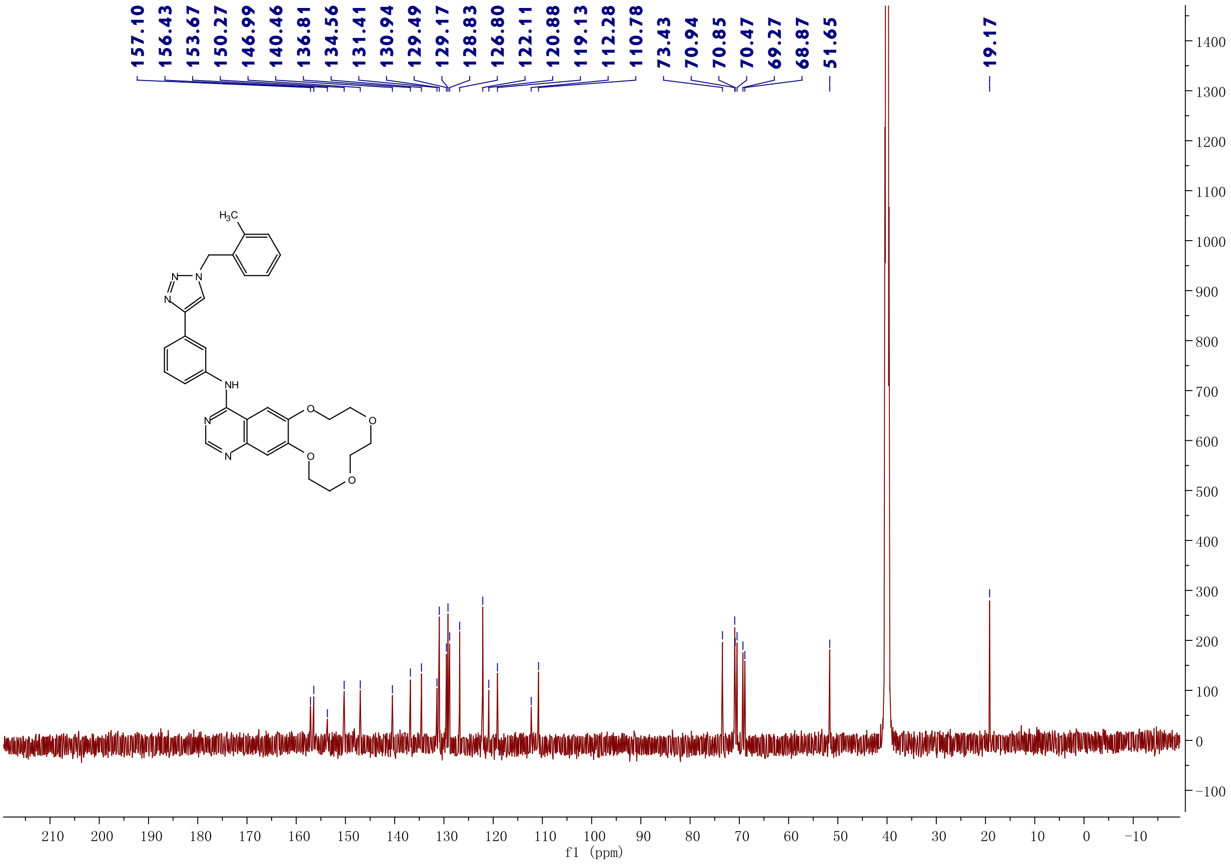

Figure S14-3. HR MS of compound 3n

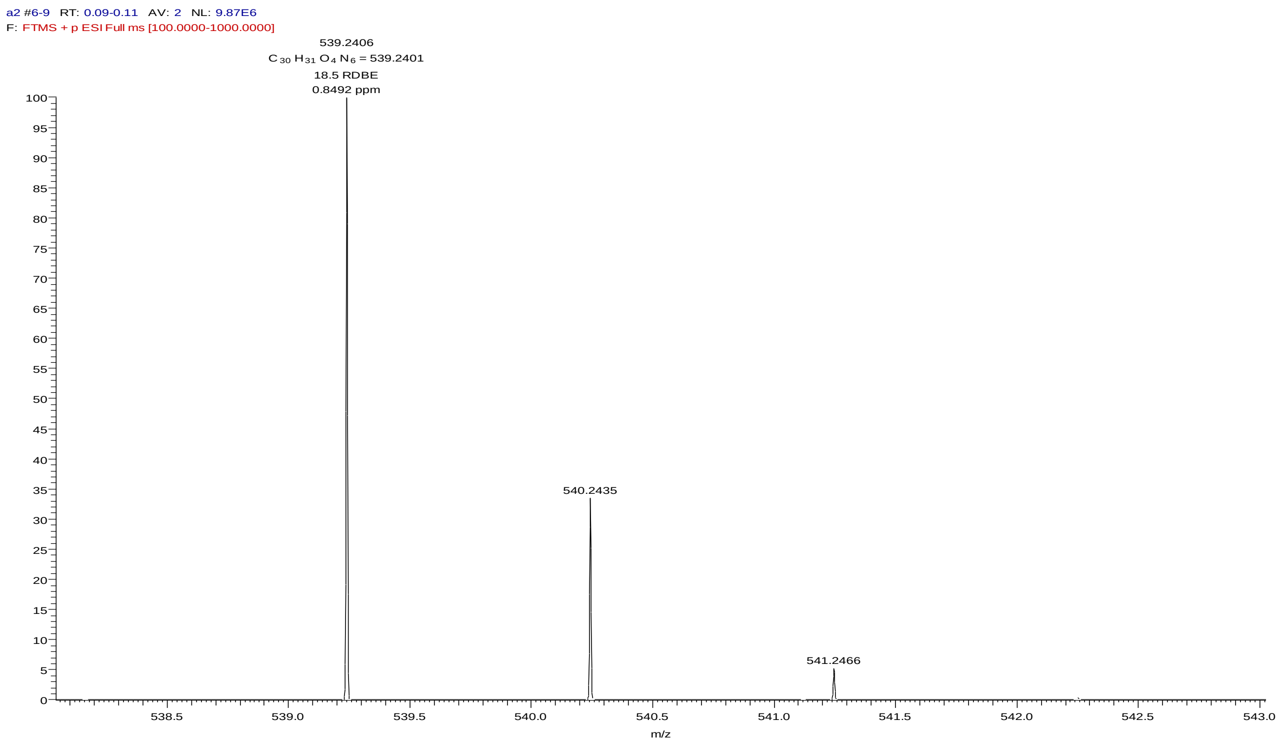

Figure S15-1. ^{1}H NMR spectrum (600 MHz, DMSO-d_6) of compound 3o

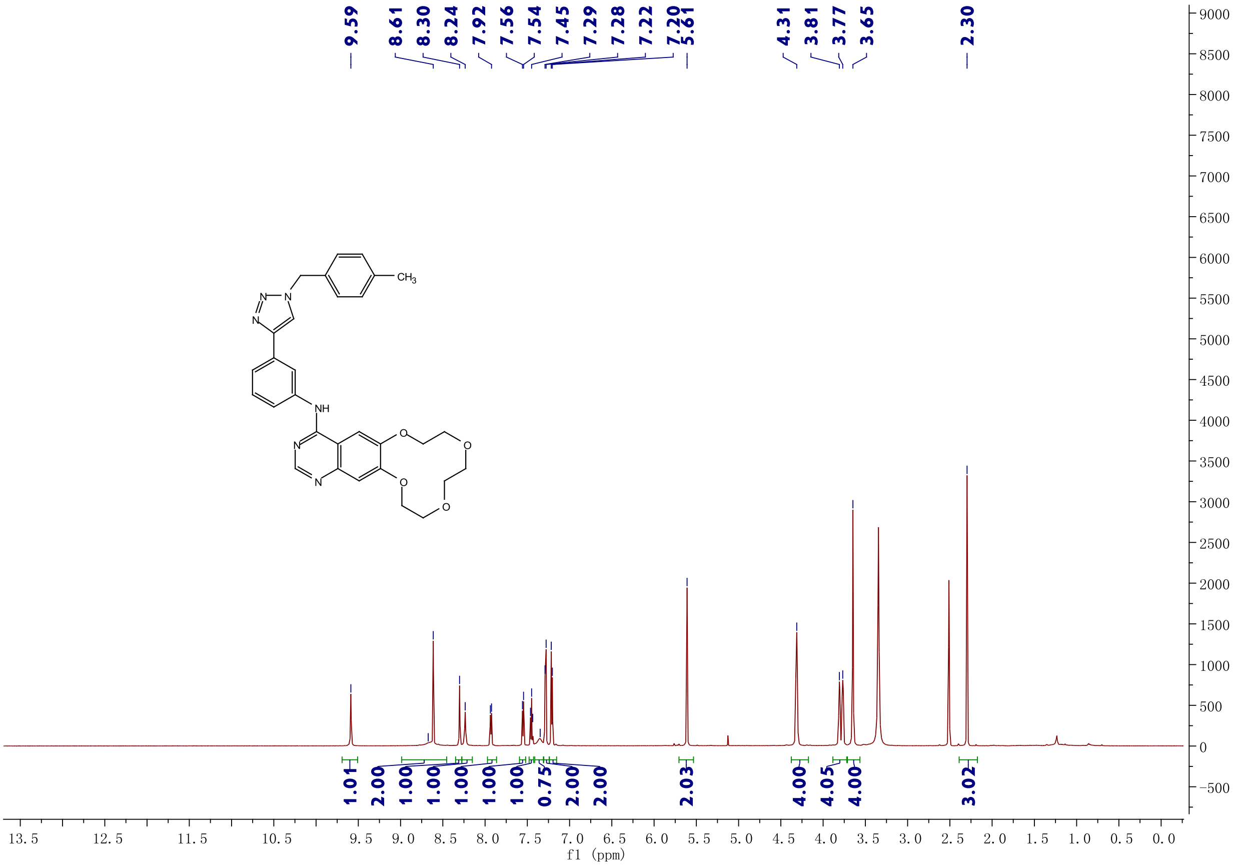

Figure S15-2. ^{13}C NMR spectrum (150 MHz, DMSO-d_6) of compound 3o

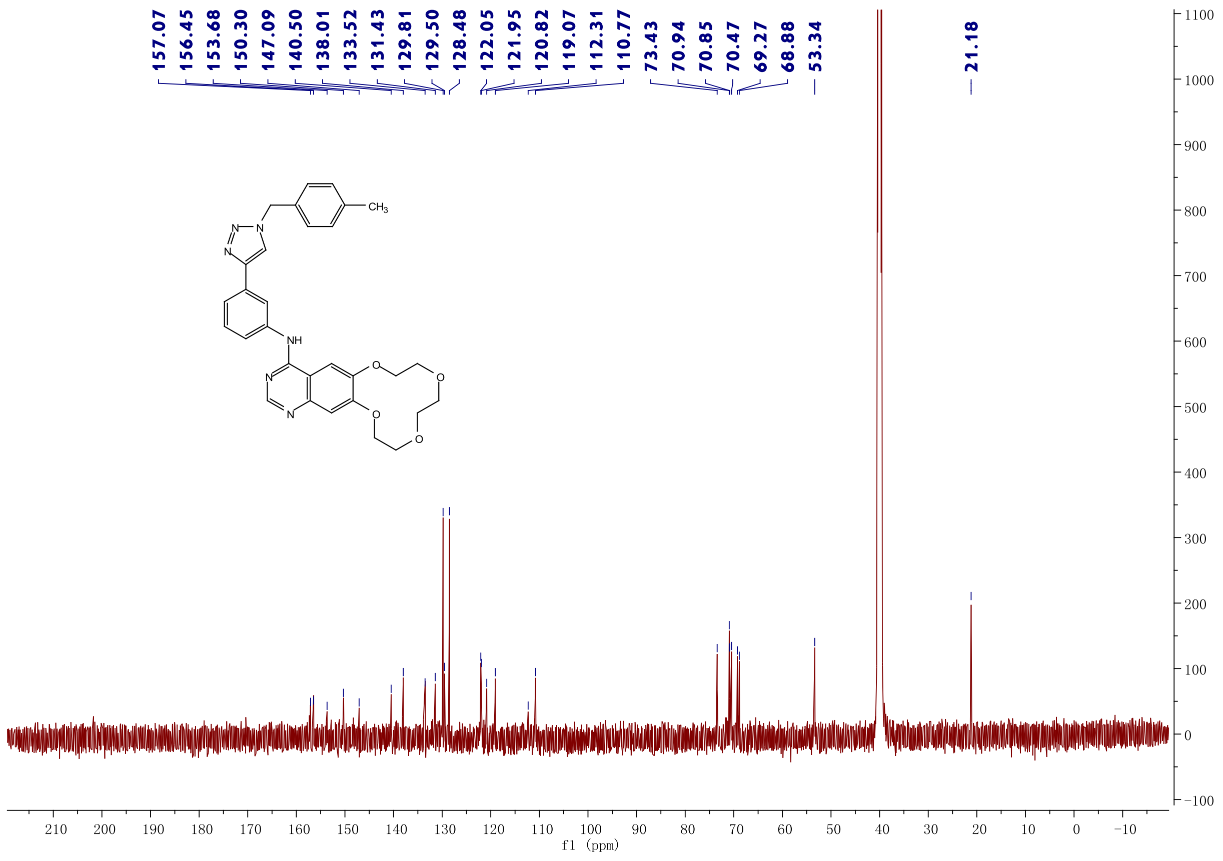

Figure S15-3. HR MS of compound 3o

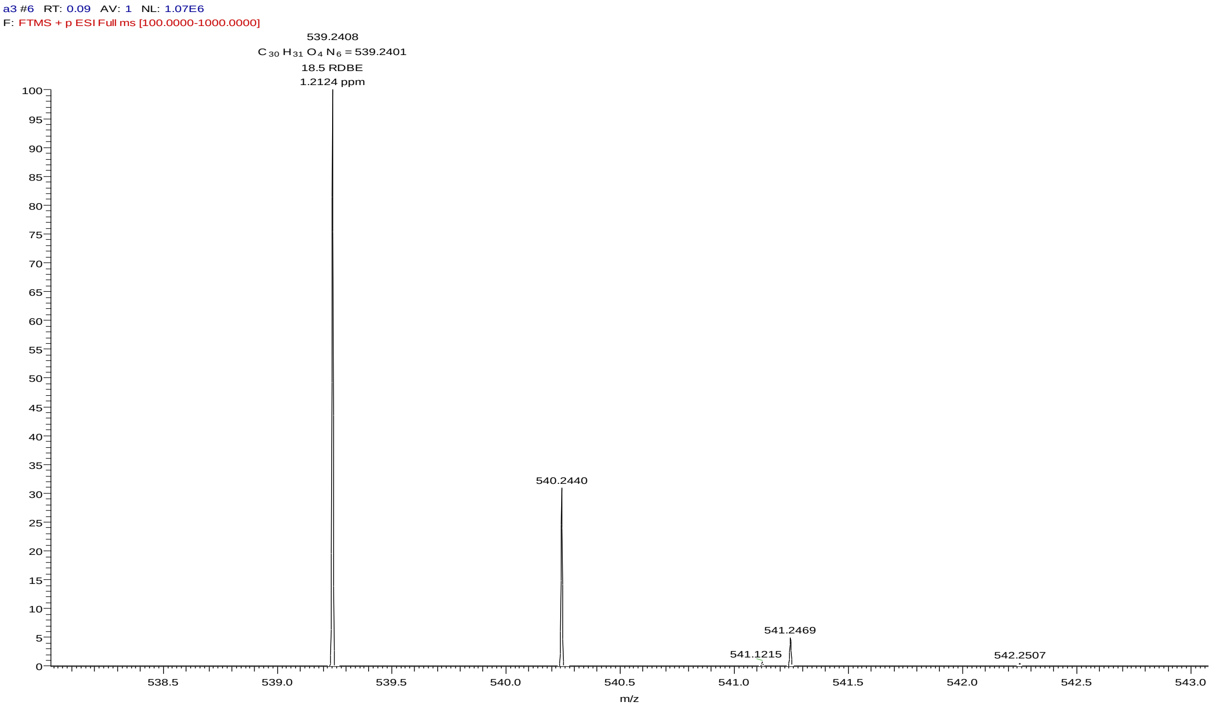

Figure S16-1. ^{1}H NMR spectrum (600 MHz, DMSO-d_6) of compound 3p

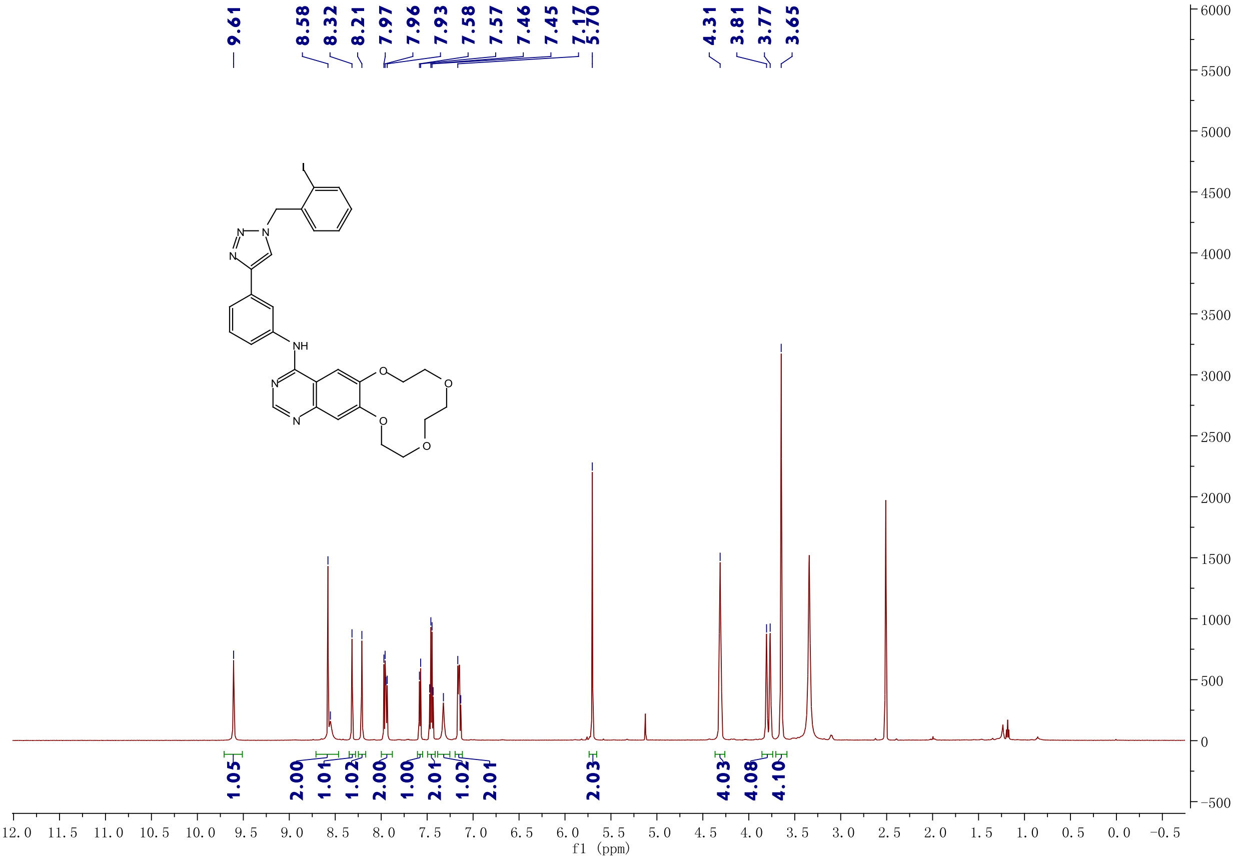

Figure S16-2. ^{13}C NMR spectrum (150 MHz, DMSO-d_6) of compound 3p

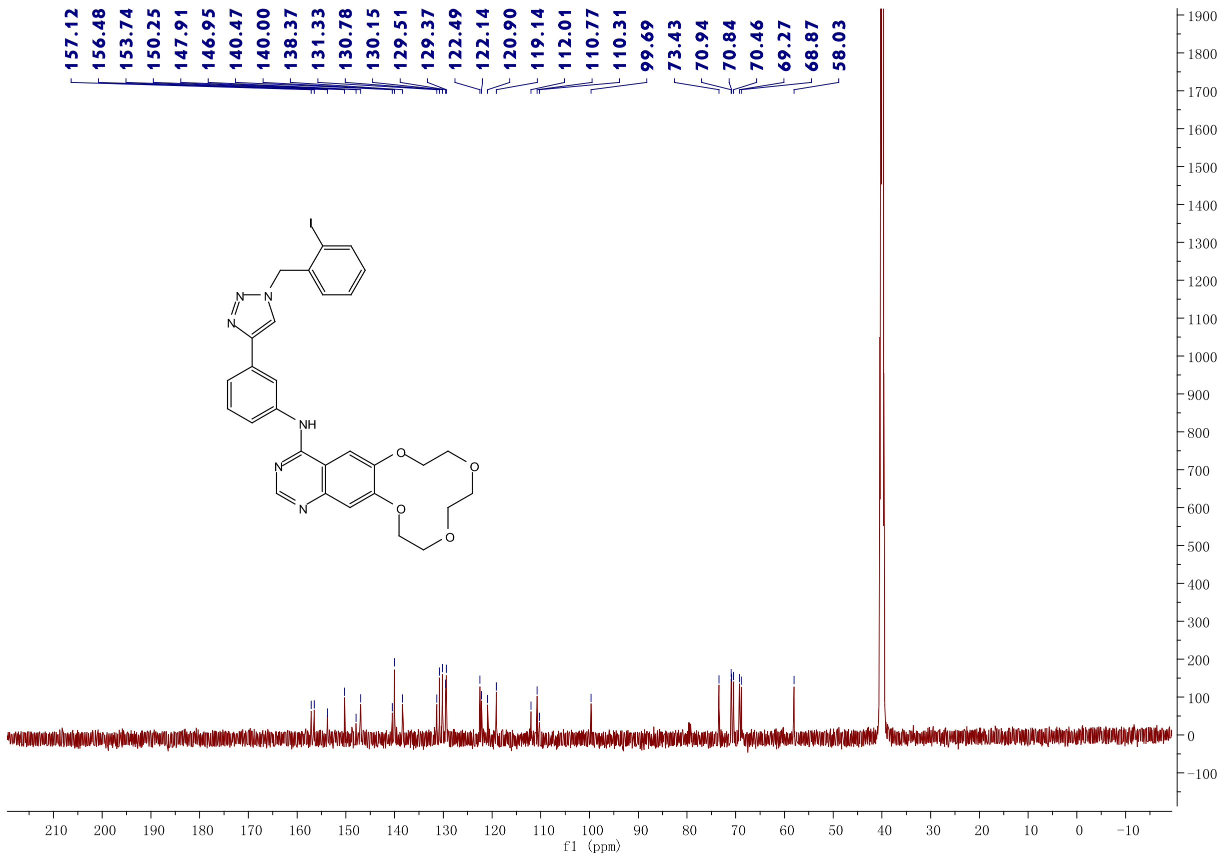

Figure S16-3. HR MS of compound 3p

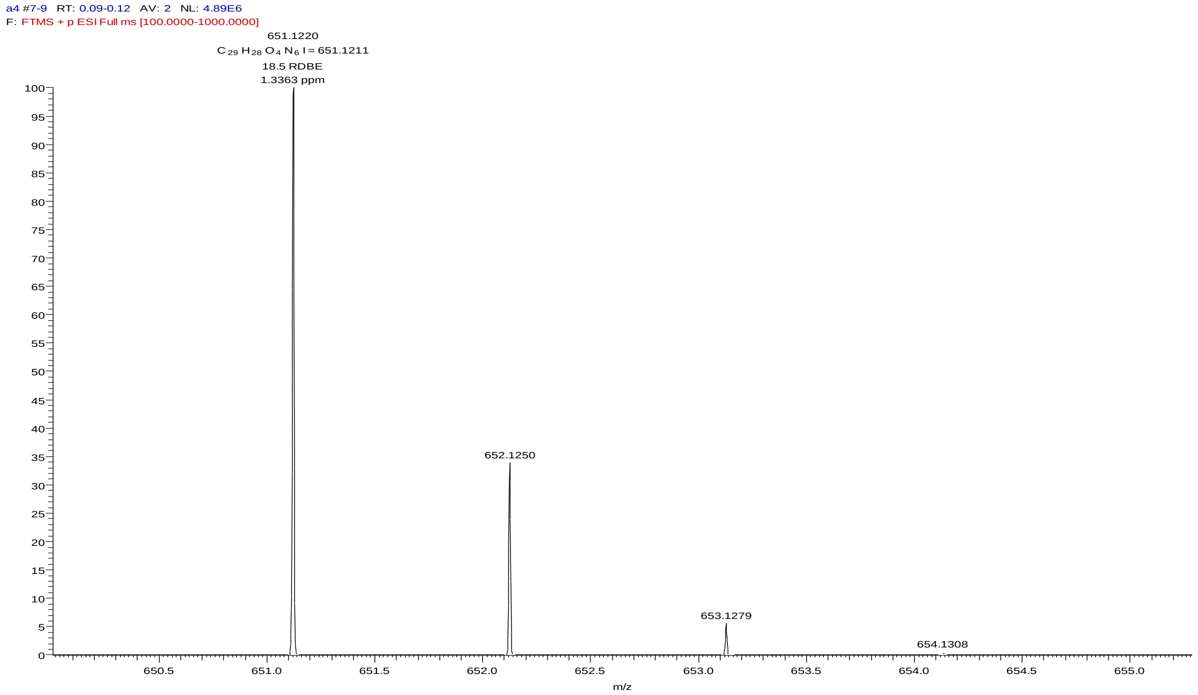

Figure S17-1. ^{1}H NMR spectrum (600 MHz, DMSO-d_6) of compound 3q

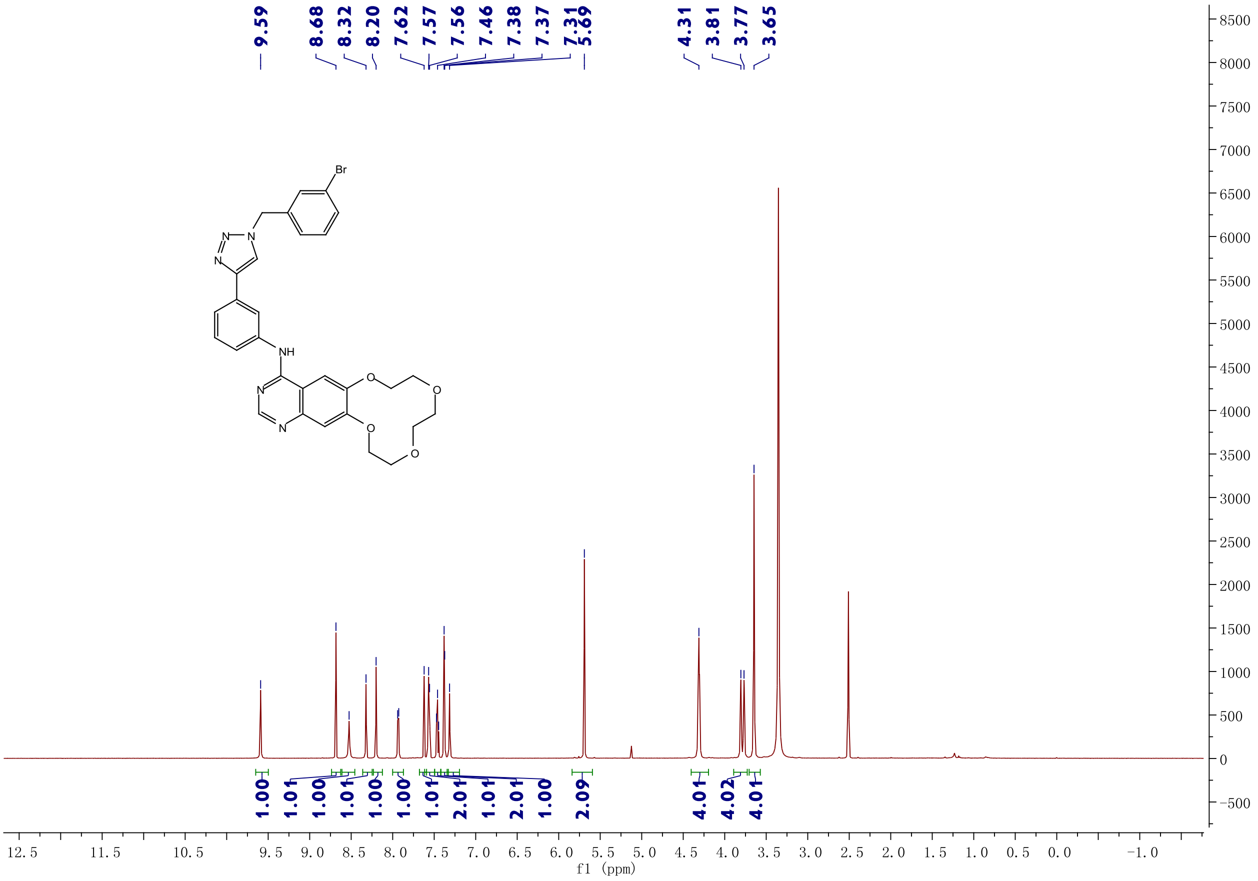

Figure S17-2. ^{13}C NMR spectrum (150 MHz, DMSO-d_6) of compound 3q

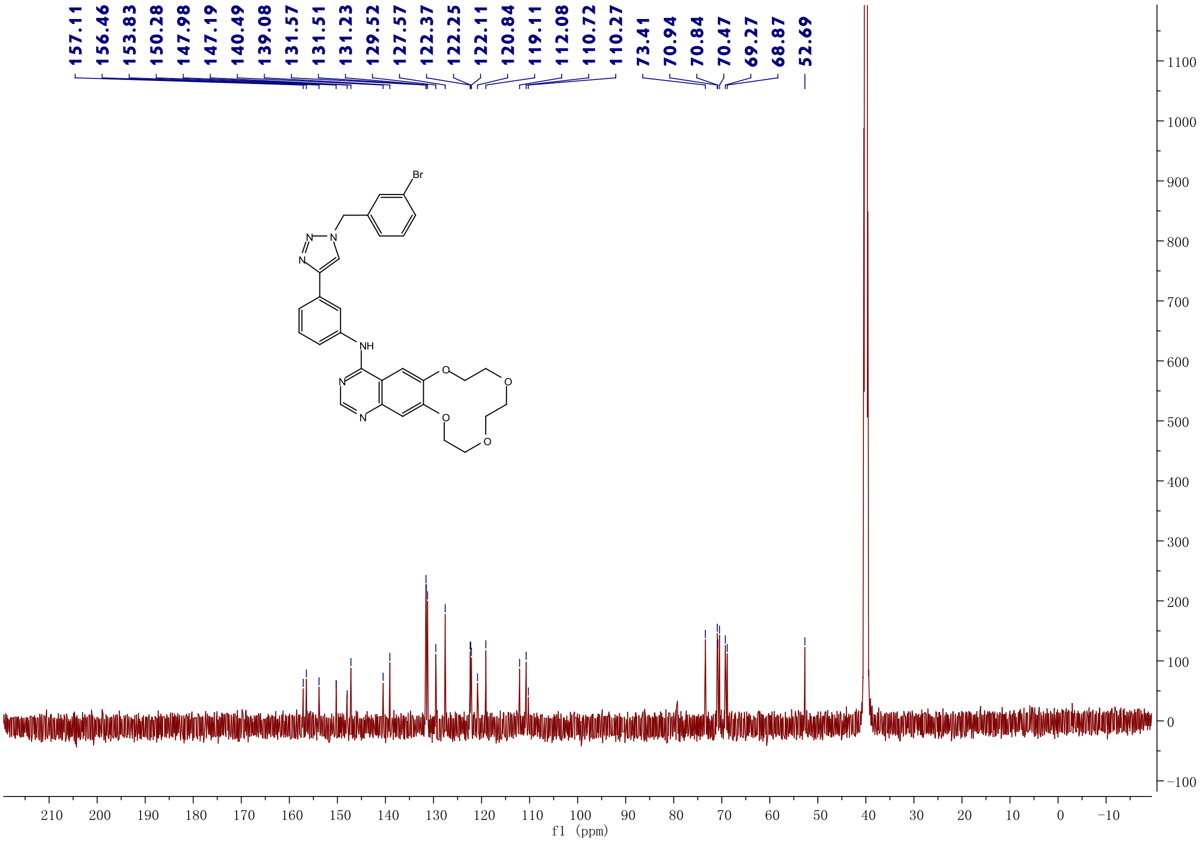

Figure S17-3. HR MS of compound 3q

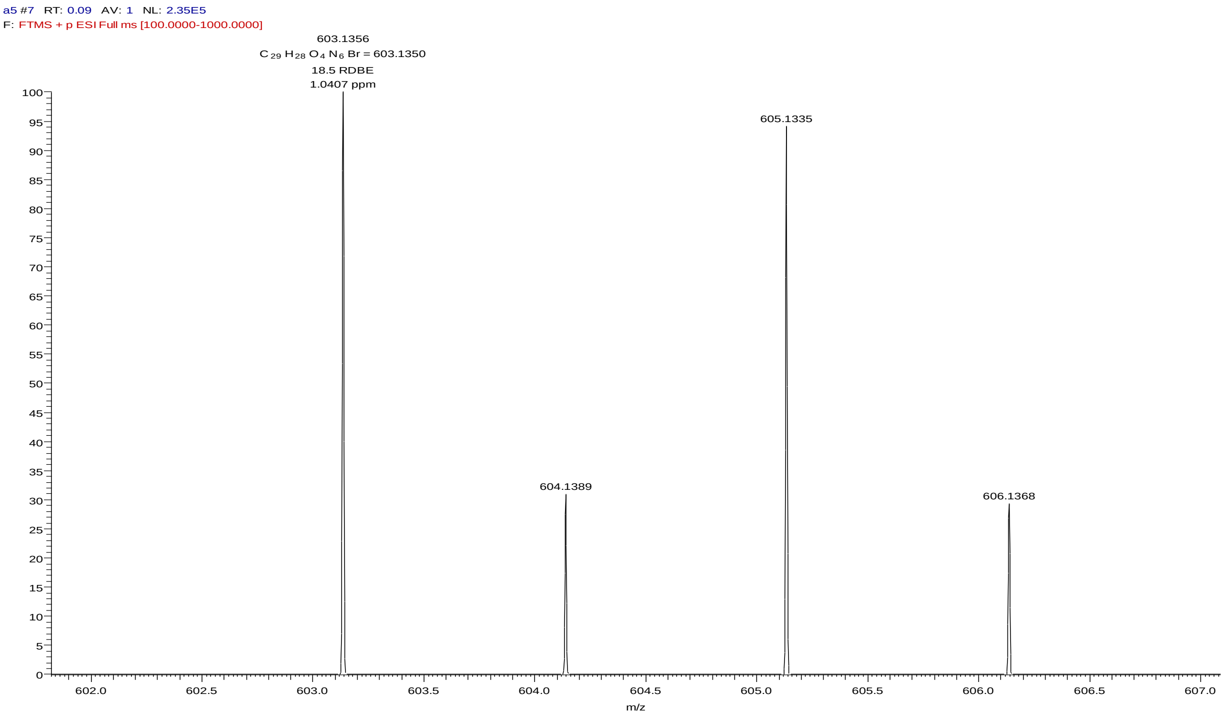

Figure S18-1. ^{1}H NMR spectrum (600 MHz, DMSO-d_6) of compound 3r

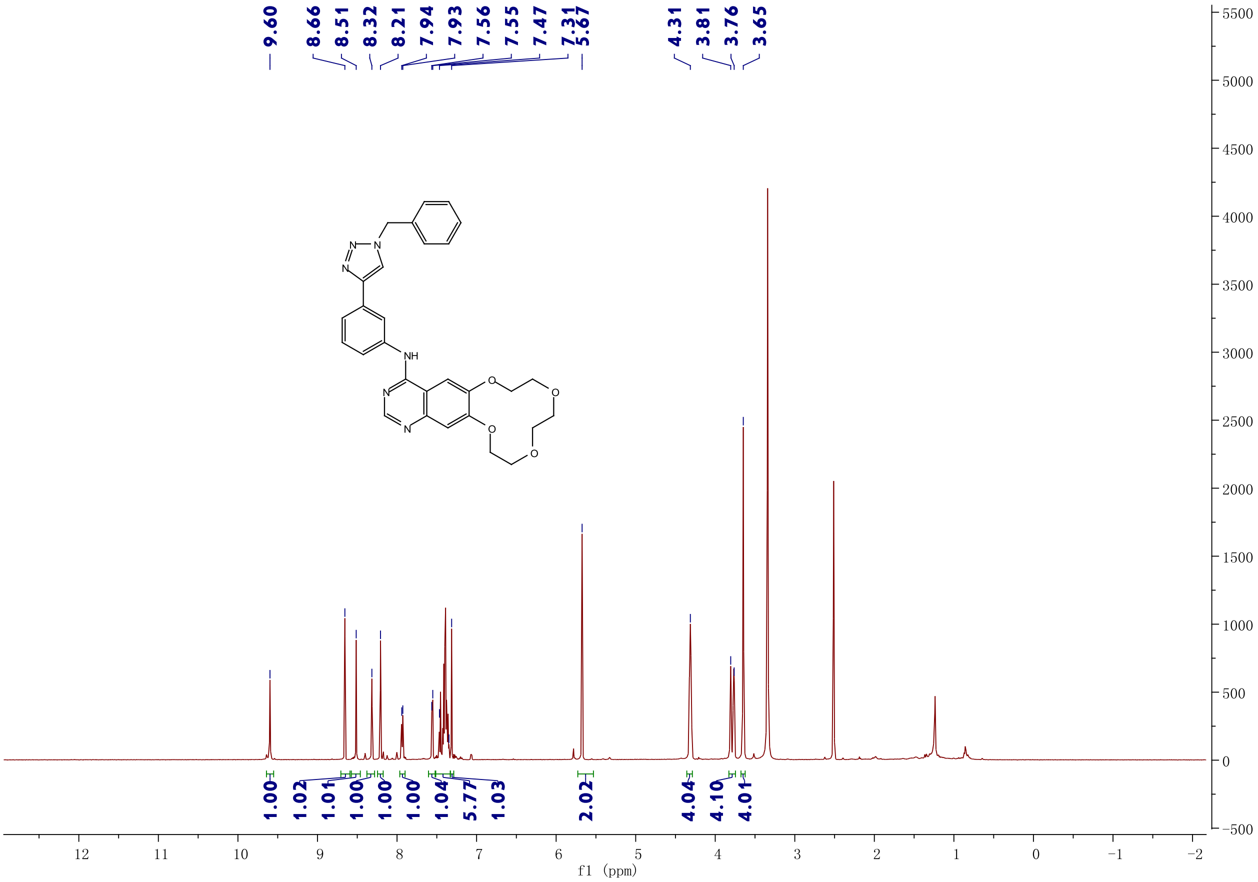

Figure S18-2. ^{13}C NMR spectrum (150 MHz, DMSO-d_6) of compound 3r

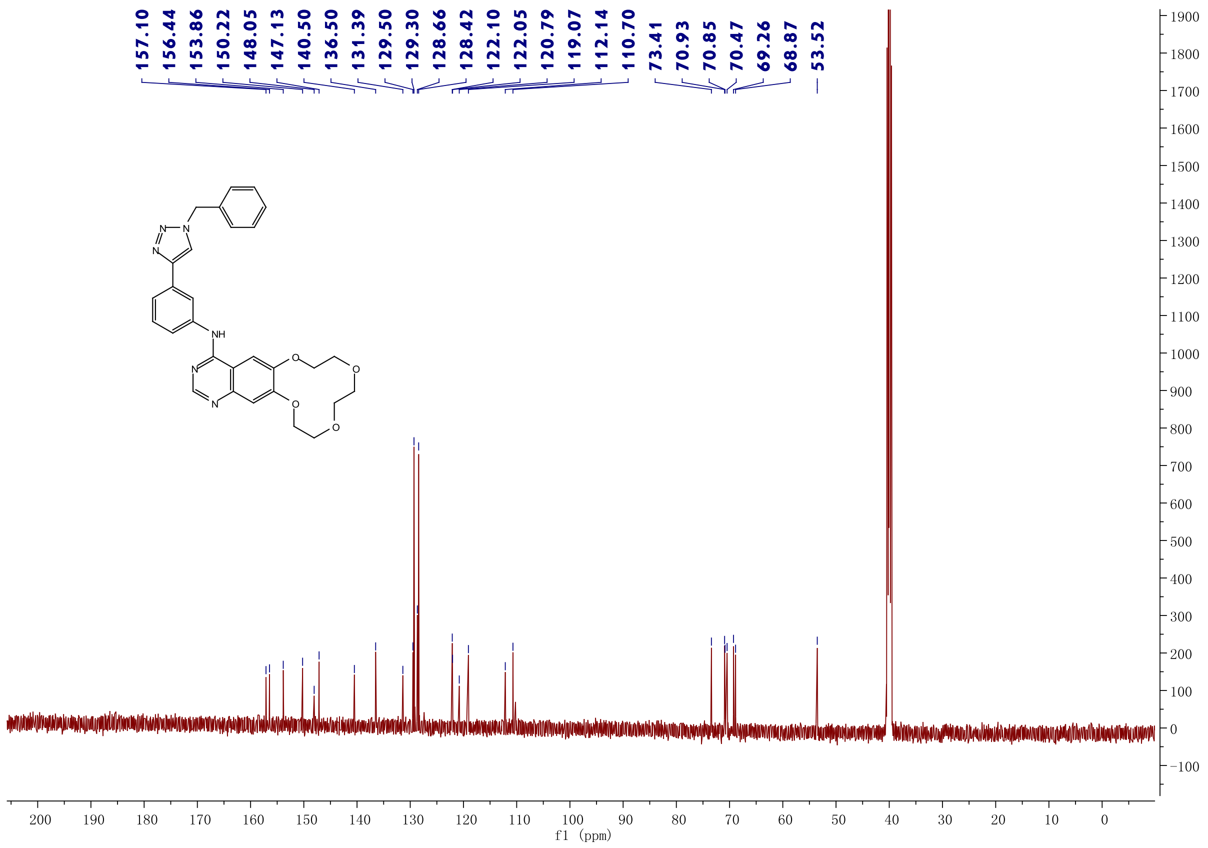

Figure S18-3. HR MS of compound 3r

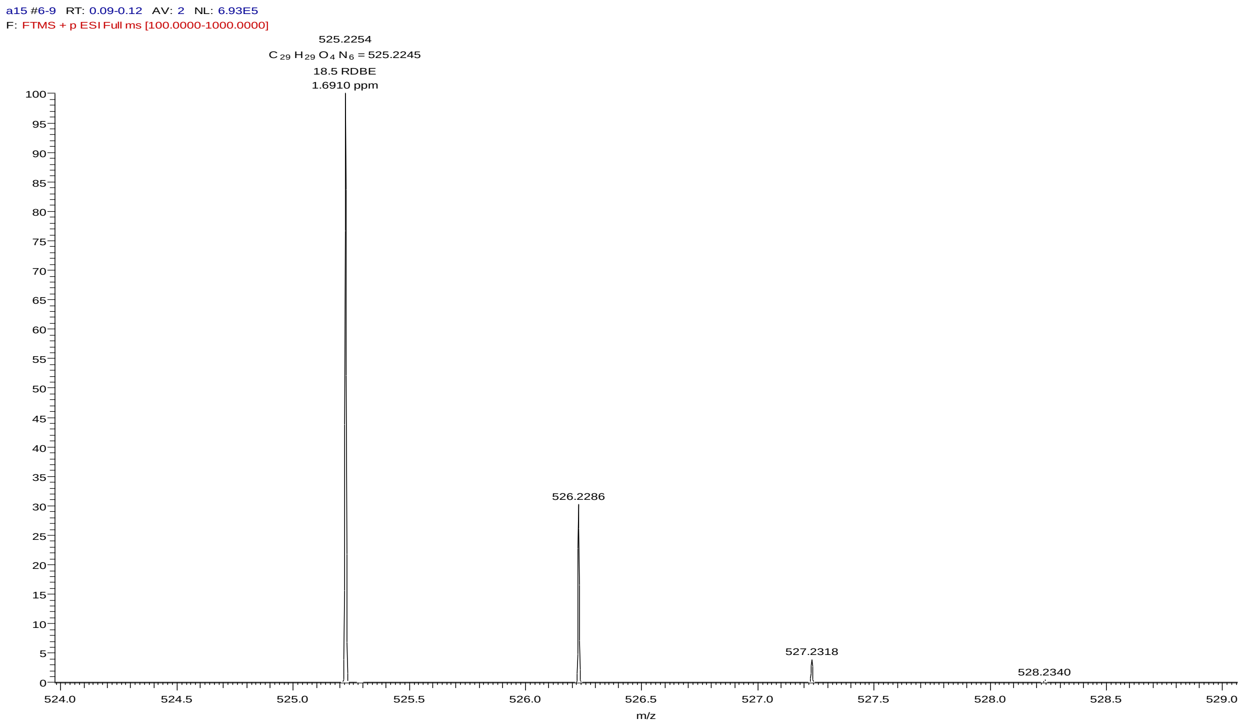

Figure S19-1. ^{1}H NMR spectrum (600 MHz, DMSO-d_6) of compound 3s

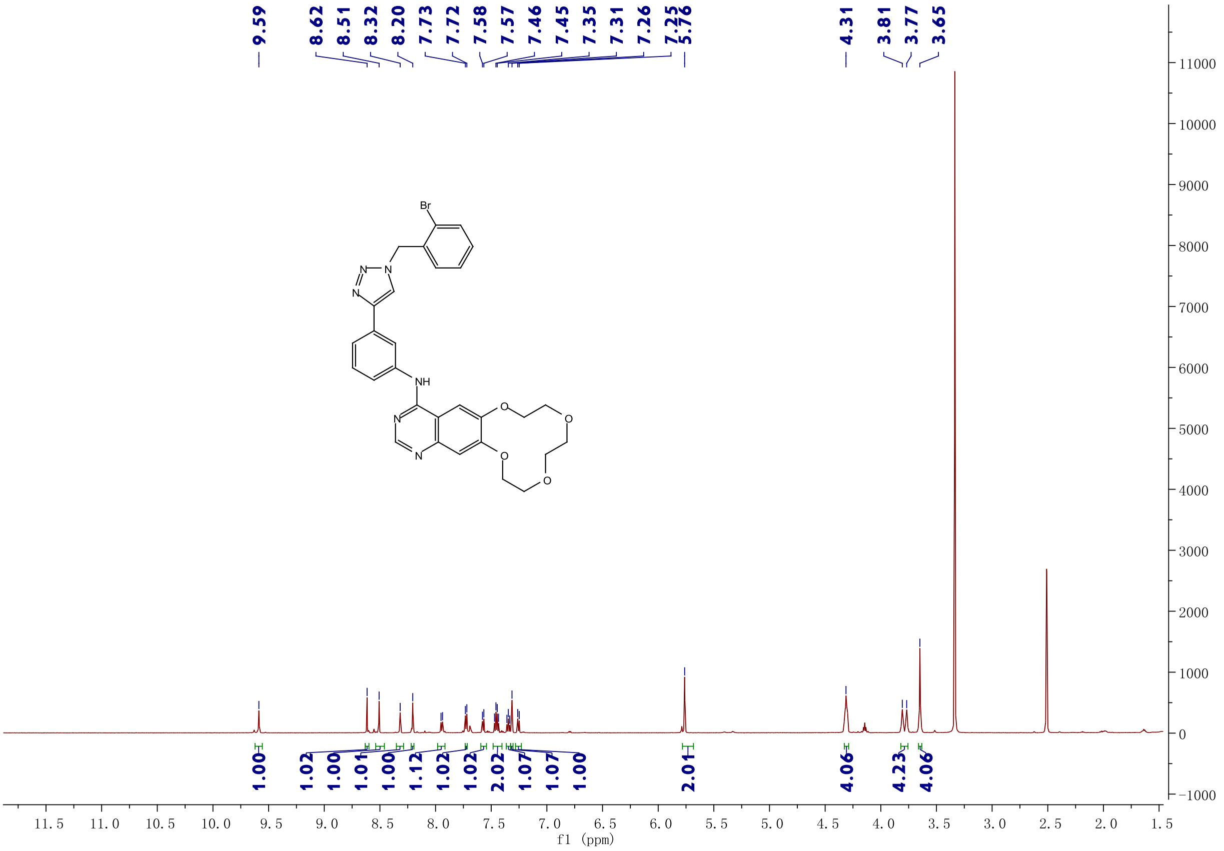

Figure S19-2. HR MS of compound 3s

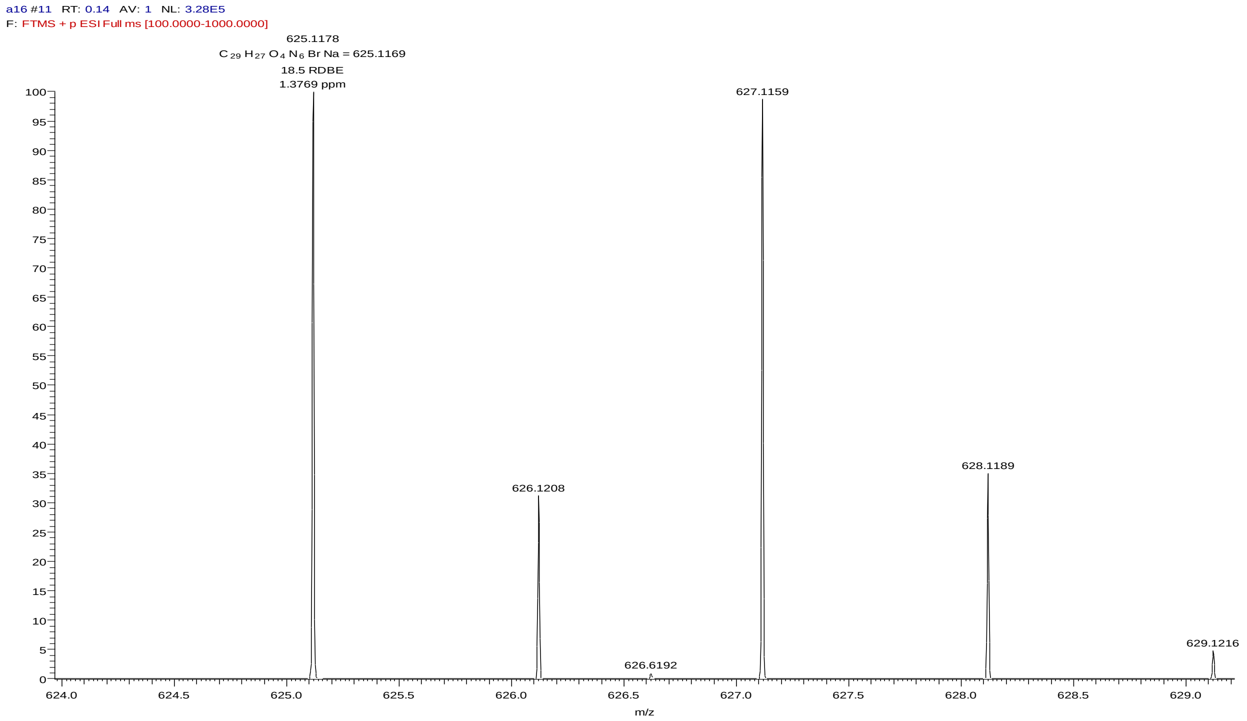

Figure S20-1. ^{1}H NMR spectrum (600 MHz, DMSO-d$_6$) of compound 3t

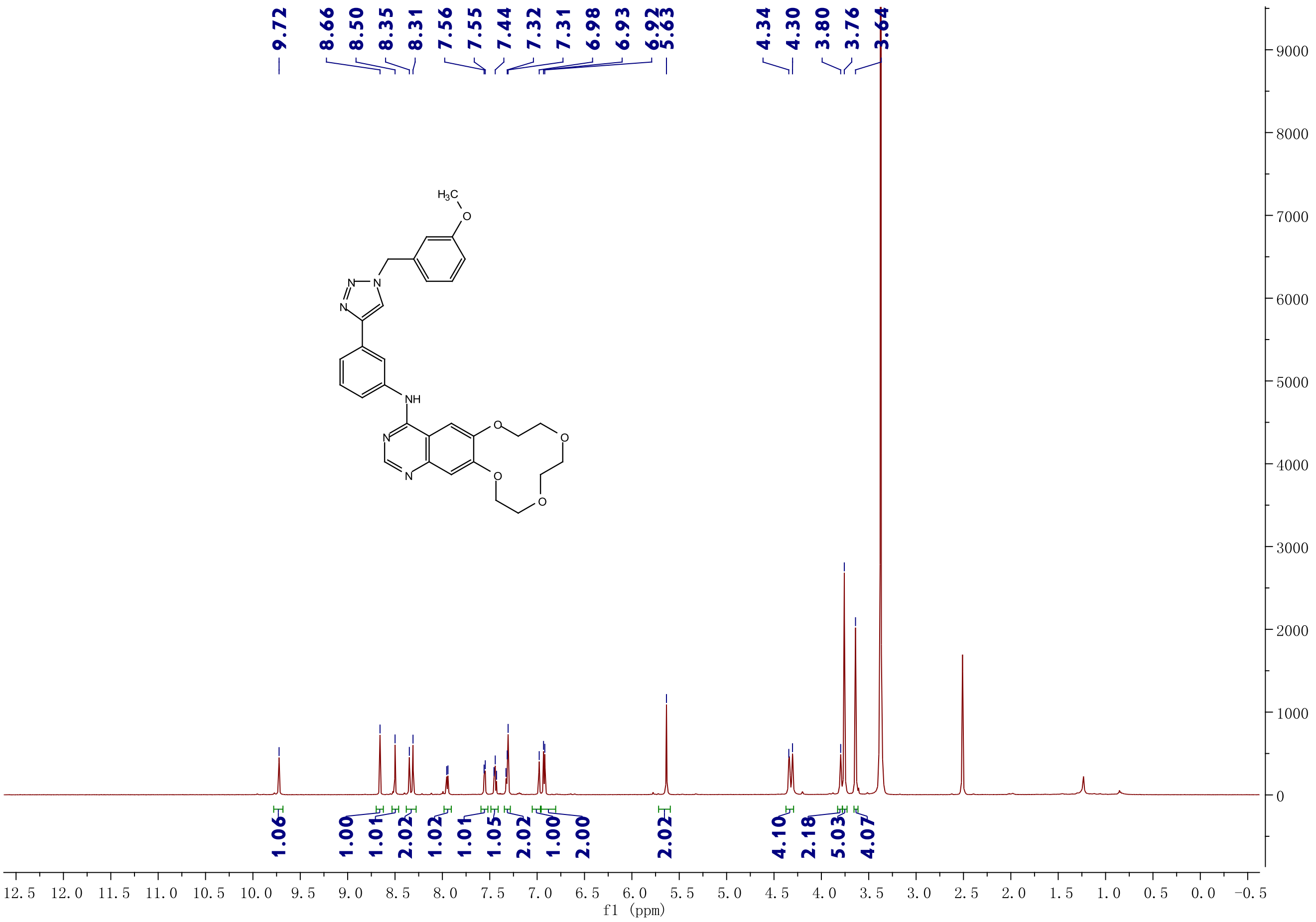

Figure S20-2. ^{13}C NMR spectrum (150 MHz, DMSO-d_6) of compound 3t

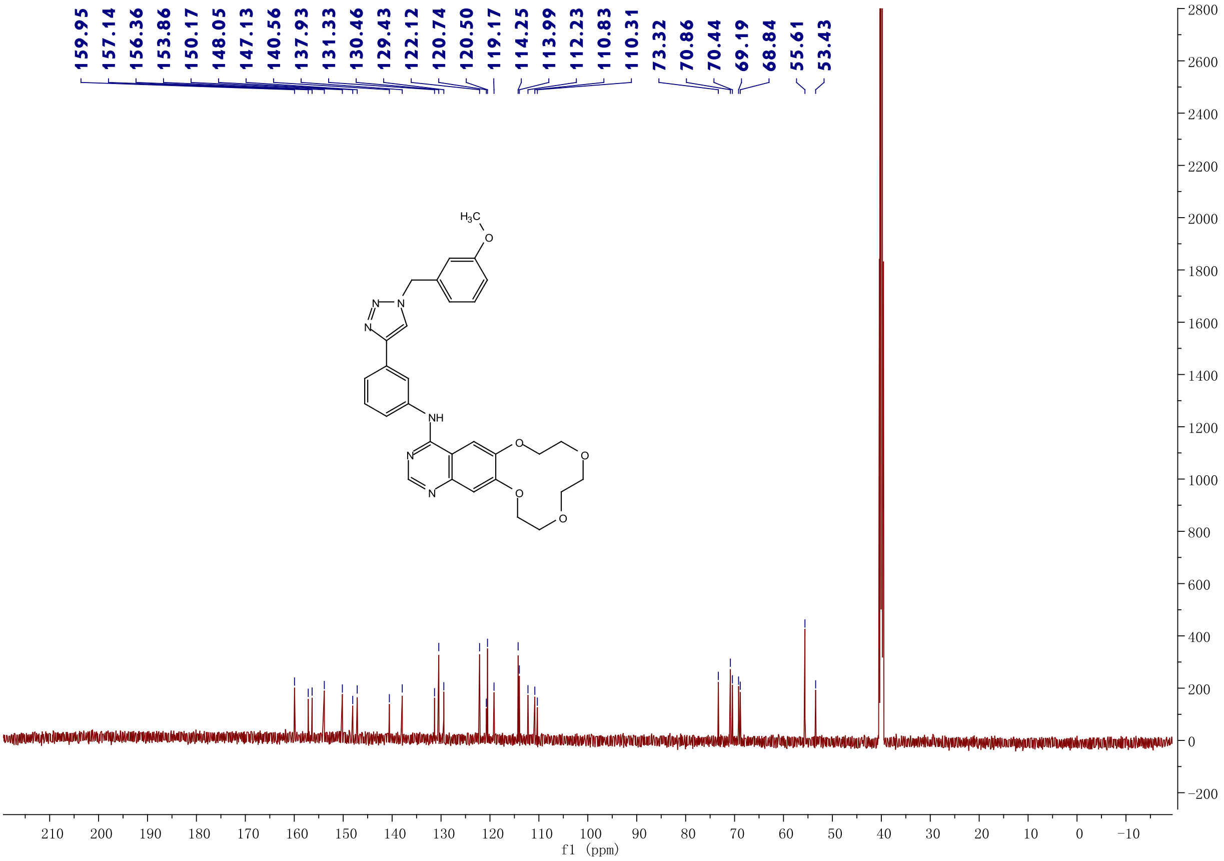

Figure S20-3. HR MS of compound 3t

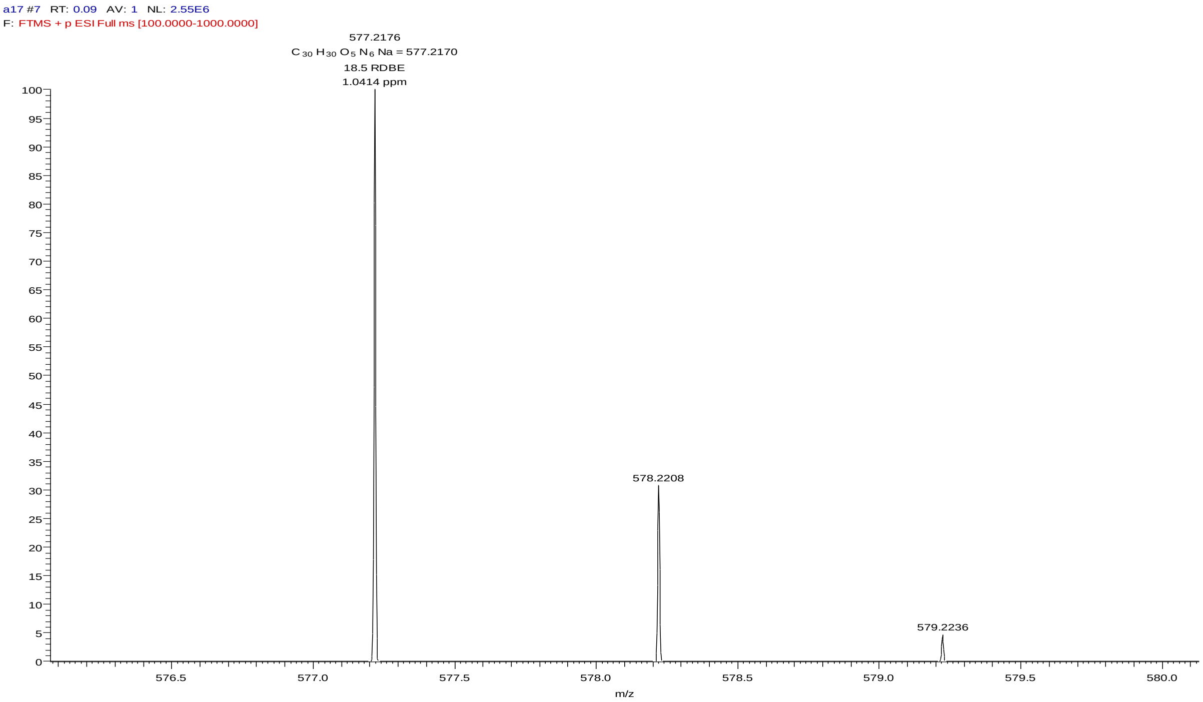

Figure S21-1. ^{1}H NMR spectrum (600 MHz, DMSO-d_6) of compound 3u

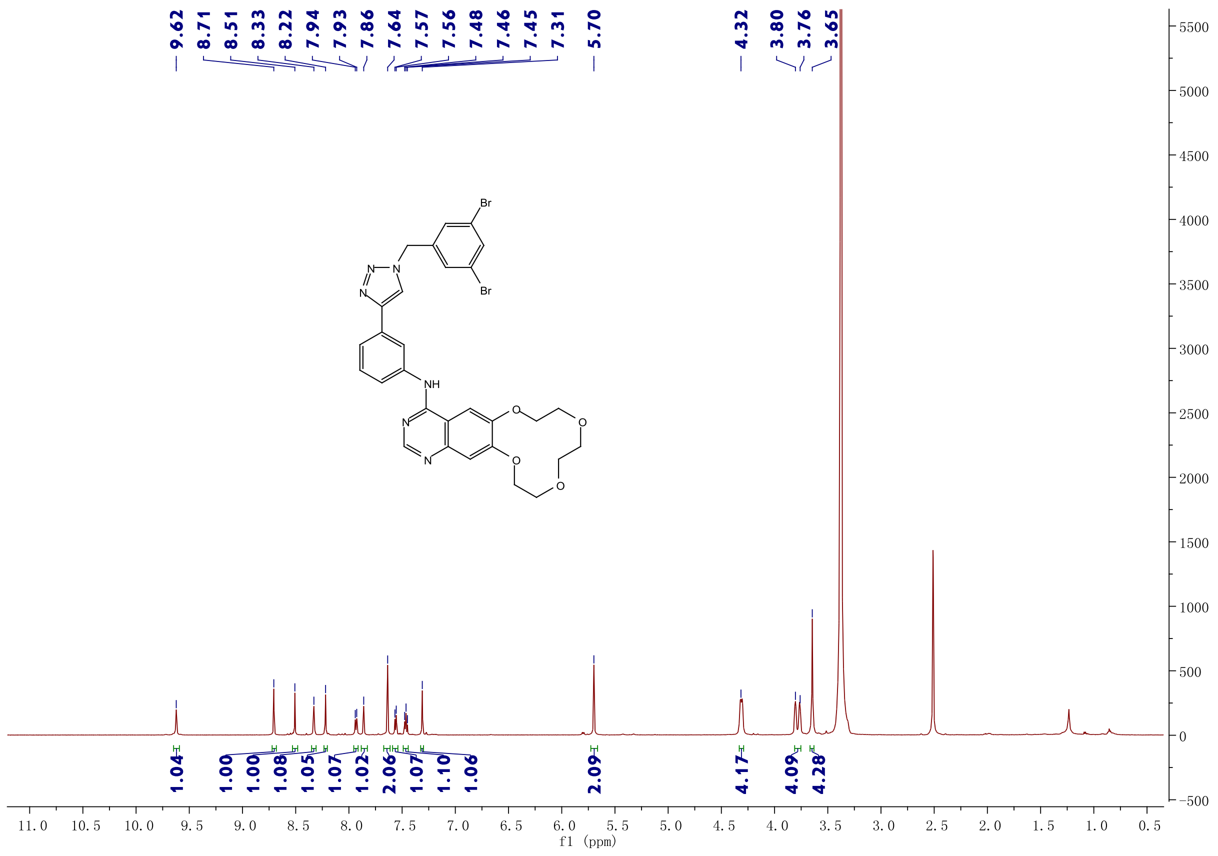

Figure S21-2. ^{13}C NMR spectrum (150 MHz, DMSO-d_6) of compound 3u

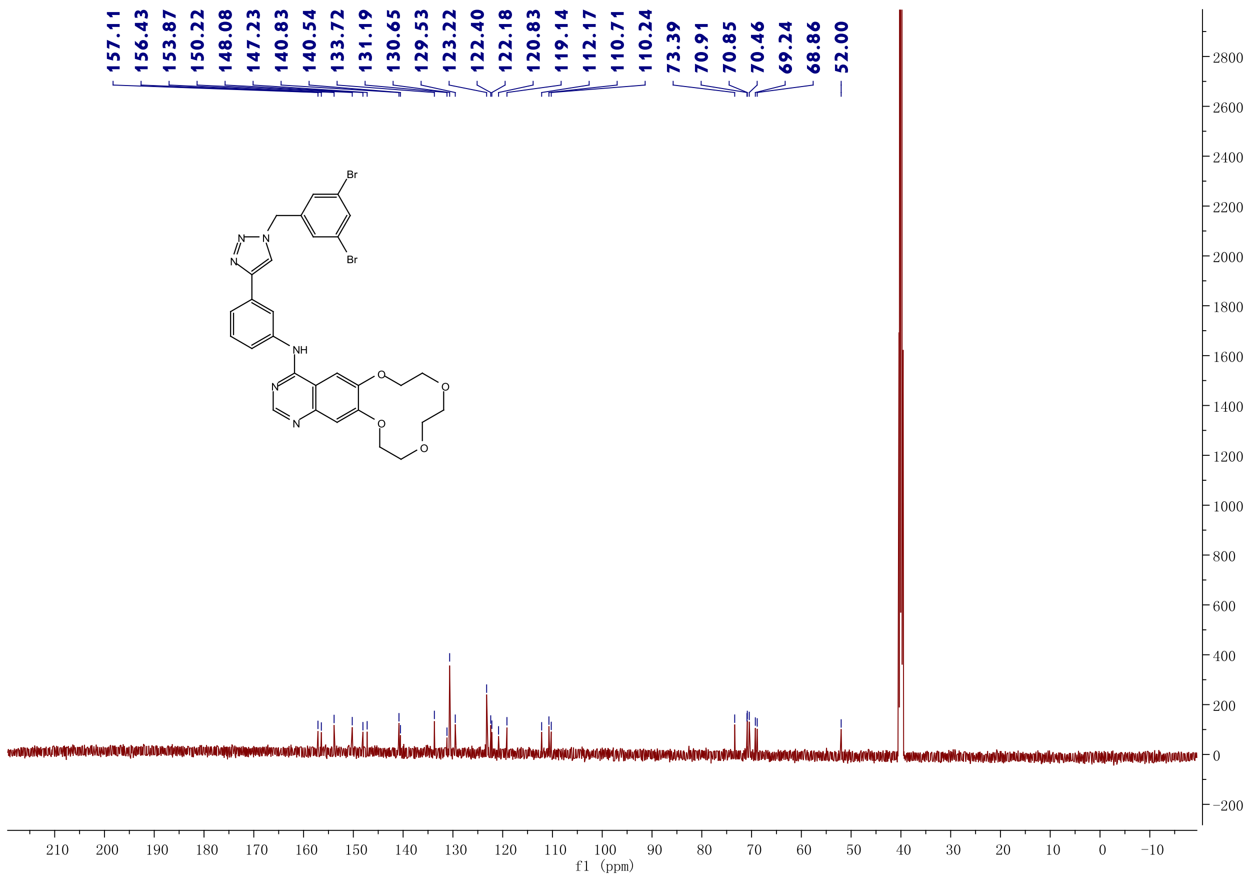

Figure S21-3. HR MS of compound 3u

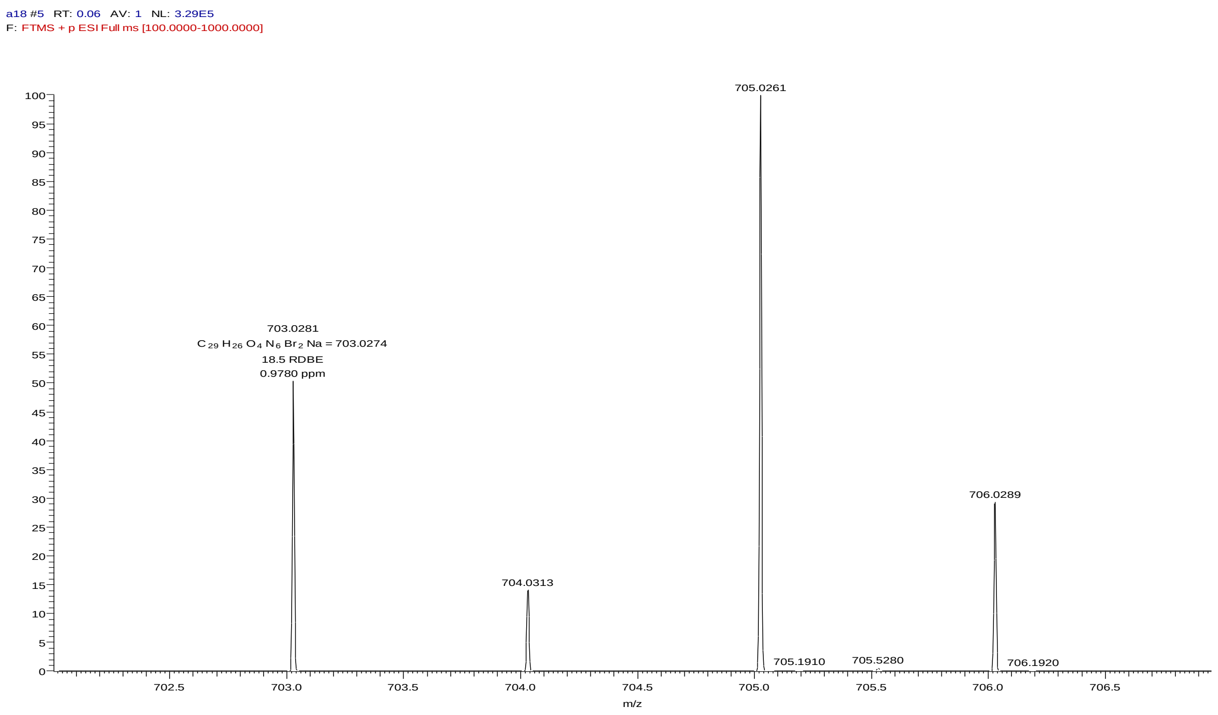

Figure S22-1. ^{1}H NMR spectrum (600 MHz, DMSO-d_6) of compound 3v

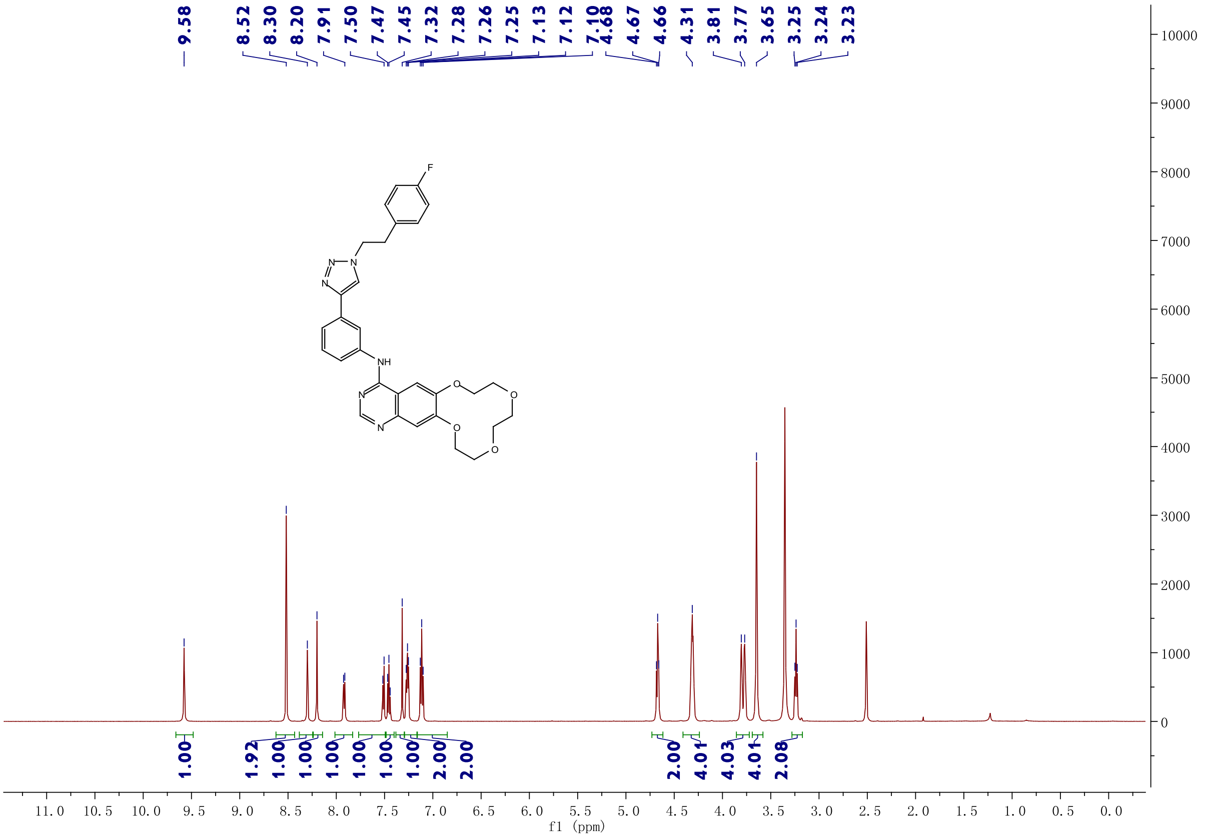

Figure S22-2. ^{13}C NMR spectrum (150 MHz, DMSO-d_6) of compound 3v

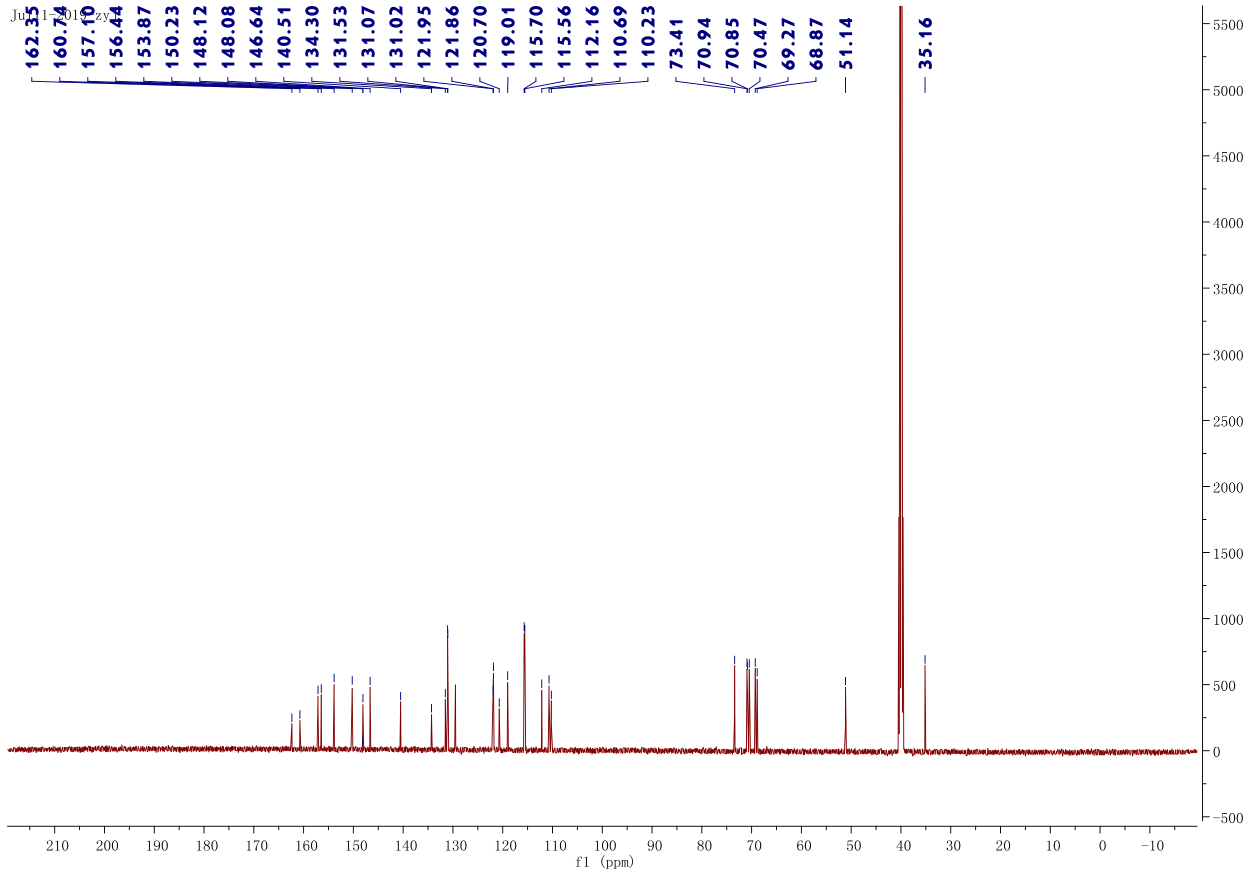

Figure S22-3. HR MS of compound 3v

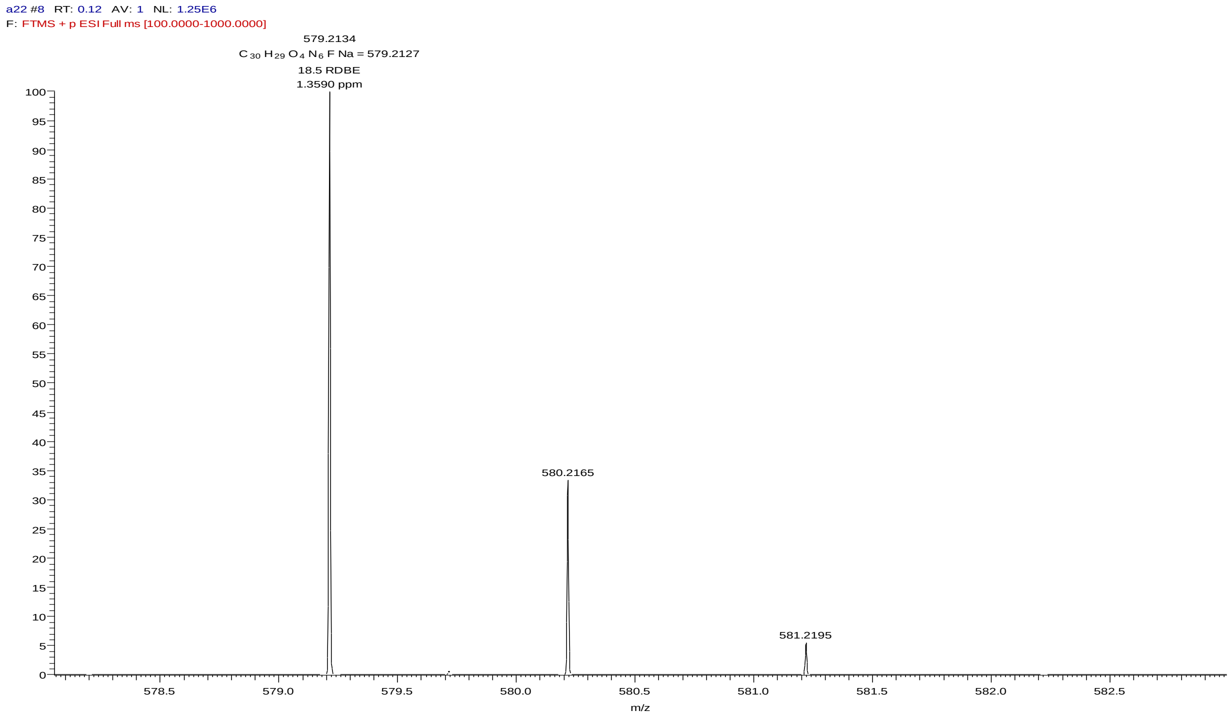

3. Biological studies:

3.1. Plate clone assay

Cells were seeded in 6-well plates with a density of 200 cells/well and cultured overnight for attachment. Cells were exposed to 3l or icotinib of various concentrations (0, 2.5, 5 and 10 μM) separately for 10 days. Medium with or without compounces was changed every 48 hours. When colony formation was visible, the medium was discharged. Then, colonies were washed with cold PBS, fixed with 4% paraformaldehyde (PFA) for at least 30min, and then stained with 0.2% crystal violet solution in 100% ethanol for 20 min.

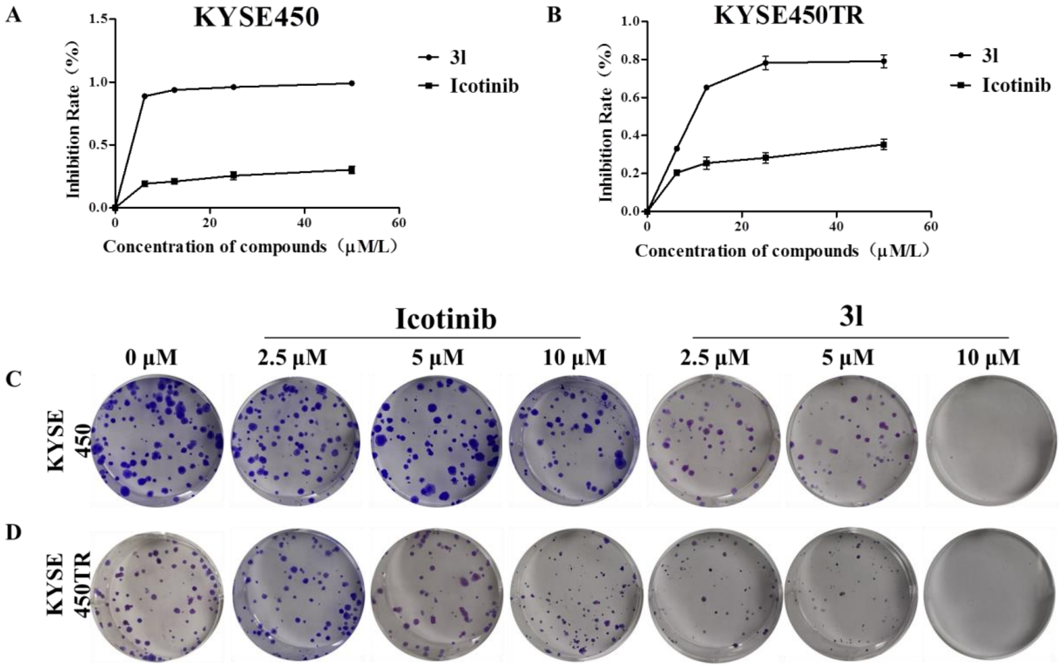

Figure S23. Plate clone assay of icotinib and 3l on cancer cells.

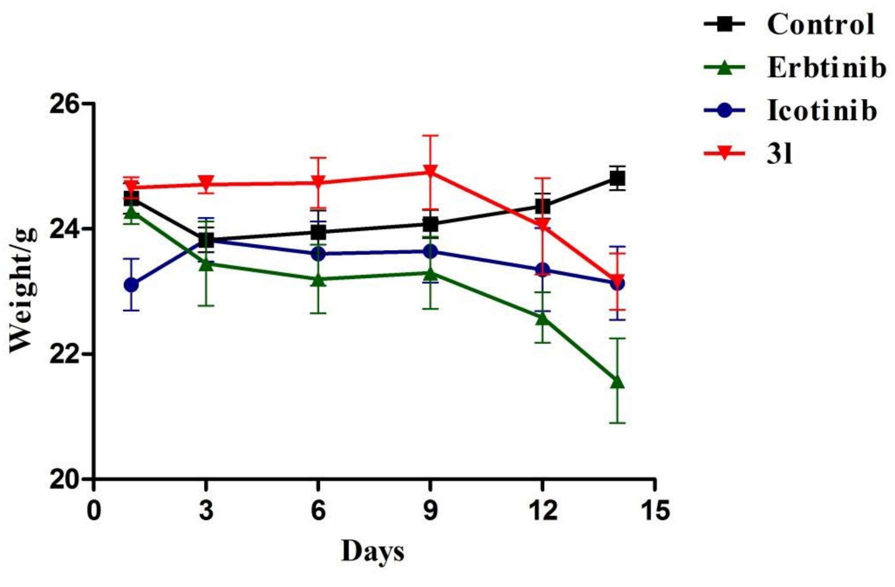

Figure S24. Body weight changes in nude mice during drug administration .
